# *In silico* dysbiosis-associated neuroprotective metabolite insufficiency in Alzheimer’s disease

**DOI:** 10.1515/med-2026-1484

**Published:** 2026-07-25

**Authors:** Nehad A. Shaer, Nouf S. Al-Abbas

**Affiliations:** Department of Biology, Jamoum University College, Umm Al-Qura University, Makkah, Saudi Arabia

**Keywords:** oral–gut–brain axis, ectopic colonization, *Fusobacterium*, *Streptococcus*, therapeutic intervention

## Abstract

Perturbation of oral and gut microbiomes has been implicated in alzheimer’s disease (AD) along the oral-gut-brain axis; however, the extent of global community restructuring may differ between niches. The present work constitutes a computational interrogation of 16S rRNA profiles from eight-month-old APP/PS1 and wild-type mice, characterizing oral and gut community structure and predicted functional capacity through PICRUSt-driven, KEGG-anchored pathway inference. Comparative taxonomic interrogation disclosed patterns consistent with ectopic occurrence, whereby classically oral genera (*Fusobacterium*, *Streptococcus*) were preferentially enriched within intestinal assemblages and gut-typical genera (*Muribaculum*, *Paramuribaculum*) emerged as prominent constituents of the oral microbiota in APP/PS1 mice. These cross-niche enrichment patterns, together with significant oral community separation but non-significant gut beta-diversity separation by ANOSIM, were accompanied by predicted decrements in 85 orally enriched enzymes distributed across 25 KEGG pathways, approximately 40 % of which were associated with the biosynthesis of metabolites with reported neuroprotective properties, including glutathione, acetyl-CoA, succinate, malate, formate, lactate, acetate, and monoterpenoids. Systems-level synthesis of these predictions indicated convergent perturbation of antioxidant defenses, bioenergetic circuitry, and one-carbon metabolism, plausibly reflecting metabolic choke points that may favor cognitive deterioration. Although the oral microbiome showed significant inter-cohort separation, the gut microbiome did not, indicating that gut changes are best viewed as taxon-specific shifts within an otherwise stable community. These observations support a hypothesis-generating framework in which oral dysbiosis, selective gut alterations, and cross-niche enrichment patterns are linked to computationally inferred neuroprotective metabolite insufficiency, highlighting specific taxa, enzymes, and pathways as candidates for biomarker and microbiome-targeted therapeutic development in AD.

## Introduction

Alzheimer’s disease (AD) is a progressive neurodegenerative disorder that is pathologically characterized by the gradual development of cognitive decline, memory impairment, and behavioral disturbances [1], 2]. The core neuropathological hallmarks of AD comprise the extracellular accumulation of amyloid-beta (Aβ) plaques and the intracellular formation of neurofibrillary tangles (NFTs) composed of hyperphosphorylated tau protein [1], 2]. Aggregation of tau protein is closely linked to synaptic dysfunction, neuronal loss, and ultimately widespread brain atrophy [1], 2]. The etiology of AD is widely recognized as complex and multifactorial, shaped by genetic susceptibility, environmental influences, and age-related alterations in neurobiology [1], 3].

The relationship between the gut and oral microbiomes in the context of Alzheimer’s disease (AD) underscores the central importance of the “oral-gut-brain axis” in both disease initiation and progression [4]. These microbial communities interface with the brain through partially overlapping but anatomically distinct communication routes. The gut-brain axis (GBA) constitutes a complex bidirectional network that relies not only on vagus nerve-mediated neural signaling but also on the production and systemic dissemination of diverse microbiota-derived metabolites (e.g., short-chain fatty acids, bile acids, neurotransmitter precursors), as well as hormones and immunomodulatory mediators, all of which can modulate neurological function [5]. In parallel, the oral-brain axis (OBA) operates via cranial nerve pathways, including the trigeminal and olfactory nerves, together with hematogenous dissemination of microorganisms and inflammatory factors, providing a direct conduit through which oral-derived signals may influence brain physiology and pathophysiology [6], [7], [8]. Both the oral and gut microbiomes are thought to contribute to AD pathogenesis through shared mechanisms, notably systemic inflammation, oxidative stress, and disruption of the blood-brain barrier (BBB) [5], 7], 8]. Within this framework, the oral-gut-brain axis (OGBA) has emerged as a useful conceptual model that highlights the bidirectional trafficking of microbial communities between the oral cavity and gastrointestinal tract and their combined impact on neuroinflammation and AD-related processes [8], [9], [10]. This integrated system emphasizes the dynamic contribution of microbial dysbiosis to AD progression, whereby perturbations in the taxonomic structure of one microbial niche can trigger systemic signals that reshape distant microbial ecosystems and potentially aggravate neurodegenerative pathology [11], 12]. Recognition of this intricate microbiome-host network is beginning to provide novel insights into the multifactorial etiology of AD and may help to inform microbiome-targeted therapeutic strategies aimed at mitigating disease progression.

Ectopic colonization, defined as the aberrant occurrence or altered abundance of microorganisms in non-native anatomical sites, is increasingly recognized as a key contributor to microbial dysbiosis [12], 13]. Translocation of oral microbes to the gut can disrupt intestinal barrier function and modulate immune responses [12]. In this new environment, commensals may transition into pathobionts, altering local microbial networks through combined genetic, environmental, and host-mediated influences [11], 12], [14], [15], [16], [17]. This commensal-to-pathobiont shift, shaped by host–microbe interactions, may underlie aspects of Alzheimer’s disease (AD) pathogenesis [1], 12], 13], 18]. Notably, the detection of periodontal pathogens in AD brain tissue supports a potential direct link between oral dysbiosis and neurodegenerative processes [17]. Despite accumulating evidence that the oral cavity and gut are intricately interconnected through bidirectional oral-to-gut and fecal-to-oral microbial transmission, these two microbial ecosystems are infrequently interrogated in a coordinated manner within the same host, and comprehensive data delineating their reciprocal dynamics under non-pathological conditions remain comparatively sparse [19].

Despite growing recognition of the oral–gut–brain axis in Alzheimer’s disease, the reciprocal dynamics between the oral and gut microbiomes within the same host remain insufficiently characterized, particularly with respect to whether cross-niche dysbiosis is associated with altered microbial metabolic potential relevant to neurodegeneration. This gap is important because microbiome-derived metabolites may influence oxidative balance, bioenergetics, and inflammatory regulation, all of which are implicated in AD pathophysiology. We therefore hypothesized that APP/PS1 mice exhibit putative bidirectional ectopic occurrence between the oral and gut microbiomes, and that the dysbiotic cross-niche enrichment pattern is associated with reduced predicted biosynthetic capacity for neuroprotective metabolites. Accordingly, the research question addressed in this study was whether oral dysbiosis, selective gut taxonomic and functional alterations, and cross-niche enrichment patterns in APP/PS1 mice are associated with a predicted insufficiency of neuroprotective metabolite synthesis relevant to Alzheimer’s disease progression.

In light of these gaps, we conducted an *in silico* re-analysis of oral and gut microbiomes from APP/PS1 and wild-type mice to test whether cross-niche dysbiosis is associated with altered microbial metabolic potential in Alzheimer’s disease. We hypothesized that APP/PS1 mice would show oral dysbiosis, selective gut shifts, and ectopic enrichment patterns linked to a reduced microbiome-associated capacity to produce neuroprotective metabolites. Accordingly, our central research question was whether oral and gut microbiome alterations in APP/PS1 mice are associated with a computationally inferred insufficiency of neuroprotective metabolite synthesis along the oral–gut–brain axis.

## Materials and methods

### Sample collection and deep sequencing

This *in silico* investigation employed publicly available paired-end 16S rRNA gene (V3–V4 region) sequencing data published by Zhang et al. [20] to examine the oral and gut microbiomes of eight-month-old male transgenic Alzheimer’s disease mice (APPswe/PS1dE9; APP/PS1) in comparison with age-matched wild-type (C57BL/6) male mice. The datasets, originally generated by OE biotech Co., Ltd. (Shanghai, China), were retrieved from the NCBI bioProject database (accession: PRJNA687556). Oral microbiome data included samples SRR13371337–SRR13371349 and SRR13371351 for wild-type mice and SRR13371352–SRR13371365 for APP/PS1 mice, while gut microbiome data consisted of samples SRR13371363–SRR13371375 and SRR13371377 for wild-type mice and SRR13371378–SRR13371391 for APP/PS1 mice, as previously described [20]. The animal experiments in the original study were carried out in accordance with MDL Biomedical Technology regulations and were approved by the Animal Ethics Committee of the Beijing Agro-Biotechnology Research Center (authorization BABRC20190101).

### Bioinformatics analysis

#### Data processing and OTU analysis

The clean paired-end read datasets were retrieved from the NCBI database and subjected to computational re-analysis at the Beijing Genome Institute (BGI), Hong Kong, China. Sequence data processing was performed using QIIME software (version 1.7.0) [21]. The resulting high-quality tags were aligned to the SILVA reference database (http://www.arb-silva.de/), and putative chimeric reads were identified and filtered out using the UCHIME algorithm [22]. The non-chimeric, high-quality reads were then clustered into operational taxonomic units (OTUs) through UPARSE (version 7.0.1090) [23], from which representative sequences were selected for downstream taxonomic annotation [24]. Taxonomic classification was inferred across standard hierarchical levels, with particular focus on the phylum, genus, and species levels. Multiple sequence alignments of representative sequences were generated using MUSCLE software (version 3.8.31) [25].

#### Diversity analyses

Partial least squares-discriminant analysis (PLS-DA) was performed using R software version 2.15.3 as an exploratory, descriptive visualization to illustrate potential cohort-related patterns in the oral and gut microbiome data, without serving as the basis for formal statistical inference. Alpha diversity metrics, including observed species (Sobs), Chao1, and ACE indices, were computed to estimate species richness across the oral and gut microbiome datasets. The Sobs index reflects abundant taxa, Chao1 estimates rare taxa, and ACE provides an integrated estimate for both [26]. A species accumulation curve (Specaccum) was generated using the *vegan* R package to evaluate taxonomic coverage and sequencing depth, where a plateau in the curve was interpreted as an indication of sufficient read depth to capture most taxa [27]. Preliminary beta diversity assessments included rarefaction analysis and the Analysis of Similarities (Anosim). Alpha diversity indices (Sobs, Chao1, ACE) were compared between WT and APP/PS1 cohorts using appropriate two-group statistical tests, with two-sided p values below 0.05 regarded as statistically significant. Beta-diversity differences between cohorts were assessed using ANOSIM applied to weighted UniFrac distance matrices, and the corresponding ANOSIM R statistics and p values were calculated and reported.

Rarefaction curves were generated to evaluate sequencing depth consistency and identify the minimum number of reads shared across the oral and gut microbiome datasets [28]. Anosim was performed using the *anosim* function within the *vegan* R package to examine potential differences in beta diversity between cohorts, based on mean ranked dissimilarities between and within groups [29]. Weighted UniFrac metrics were subsequently applied to incorporate phylogenetic relationships and relative abundance profiles, enabling comparative assessment of microbial community structures across the two microbiome types [30], 31].

#### Functional network in oral microbiome and AD

Functional prediction was carried out using the Phylogenetic Investigation of Communities by Reconstruction of Unobserved States (PICRUSt) software package (version 1.1.4; http://picrust.github.io/picrust/) [32]. The Kyoto Encyclopedia of Genes and Genomes (KEGG) resource (release 115.0, 2025) was employed as the reference framework for a differential enzymatic profiling of the two oral microbiome cohorts. Enzyme selection was based on a set of stringent, predefined criteria: a high inferred abundance threshold (≥6,000) to capture enzymes with substantial representation in the oral microbiome; a clear reduction in inferred abundance ≥0.2-fold in age-matched transgenic male littermates expressing amyloid precursor protein/presenilin-1 (APP/PS1) compared with their wild-type (WT) counterparts; documented integration within at least one KEGG pathway to support putative functional relevance within established biochemical networks; and low inferred abundance (<3,000) or complete absence in the gut microbiome of the same animals. An abundance threshold of ≥6,000 inferred queries in the oral microbiome was selected *a priori* to restrict downstream analysis to enzymes with substantial predicted representation and to minimize the influence of low-abundance, potentially less reliable predictions [33]. Additionally, inferred enzyme abundance data were compared between WT and APP/PS1 cohorts using appropriate two-group statistical tests, and p values were subsequently adjusted for multiple comparisons by controlling the false discovery rate (FDR) according to the Benjamini–Hochberg procedure, with FDR-adjusted q values <0.05 considered statistically significant [34], 35]. It is important to note that the functional inference in this study relied on an OTU-based 16S rRNA processing pipeline (QIIME 1.7 with 97 % OTU clustering) and PICRUSt version 1.1.4, as implemented by the original provider, e.g., Beijing Genome Institute (BGI), Hong Kong, China. These approaches pre-date currently recommended ASV-based workflows (e.g. DADA2 or Deblur) and the more recent PICRUSt2 framework, which provide finer taxonomic resolution and leverage an expanded reference genome catalogue.

#### NSTI calculation and quality control of PICRUSt predictions

Weighted Nearest Sequenced Taxon Index (NSTI) scores were used to evaluate the reliability of PICRUSt-based functional predictions for the oral microbiomes of wild-type and APP/PS1 transgenic male mice [32], 33]. PICRUSt computes weighted NSTI as the relative-abundance–weighted average phylogenetic distance between each amplicon sequence variant (ASV) in a sample and its closest sequenced reference genome on the fixed PICRUSt 16S rRNA reference phylogeny [33], 36]. For each ASV, PICRUSt first determines the branch-length distance to the closest reference genome tip, then multiplies this distance by the ASV’s relative abundance in the corresponding sample, and finally sums these products across all ASVs to obtain a single weighted NSTI score per sample that reflects overall reference genome coverage.

### Ethical statement

All *in vivo* procedures described in the primary study were implemented in strict adherence to the regulatory framework of MDL Biomedical Technology and received formal ethical authorization from the Animal Ethics Committee of the Beijing Agro-Biotechnology Research Center (approval BABRC20190101) by the original investigating team.

## Results

### Numerical analysis and validation of microbiome sequencing datasets

Numerical datasets delineating the composition and diversity of the gut microbiome, derived from eight-month-old wild type male mice (WT) and age-matched transgenic male littermates expressing amyloid precursor protein/presenilin-1 (APP/PS1), have been recently described [37], whereas corresponding data for the oral microbiome are summarized in Table S1. The oral microbiome dataset comprised 28 samples, yielding an aggregate of 2,102,232 clean paired-end reads, with an average of approximately 75,080 reads per sample. Among WT samples, the maximum and minimum clean read counts were 85,667 and 51,600, respectively. The number of operational taxonomic units (OTUs) ranged from 1,820 in APP/PS1 mice to 556 in WT mice. The species accumulation curve (Figure S1) indicated approximately 4,100 distinct OTUs in the oral microbiome, compared with 4,575 OTUs inferred from the gut microbiome in the prior analysis [37]. Both datasets demonstrated sufficient sequencing coverage, suggesting that the majority of microbial taxa present in the oral and gut environments were effectively captured through the computational re-analysis.

Comparative analysis of the ANOSIM plots between the two microbiome datasets suggested that inter-cohort variation (WT vs. APP/PS1) within the oral microbiome was more pronounced than intra-cohort variation (oral ANOSIM R=0.423, p=0.001), whereas no statistically significant differences were inferred between cohorts in the gut microbiome (gut ANOSIM R=0.021, p=0.26; Figure S2) Nevertheless, the gut dataset showed significant richness differences and potentially meaningful taxon-specific shifts, indicating that gut alterations, if present, are better interpreted as selective compositional changes rather than whole-community restructuring. Species richness, representing alpha diversity, displayed greater variability in the APP/PS1 cohort of the oral microbiome compared to the corresponding gut microbiome (Figure S3). Among the alpha diversity indices, Chao1 and ACE showed statistically significant differences between WT and APP/PS1 cohorts in the oral microbiome (p=0.003 and p=0.011, respectively), whereas Sobs showed a trend (p=0.051); in the gut microbiome, all three indices (Sobs, Chao1, ACE) were significantly different between cohorts (p=0.003, p=0.005, and p=0.003, respectively; (Figure S3, Table S2). As expected, overall species richness, independent of cohort, was higher in the gut microbiome than in the oral dataset (Figure S3). These observations were further supported by the rarefaction curve analysis, where sequencing depth was estimated to reach saturation at approximately 51,600 reads for the oral microbiome and 55,000 reads for the gut microbiome (Figure S4), suggesting adequate coverage of microbial diversity across both datasets.

**Figure 1:**
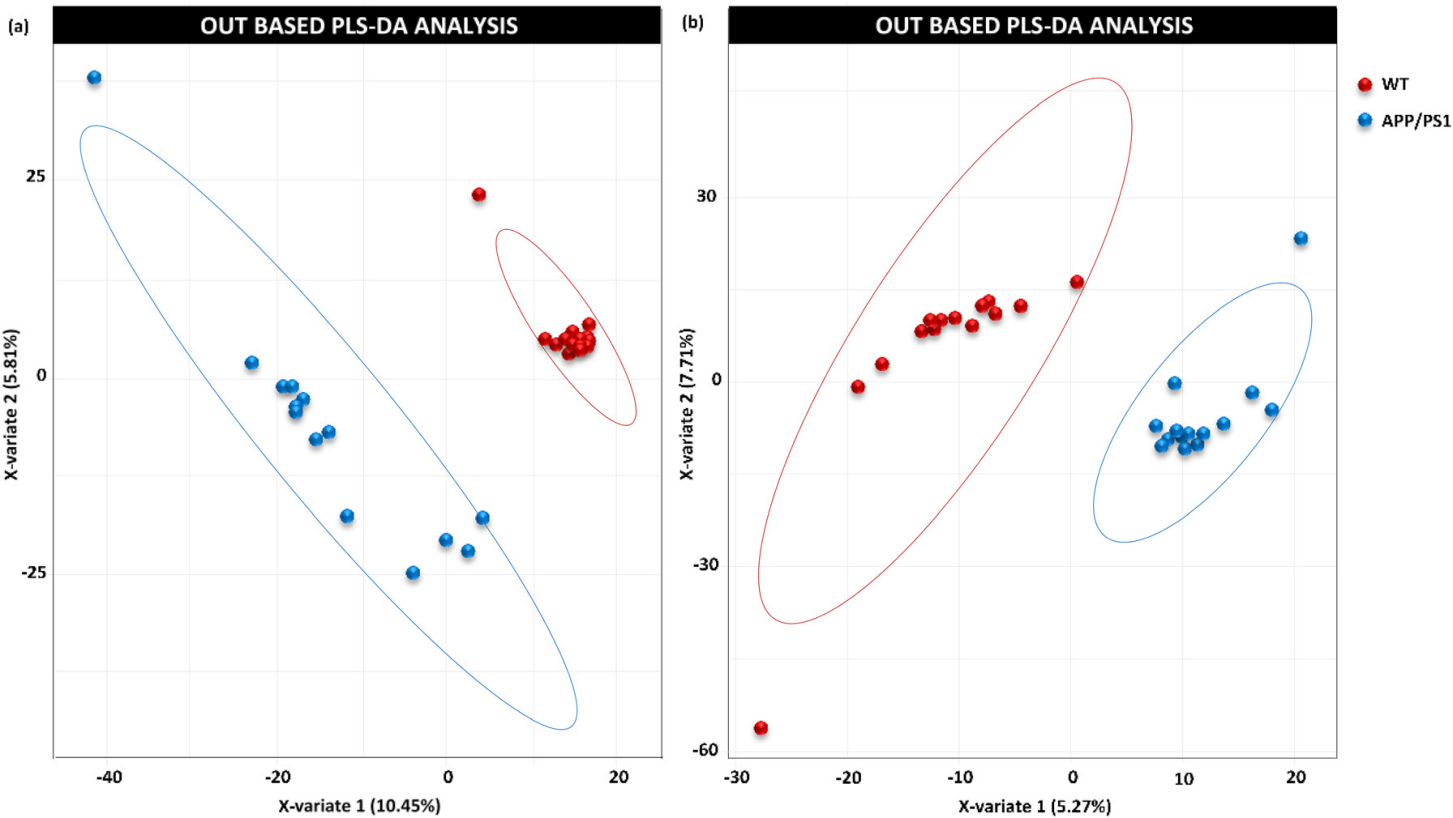
Partial least squares discriminant analysis (PLS-DA) of the oral (a) and gut (b) microbiomes of wild type male mice (WT) and age-matched transgenic littermates expressing the amyloid precursor protein/presenilin-1 (APP/PS1) genes. The APP/PS1 transgenic mice serve as a model for early-onset Alzheimer’s disease (EOAD). PLS-DA plots are shown for descriptive purposes only; statistical evidence for cohort differences is based on UniFrac-based beta-diversity metrics, ANOSIM, and alpha-diversity/taxon-level comparisons rather than on supervised ordination.

Partial least squares-discriminant analysis (PLS-DA) was applied to the oral and gut microbiome datasets to provide an additional exploratory visualization of cohort-related patterns (Figure 1), complementing the UniFrac-based beta-diversity metrics and ANOSIM results. This multivariate visualization was consistent with a clearer separation between APP/PS1 and WT cohorts in the oral microbiome than in the gut microbiome, in line with the unsupervised beta-diversity and alpha-diversity findings, but it was not used as an independent test of group differences. The ordination patterns also appeared compatible with the alpha diversity trends, indicating relatively greater dispersion within the APP/PS1 cohort compared to the corresponding WT cohort for both microbiome types (Figure S3). In the oral microbiome, inferred microbial signatures corresponding to APP/PS1 mice predominantly clustered along the negative side of the X-variates 1 and 2 axes, whereas WT samples were mainly positioned in the positive quadrant of these axes (Figure 1a), suggesting more constrained community structure among WT profiles. A similar but axis-shifted pattern was observed in the gut microbiome, where APP/PS1-associated signatures tended to aggregate along the negative side of X-variate 1 and the positive side of X-variate 2, while WT signatures displayed an approximately inverse distribution (Figure 1b). Taken together, these PLS-DA score plots are consistent with distinct microbial community configurations in APP/PS1 versus WT cohorts in oral microbiome dataset.

### Cross-niche enrichment patterns consistent with ectopic occurrence inferred in APP/PS1 oral microbiomes

**Figure 2:**
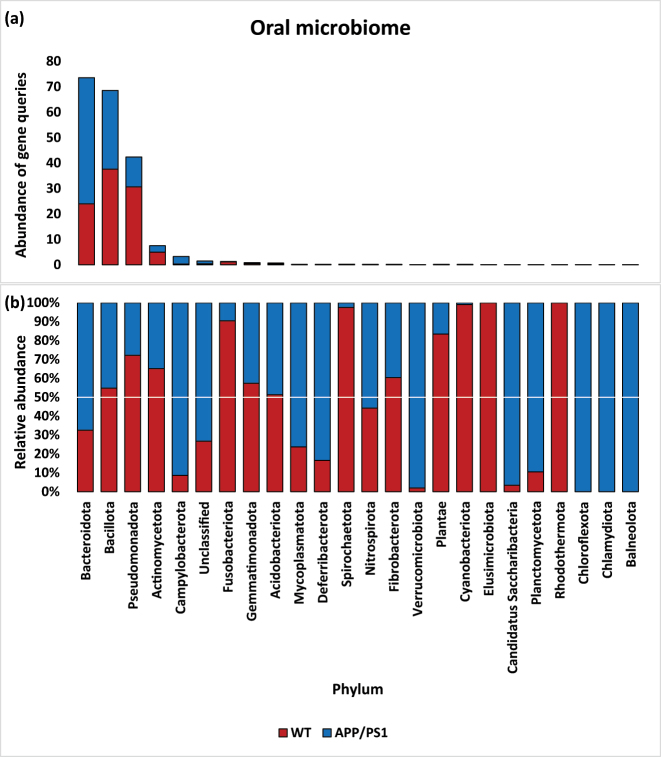
Microbial abundance (a) and relative abundance (b) delineating the microbial composition at the phylum level within the oral microbiome of wild type male mice (WT) and age-matched transgenic male littermates expressing amyloid precursor protein/presenilin-1 (APP/PS1). Additional details can be referenced in Table S4.

**Figure 3:**
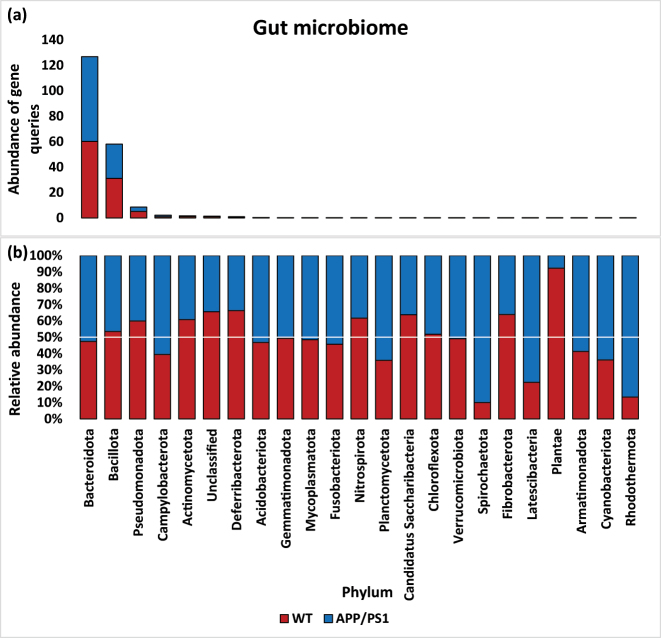
Microbial abundance (a) and relative abundance (b) delineating the microbial composition at the phylum level within the gut microbiome of wild type male mice (WT) and age-matched transgenic male littermates expressing amyloid precursor protein/presenilin-1 (APP/PS1). Additional details can be referenced in Table S5.

**Figure 4:**
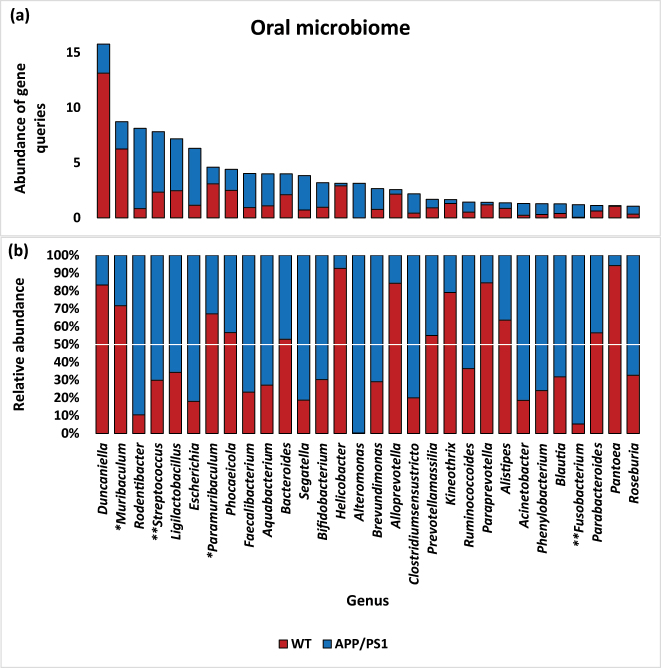
Microbial abundance (a) and relative abundance (b), showcasing the compositional distribution of the top 30 microbial taxa at the genus level within the oral microbiome of wild type male mice (WT) and age-matched transgenic male littermates expressing amyloid precursor protein/presenilin-1 (APP/PS1). The asterisks denote genera that potentially engage in ectopic colonization patterns between distinct microbial ecosystems within the host. Specifically, a single asterisk (*) indicates genera that may migrate from their primary habitat in the gut microbiome to establish a presence in the oral cavity, while a double asterisk (**) signifies genera typically associated with the oral microbiome that might translocate and colonize the gastrointestinal tract. Additional details can be referenced in Tables S6 and S7.

**Figure 5:**
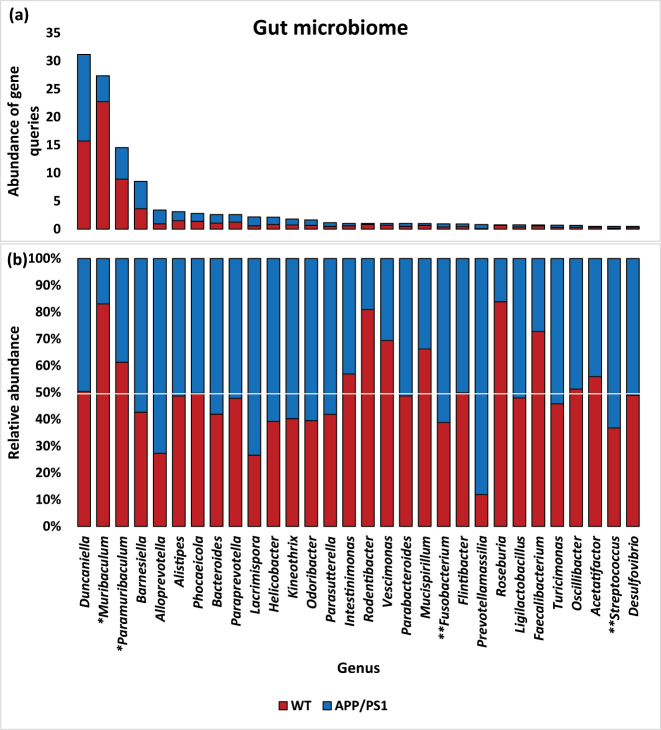
Microbial abundance (a) and relative abundance (b), showcasing the compositional distribution of the top 30 microbial taxa at the genus level within the gut microbiome of wild type male mice (WT) and age-matched transgenic male littermates expressing amyloid precursor protein/presenilin-1 (APP/PS1). The asterisks represent genera that may participate in ectopic colonization between different microbial environments within the host. A single asterisk (*) identifies genera that might relocate from their usual habitat in the gut microbiome to the oral cavity, whereas a double asterisk (**) indicates genera commonly found in the oral microbiome that could migrate and be established in the gastrointestinal tract. Further information regarding these patterns can be found in Tables S6 and S7.

**Figure 6:**
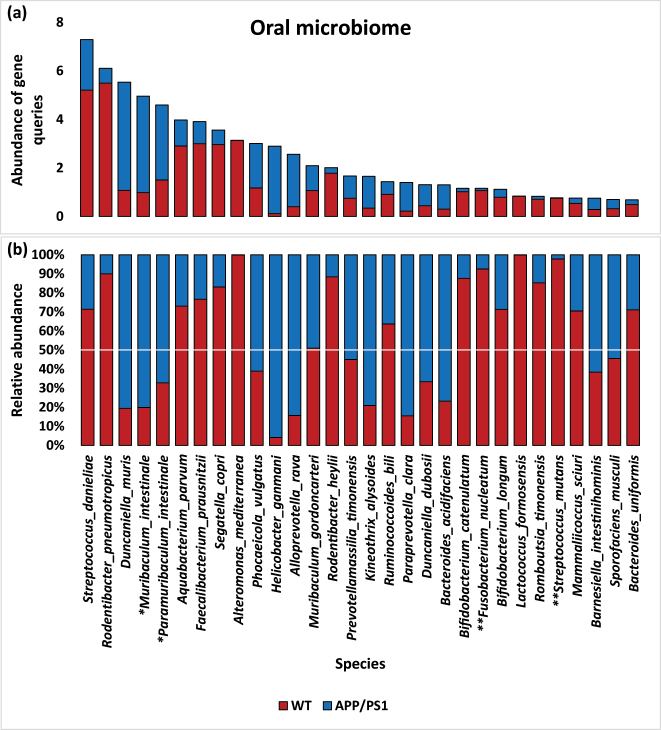
Microbial abundance (a) and relative abundance (b), showcasing the compositional distribution of the top 30 microbial taxa at the species level within the oral microbiome of wild type male mice (WT) and age-matched transgenic male littermates expressing amyloid precursor protein/presenilin-1 (APP/PS1). The asterisks denote species that potentially engage in ectopic colonization patterns between distinct microbial ecosystems within the host. Specifically, a single asterisk (*) indicates species that may migrate from their primary habitat in the gut microbiome to establish a presence in the oral cavity, while a double asterisk (**) signifies species typically associated with the oral microbiome that might translocate and colonize the gastrointestinal tract. Additional details can be referenced in Tables S8 and S9.

**Figure 7:**
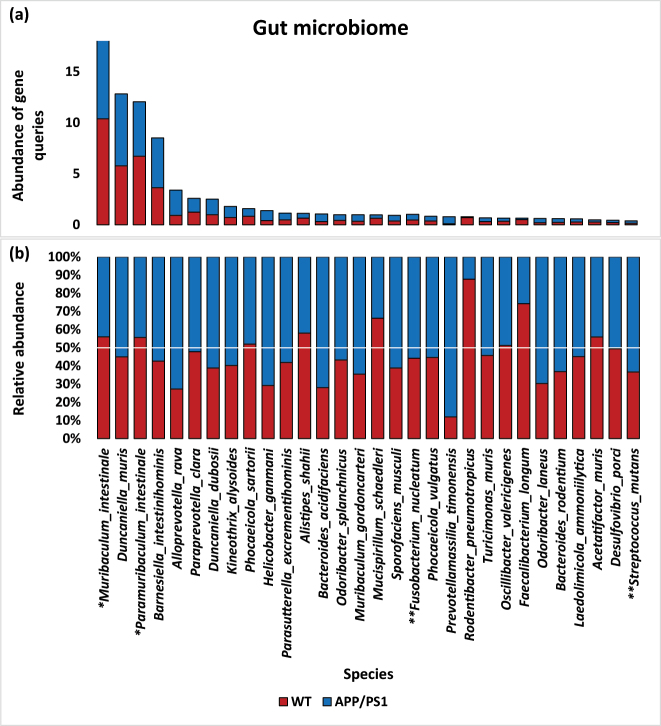
Microbial abundance (a) and relative abundance (b), showcasing the compositional distribution of the top 30 microbial taxa at the species level within the gut microbiome of wild type male mice (WT) and age-matched transgenic male littermates expressing amyloid precursor protein/presenilin-1 (APP/PS1). The asterisks denote species that potentially engage in ectopic colonization patterns between distinct microbial ecosystems within the host. Specifically, a single asterisk (*) indicates species that may migrate from their primary habitat in the gut microbiome to establish a presence in the oral cavity, while a double asterisk (**) signifies species typically associated with the oral microbiome that might translocate and colonize the gastrointestinal tract. Additional details can be referenced in Tables S8 and S9.

Microbial abundance and relative abundance were computationally examined for both the oral and gut microbiomes of wild-type (WT) and amyloid precursor protein/presenilin-1 (APP/PS1) cohorts across multiple taxonomic ranks – phylum (Figures 2 and 3; Tables S4 and S5), genus (Figures 4 and 5; Tables S6 and S7), and species (Figures 6 and 7; Tables S8 and S9). A comparative *in silico* analysis of the two microbiome types was performed to infer potential patterns of ectopic microbial occurrence, possibly reflecting oral-to-gut or gut-to-oral translocation. All phyla detected were included in the assessment, while the thirty most abundant taxa at the genus and species levels were prioritized for detailed evaluation based on relative abundance metrics.

**Figure 8:**
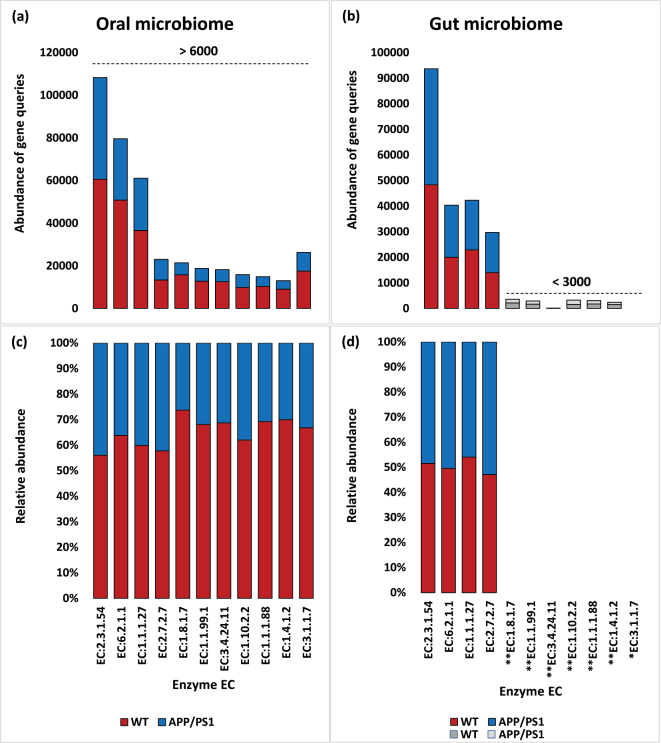
Comparative analysis of 11 specific KEGG enzymes in oral and gut microbiomes, focusing on their functional abundance (a & b) and relative abundances (c & d) in wild-type mice (WT) versus transgenic littermates expressing the APP/PS1 genes associated with Alzheimer’s disease (AD). These enzymes exhibited a significant decrease (≥0.2-fold) in relative abundance in either microbiome of the APP/PS1 transgenic mice compared to that of their WT counterparts. These enzymes were carefully selected based on their previously documented involvement or potential role in AD occurrence and/or pathogenesis, as indicated by existing scientific literature. In the oral microbiome, all 11 enzymes met the threshold of ≥ 6,000 for functional abundance, whereas in the gut microbiome, only four enzymes surpassed the ≥3,000 threshold. The remaining seven gut enzymes either showed no significant difference between WT and APP/PS1 groups (marked with **) or were completely absent (denoted by *). EC: 2.3.1.54=formate C-acetyltransferase; EC: 6.2.1.1=acetate-CoA ligase; EC: 1.1.1.27=L-lactate dehydrogenase; EC: 2.7.2.7=butyrate kinase; EC: 1.8.1.7=glutathione-disulfide reductase; EC: 1.1.99.1=choline dehydrogenase; EC: 3.4.24.11 (K01389)=neprilysin (neutral endopeptidase or NEP); EC: 1.10.2.2 (EC: 7.1.1.8)=quinol--cytochrome-c reductase; EC: 1.1.1.88=Hydroxymethylglutaryl-CoA reductase; EC: 1.4.1.2=glutamate dehydrogenase, EC: 3.1.1.7=acetylcholinesterase. Additional details can be referenced in Supplementary Tables S10-S12.

Across both microbiome types, the predominant phyla identified were Bacteroidota (synonym “Bacteroidetes”), Bacillota (synonym “Firmicutes”), and Pseudomonadota (synonym “Proteobacteria”) in both wild-type (WT) and amyloid precursor protein/presenilin-1 (APP/PS1) cohorts. The relative abundance of Bacillota and Pseudomonadota appeared higher in the WT microbiomes, whereas Bacteroidota was comparatively enriched in the APP/PS1 datasets. Of note, Cyanobacteriota, Elusimicrobiota, and Rhodothermota were virtually undetected in the APP/PS1 oral microbiome (Figure 2; Table S4), while all identified phyla were represented, albeit at varying levels, in the APP/PS1 gut microbiome (Figure 3; Table S5). At the genus level, irrespective of cohort, the most abundant taxa in the oral microbiome were *Duncaniella*, followed by *Muribaculum*, *Rodentibacter*, *Streptococcus*, *Ligilactobacillus*, and *Escherichia* (Figure 2; Table S4). In contrast, the gut microbiome was dominated by *Duncaniella*, followed by *Muribaculum*, *Paramuribaculum*, *Barnesiella*, *Alloprevotella*, and *Alistipes* (Figure 3; Table S5). Notably, the genera *Muribaculum* and *Paramuribaculum*, typically regarded as gut-associated taxa, were among the 30 most abundant genera in the oral microbiome (Figure 4; Table S6), whereas *Fusobacterium* and *Streptococcus*, commonly associated with the oral cavity, were among the abundant genera in the gut microbiome (Figure 5; Table S7), suggesting possible ectopic occurrence across niches. A similar pattern was observed at the species level for these four genera, with *Muribaculum intestinale* and *Paramuribaculum intestinale* detected among the abundant taxa in the oral microbiome (Figure 6; Table S8), and *Fusobacterium nucleatum* and *Streptococcus mutans* identified within the gut microbiome (Figure 7; Table S9). In this *in silico* context, these four instances were interpreted as putative examples of ectopic occurrence between the oral and gut microbiomes, which may be relevant to Alzheimer’s disease (AD)–associated microbial shifts. Further exploratory analyses of these genera and species were undertaken to probe potential mechanisms that could underlie or favor such inferred translocation patterns in the AD setting.

### Functional enzymatic network potentially implicated in alzheimer’s disease

The functional abundance analysis of the oral microbiome inferred the presence of 928 enzymes exceeding a high-abundance threshold of ≥6,000 queries across wild-type (WT) male mice and age-matched transgenic male littermates expressing amyloid precursor protein/presenilin-1 (APP/PS1) (Table S10). In contrast, the gut microbiome displayed a distinct inferred enzymatic profile, with 847 enzymes meeting a high-abundance threshold of ≥3,000 queries across both cohorts (Table S11). The high-abundance cut-off of ≥6,000 inferred queries in the oral microbiome and ≥ 3,000 queries in the gut microbiome across were selected *a priori* as a conservative filter to focus on enzymes with substantial predicted representation and to reduce noise from sparsely inferred functions. This threshold was applied uniformly across cohorts and was not tuned to maximize statistical significance; it provides a pragmatic operational definition of “high abundance” for the present analysis, rather than a biologically absolute boundary, and alternative thresholds or continuous modeling strategies could be explored in future work [33], [34], [35], [36], [37], [38]. From a technical standpoint, these functional profiles were generated using PICRUSt1 applied to 97 % OTU-clustered 16S rRNA data, rather than to ASV-resolved profiles or shotgun metagenomes. Consequently, the reported enzyme and pathway abundances represent *in silico* predictions constrained by a legacy OTU-based pipeline and an earlier generation of the PICRUSt framework, and are best viewed as “hypothesis-generating” patterns that might require confirmation with updated ASV-based analyses, PICRUSt2, or direct metagenomic and metabolomic measurements in future work.

A comparative *in silico* assessment of these highly abundant enzymatic profiles in oral and gut microbiomes led to the identification of 121 enzymes with notable inferred abundance in the oral microbiome that were selected for more detailed analysis (Table S12). This selection was guided by predefined criteria: each enzyme showed a marked reduction in inferred abundance (≥0.2-fold) in APP/PS1 mice relative to WT mice, and was additionally characterized by low inferred abundance (<3,000) or absence in the gut microbiome across cohorts. This distribution pattern points to a putative oral specificity of these enzymes in the Alzheimer’s disease context and enabled a more focused exploration of how shifts in oral enzymatic repertoires might relate to neurodegeneration-associated processes in APP/PS1 mice. The emphasis on high-abundance, pathway-integrated enzymes with a ≥0.2-fold reduction in APP/PS1 mice should not be interpreted as suggesting that low-abundance enzymes lack biological importance; enzymes below these thresholds may still play roles in Alzheimer’s disease-related processes, even if their representation is comparatively limited. Rather, the present analysis should be viewed as highlighting a conservative core network of strongly enriched candidates, while acknowledging that complementary approaches with less stringent filtering or direct metagenomic data will be needed to more fully characterize the contribution of low-abundance enzymatic functions.

The subset of oral enzymes was further refined to 85 candidates, each annotated to one or more KEGG pathways, thereby substantiating their putative integration within established biochemical circuits. This ensemble was then interrogated under a multiple-hypothesis-testing framework using false discovery rate (FDR) control, where FDR is defined as the expected proportion of spurious positives among all results declared significant and was regulated via the Benjamini–Hochberg procedure. The FDR-adjusted statistics in Table S13 demonstrate that these 85 enzymes exhibit consistently higher inferred abundances in the oral microbiome of WT animals relative to APP/PS1 counterparts. Across enzymes, median values differ by several-fold with only limited interquartile overlap, and the associated p-values remain below 0.05 following FDR adjustment. Taken together, these features indicate that the attenuation of this enzymatic cohort in APP/PS1 oral microbiota constitutes a statistically robust signal under stringent multiple-comparison control, thereby legitimizing their designation as a core dysregulated module in the present analysis. This additional filtering step was implemented to enrich enzymes with heightened plausibility of participating in biologically meaningful metabolic and regulatory processes. Within this refined set, 11 enzymes have been previously implicated in the etiology or pathobiology of Alzheimer’s disease (AD) according to published reports (Figure 8). Of these, four maintain relatively elevated inferred abundances (≥3,000 gene queries) within the gut microbiome of both cohorts without exhibiting a ≥0.2-fold decrement in APP/PS1 mice, whereas the remaining seven display low inferred abundance (<3,000 gene queries) across gut samples or are entirely undetected in that compartment, underscoring their apparent oral specificity in the AD context (Figure 8).

**Figure 9:**
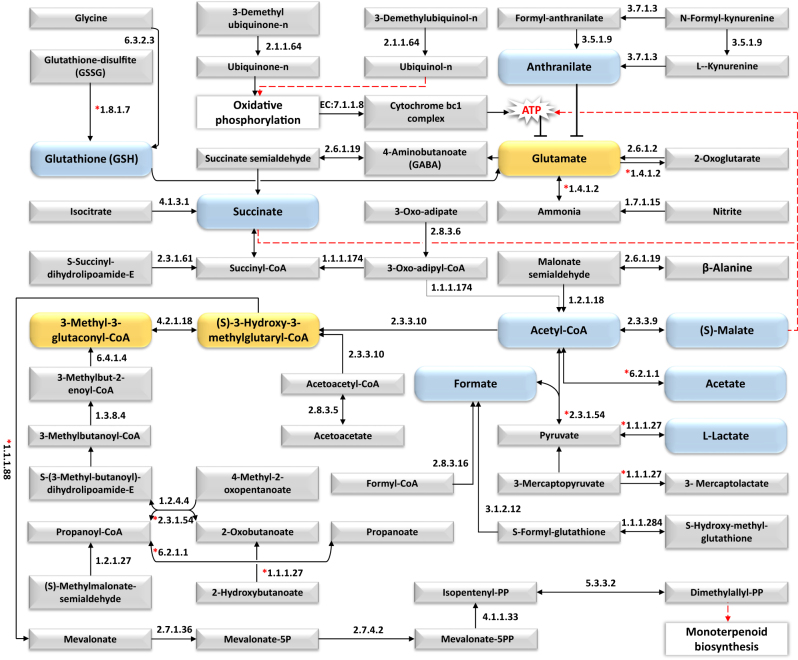
Metabolic network analysis focusing on 33 KEGG enzymes highly enriched in the oral microbiome of wild-type (WT) mice, while exhibit a significant decrease in abundance (≥0.2-fold) in age-matched transgenic male littermates expressing the amyloid precursor protein/presenilin-1 (APP/PS1) genes. None of the illustrated enzymes exhibited elevated abundance in the gut microbiota of wild-type (WT) mice when compared to their APP/PS1 counterparts. A significant portion of these enzymes were entirely absent from the gut microbiome, which led to disconsidering in our latest investigation into the impact of gut microbiome on Alzheimer’s disease (AD). This complex network synthesizes 25 distinct cross-talking KEGG pathways, illustrating the sophisticated interrelationships among various biochemical processes. The diagram also elucidates how these interconnected metabolic routes culminate in the generation of pivotal metabolite that hold significant potential as biomarkers, offering valuable insights into both the occurrence and progression of AD. The final products of these metabolic pathways, including succinate, formate, isoprene, malate, and anthranilate, are highlighted in blue boxes, representing the significant outputs resulting from the altered enzymatic activities observed in APP/PS1 mice. Other crucial metabolic intermediates/end-products, such as acetyl-CoA, glutamate, 3-methyl-3-glutaconyl-CoA, and (S)-3-hydroxy-3-methylglutaryl-CoA, are enclosed in orange boxes. These compounds play vital roles in various biochemical pathways, further elucidating the metabolic disparities between wild-type and APP/PS1 transgenic mice in the context of oral microbiome metabolism in relation to Alzheimer’s disease (AD). EC: 3.7.1.3=kynureninase; EC: 3.5.1.9=arylformamidase; EC: 2.6.1.2=alanine transaminase; *EC: 1.4.1.2=glutamate dehydrogenase; EC: 1.7.1.15=nitrite reductase (NirBD); EC: 2.6.1.19=4-aminobutyrate--2-oxoglutarate transaminase; EC: 1.2.1.18=malonate-semialdehyde dehydrogenase (acetylating); EC: 2.3.3.9=malate synthase; *EC: 6.2.1.1=acetate--CoA ligase; *EC: 1.1.1.27=L-lactate dehydrogenase; *EC: 2.3.1.54=formate C-acetyltransferase; EC: 1.1.1.284=S-(hydroxymethyl)glutathione dehydrogenase; EC: 3.1.2.12=S-formylglutathione hydrolase; EC: 5.3.3.2=isopentenyl-diphosphate delta-isomerase; EC: 2.7.4.2=phosphomevalonate kinase; EC: 4.1.1.33=diphosphomevalonate decarboxylase; EC: 2.8.3.16=formyl-CoA transferase; EC: 2.8.3.5=3-oxoacid CoA-transferase; EC: 2.3.3.10=Hydroxymethylglutaryl-CoA synthase; EC: 1.1.1.174=3-Oxoadipyl-CoA thiolase; EC: 2.8.3.6=3-Oxoadipate CoA-transferase; EC: 6.3.2.3=glutathione synthase; *EC: 1.8.1.7=glutathione-disulfide reductase; EC: 4.1.3.1=isocitrate lyase; EC: 2.3.1.61=dihydrolipoyllysine-residue succinyltransferase; EC: 4.2.1.18=Methylglutaconyl-CoA hydratas; EC: 6.4.1.4=methylcrotonoyl-CoA carboxylase; EC: 1.3.8.4=isovaleryl-CoA dehydrogenase; EC: 1.2.4.4=3-methyl-2-oxobutanoate dehydrogenase (2-methylpropanoyl-transferring); *EC: 1.1.1.88=Hydroxymethylglutaryl-CoA reductase; EC: 1.2.1.27=methylmalonate-semialdehyde dehydrogenase (CoA acylating); EC: 2.7.1.36=mevalonate kinase; EC: 2.1.1.64=3-demethylubiquinol 3-O-methyltransferase; EC: 7.1.1.8=quinol--cytochrome-c reductase. The red asterisk (*) symbol functions as a crucial visual marker, highlighting specific KEGG enzymes that have been either substantiated through scientific inquiry or are hypothesized to potentially play pivotal roles in the onset or progression of Alzheimer’s disease (AD). The red dashed arrows denote distal biochemical processes or metabolites within the metabolic network. Additional details can be found in Figures S5, S6, S15, S18, S20, S21, S24, S28, S30, S32, S36, S37, S39, S46, S50, S54, S55, S57, S58, S60, S62, S64, and S66 and Tables S10-S12.

Further evaluation of the KEGG-mapped enzymes identified a subset of 33 enzymes, organized within 25 interconnected KEGG pathways, that showed consistent behavior and participate in the biosynthesis of metabolites with putative links to the initiation or progression of Alzheimer’s disease (AD) (Figures 9, S5, S6, S15, S18, S20, S21, S24, S28, S30, S32, S36, S37, S39, S46, S50, S54, S55, S57, S58, S60, S62, S64 and S66; Tables S10–S12). This functionally coherent enzyme subset offers a focused framework for probing how microbiome-derived metabolic activities may intersect with AD pathogenesis. Within this network, the core intermediate metabolites 3-methyl-3-glutaconyl-CoA, (S)-3-hydroxy-3-methylglutaryl-CoA, and glutamate appear as key nodes, potentially modulating multiple downstream routes relevant to AD-related neurobiology. The same analysis highlighted several terminal metabolites of particular interest, including anthranilate, glutathione (GSH), succinate, acetyl-CoA, (S)-malate, formate, lactate, acetate, and monoterpenoids, each mapped to specific supporting figures in the supplementary material (e.g., anthranilate, Figure S32; GSH, Figure S38; succinate and (S)-malate, Figure S54; acetyl-CoA, Figures S5, S50, S54, S55, S58, S60; formate and lactate, Figure S50; acetate, Figures S50, S58, S60; monoterpenoids, Figure S64). These end products are inferred to occupy potentially pivotal positions in AD-relevant pathways, and their altered biosynthetic potential in the AD oral microbiome justifies more detailed mechanistic studies on their roles in neurodegeneration.

Multiple additional metabolites were inferred to aggravate Alzheimer’s disease (AD) pathology when their levels were reduced in the oral microbiome of APP/PS1 mice. This group includes components of the oxidative phosphorylation machinery – cytochrome bc1 complex, 3-demethylubiquinone-n, and 3-demethylubiquinol-n, which are essential for electron transport and ATP generation (Figures S12 and S14). Further metabolites showing potentially deleterious associations with AD when diminished comprise estrone, allantoin, betaine, the inner core polysaccharide moiety of lipopolysaccharide (LPS), and amyloid-beta (Aβ) (Figures S13, S16, S19, S42 and S71). Collectively, these observations highlight nuanced metabolic disturbances within the oral microbiome that may influence oxidative balance, hormone and purine metabolism, inflammatory signaling, and amyloid dynamics, thereby underscoring the possible contribution of these molecular entities to AD onset and progression.

Table S14 indicates that PICRUSt-derived functional projections for the oral microbiomes of wild-type and APP/PS1 transgenic male mice are underpinned by remarkably low weighted NSTI scores, denoting excellent representation of community members by sequenced reference genomes and, therefore, a high expected accuracy of the inferred functional repertoire. Across both experimental cohorts, NSTI values are predominantly concentrated in the 0.01–0.04 range, with only a small subset of WT samples exhibiting modestly elevated scores (roughly 0.07–0.11), signifying that the bulk of taxa possess closely related genomic proxies and that PICRUSt-based functional profiles can be interpreted with substantial confidence. APP/PS1 mice do not display systematically increased NSTI values relative to WT animals; instead, both genotypes are characterized by similarly low indices overall, suggesting that any functional distinctions inferred between groups are unlikely to be artefacts of differential prediction quality.

## Discussion

In this *in silico* re-analysis of APP/PS1 and wild-type mice, our findings are consistent with the hypothesis that oral dysbiosis, selective gut shifts, and cross-niche enrichment in APP/PS1 animals coincide with a predicted reduction in microbiome-linked biosynthetic capacity for several candidate neuroprotective metabolites. These results are best interpreted as indicating a potential association between cross-niche microbial restructuring and diminished predicted production of metabolites involved in antioxidant defense, energy metabolism, and one-carbon pathways, providing a starting point for future work that applies higher-resolution sequencing and direct metagenomic and metabolomic profiling to test and refine the proposed mechanisms.

### Computationally inferred dysbiosis-associated diversity loss in AD pathogenesis

In the present study, we found that the APP/PS1 mice exhibit a stronger community-level alteration in the oral microbiome than in the gut microbiome. Specifically, oral beta diversity showed significant inter-cohort separation, whereas gut beta diversity did not differ significantly by ANOSIM, indicating that the gut microbiome was not globally reconfigured at the level of weighted UniFrac community structure. Nevertheless, the gut dataset still showed significant alpha-diversity differences and selective taxonomic shifts, including cross-niche enrichment of oral-associated genera such as *Fusobacterium* and *Streptococcus*. Accordingly, the present findings are most appropriately interpreted as evidence for oral dysbiosis accompanied by selective gut compositional alterations and putative ectopic enrichment patterns, rather than a wholesale dual-niche reorganization. Within this more conservative framework, these microbiome alterations were associated with a predicted reduction in the biosynthetic capacity of multiple neuroprotective metabolites.

Computational diversity metrics in the study indicated that the AD-associated gut microbiome exhibited predicted attenuation in diversity profiles, whereas the WT cohort demonstrated computationally inferred enrichment of microbial richness in their gut communities relative to their oral counterparts. These computational observations are consistent with findings from prior empirical and computational studies [7], 8], 10], 11], 39], 40] collectively suggesting that reductions in gut microbial diversity may represent a feature accompanying or reflecting neurodegenerative progression [41], 42]. Importantly, the absence of a significant ANOSIM result for the gut microbiome should not be interpreted as evidence that no gut alterations are present. ANOSIM evaluates overall multivariate community separation and therefore does not exclude statistically meaningful differences in alpha diversity or in the abundance of particular taxa between cohorts. In the present dataset, the non-significant gut beta-diversity result indicates that APP/PS1 and WT gut communities were not clearly separated at the whole-community level, but this does not negate the potential biological relevance of specific taxonomic shifts or cross-niche enrichment patterns detected within that overall structure.

The present *in silico* analysis suggests that oral microbiome alteration and selective gut microbiome changes may both be relevant to AD pathogenesis through interconnected yet mechanistically distinct pathways supported by prior empirical literature. According to established models, the altered oral microbiome may theoretically influence the central nervous system via cranial nerve signaling or microbial translocation, potentially heightening the risk of neuroinflammatory activation [43]. The circulating molecules, including lipopolysaccharides (LPS) and amyloid-beta (Aβ), have been reported to potentially exacerbate neuroinflammatory processes and are frequently identified co-localized in amyloid plaques within brain tissue [44], 45]. Oral-derived dysbiotic disturbances may theoretically compromise the blood–brain barrier (BBB), thereby facilitating the passage of inflammatory mediators and microbial components into brain parenchyma and amplifying neurodegenerative cascades, as supported by empirical evidence [5], 43], 46]. This computational characterization of oral dysbiosis together with selective gut alterations, combined with predicted metabolic disruption, suggests that maintaining microbial homeostasis across oral and gut ecosystems may be relevant to cognitive health [6], 47].

### Dominant microbial phyla and cross-niche interactions in AD

Computational analysis of 16S rRNA sequencing data revealed taxonomic patterns indicating that the dominant phyla in both microbiome types are Bacteroidota, followed by Bacillota and Pseudomonadota across wild-type (WT) and APP/PS1 transgenic cohorts. *In silico* characterization demonstrated that Bacillota and Pseudomonadota showed elevated predicted relative abundance in WT cohort microbiomes, whereas Bacteroidota exhibited increased predicted abundance in Alzheimer’s disease (AD) cohort microbiomes across both oral and gut microbial ecosystems, findings consistent with recent computational and empirical reports [9], 48].

Computational analysis of comparative microbial composition indicated putative patterns consistent with oral-to-gut translocation of *Fusobacterium* and *Streptococcus* species (Figures 5 and 7; Tables S7 and S9), taxa previously associated through empirical studies with neurodegeneration and cognitive decline in Alzheimer’s disease (AD) models [49]. Although the gut ANOSIM result was not significant, this does not preclude meaningful between-cohort differences in the abundance of particular taxa, because taxon-specific enrichment can occur without producing a statistically significant shift in overall multivariate community structure. Mechanistic literature indicates this translocation process may be linked to elevated lipopolysaccharide (LPS) levels and systemic inflammation. Within the oral niche, *Streptococcus* has been demonstrated to support *Fusobacterium nucleatum* colonization through adhesin–receptor interactions [50], 51], whereas in the gut environment, *Fusobacterium* is reported to disrupt microbial homeostasis and promote *Streptococcus* expansion [49], 52]. Our computational findings indicate this predicted imbalance correlates with increased *Streptococcus* abundance in AD microbiomes (Figure 4), which empirical evidence suggests may intensify intestinal inflammation and modulate neuroinflammatory signaling [7], 11], 53]. These findings illustrate computationally predicted interconnected microbial dynamics between oral and gut ecosystems that, according to prior literature, may influence host physiology. Conversely, the proposed migration of gut-associated bacteria, particularly *Muribaculum* and *Paramuribaculum*, to the oral cavity in AD mice may result from mechanisms including weakened immune function, barrier dysfunction, or immune cell-mediated bacterial transport, as supported by empirical evidence [54], 55]. Increased computational detection of gut-originating taxa in elderly oral microbiomes has been associated with age-related barrier alterations, suggesting that dysbiosis-associated selective ecological shifts may reflect secondary consequences of impaired mucosal immunity [46], 54].

### Metabolic network analysis and key metabolites

*In silico* analysis identified 85 enzymes with computationally predicted reduced abundance in Alzheimer’s disease (AD) mice (Figures S5–S71; Table S12). Among these dysregulated enzymes, 34 were computationally assigned to 25 intersecting KEGG metabolic pathways, forming a complex predicted metabolic network potentially consistent with AD pathophysiological mechanisms (Figure 9). It is important to emphasize that these PICRUSt-based functional profiles are *in silico* predictions inferred from 16S rRNA data rather than direct metagenomic measurements. Because PICRUSt relies on phylogenetic placement against annotated reference genomes, prediction accuracy is modulated by reference genome coverage (e.g., Nearest Sequenced Taxon Index, NSTI), and tends to decline as taxa become more phylogenetically distant from sequenced representatives. In the context of non-human mammalian microbiomes such as the APP/PS1 mouse model, the inferred enzyme and pathway abundances should therefore be regarded as approximate and hypothesis-generating; confirmation will require strain-resolved metagenomics and targeted metabolomic validation [32], 33], 38]. It is important to note that these predictions pertain specifically to microbial enzymatic capacity inferred from 16S rRNA profiles, and do not capture the substantial contribution of host metabolic pathways to the biosynthesis and regulation of these metabolites. In other words, the present analysis suggests a decline in the microbiome-associated synthetic potential for selected neuroprotective metabolites, rather than demonstrating an absolute systemic depletion of these compounds in host tissues. In addition, the present analysis is based on an OTU-clustered 16S rRNA pipeline and PICRUSt1 rather than on ASV-resolved workflows and PICRUSt2, which are now more widely adopted for microbiome functional inference [33], 56]. This reliance on a legacy processing framework should be understood as a constraint of the original dataset and re-analysis pipeline, and it further reinforces that our findings are intended to generate testable hypotheses regarding putative neuroprotective metabolite insufficiency, rather than to provide definitive causal mechanisms.

Computational assignment and pathway mapping identified metabolites most likely to relate to disease-linked metabolic alterations inferred from enzymatic reductions, including glutathione (GSH) (Figure S38), acetyl-CoA (Figures S5, S50 and S54–S60), succinate, (S)-malate (Figure S54), formate (Figure S50), lactate (Figures S5 and S50), acetate (Figures S50, S58 and S60), anthranilate (Figure S32), cytochrome bc1 complex III, and monoterpenoids (Figure S64). These computationally integrated molecules are predicted to indicate altered biochemical routes potentially impacting redox equilibrium, energy metabolism, and neurotransmitter synthesis, though experimental metabolomic validation is required to confirm whether predicted enzymatic reductions translate to actual metabolite insufficiencies.

#### Antioxidant metabolism: glutathione and redox balance

Glutathione (GSH) serves as the brain’s most prominent endogenous antioxidant, functioning to maintain cellular redox balance and counteract oxidative injury through multiple enzymatic pathways [57]. This analysis predicted reduced biosynthetic capacity for GSH in AD-associated dysbiosis based on enzymatic reductions (Figure S38). Empirical literature demonstrates that GSH depletion has been associated with memory loss, oxidative stress, and neuroinflammatory processes contributing to AD pathology [58], [59], [60], [61], with post-mortem studies confirming reduced GSH levels in multiple brain regions of AD patients [60]. Notably, experimental supplementation with γ-glutamylcysteine (a GSH precursor) has been demonstrated to restore redox capacity, attenuate amyloid pathology, and improve cognitive function in preclinical models [58], 62]. Within this framework, the current results should be interpreted as indicating a potential reduction in microbial pathways that could support or modulate glutathione availability, rather than as direct evidence that total host glutathione pools are depleted in APP/PS1 mice. Host cells retain intrinsic capacity to synthesize GSH, and only integrated metabolomic and functional studies can establish whether microbiome-level changes materially influence systemic redox status.

#### Energy metabolism: acetyl-CoA, succinate, and malate

Acetyl-CoA supports critical neuronal functions including energy metabolism and acetylcholine synthesis, while also mediating epigenetic regulation through histone acetylation [63], [64], [65]. Literature indicates that reduced acetyl-CoA availability can impair mitochondrial function and cholinergic signaling in AD [63]. The analysis identified that although pyruvate dehydrogenase activity (EC 2.3.1.12) was not computationally predicted to be reduced, a related enzyme – formate C-acetyltransferase (EC 2.3.1.54) – showed predicted decreased abundance in the oral microbiome of APP/PS1 mice (Figure 9; Table S10). This enzyme participates computationally in an alternative biosynthetic route for acetyl-CoA from pyruvate (Figures S50 and S57). These computational findings align with empirical literature proposing acetyl-CoA’s role in supporting mitochondrial stability and neuronal survival [63], suggesting that dysbiosis-associated reduction in predicted acetyl-CoA biosynthetic capacity may represent a mechanistic contributor to AD pathology.

Succinate and malate serve as pivotal central intermediates within the citric acid cycle, where their catalytic participation is essential for optimal adenosine triphosphate (ATP) synthesis and maintenance of cellular energetic and metabolic homeostasis [66], 67]. Empirical studies demonstrate that reductions in succinate levels in AD neurons may establish a metabolic bottleneck leading to energetic deficiency, cognitive decline, and neuronal loss [67], [68], [69], [70]. Literature evidence indicates that succinate imbalance may trigger pro-inflammatory signaling [71] and interact with amyloid-beta (Aβ)-induced oxidative stress pathways [72], 73], with emerging therapeutic approaches exploring metabolic pathway manipulation including succinate restoration as potential AD interventions [68], 74]. Our computational analysis predicted reduced abundance of enzymes supporting succinate synthesis (Figure S54), suggesting dysbiosis-associated impairment of this critical metabolic node. Malate depletion may similarly compromise redox cycling and mitochondrial efficiency according to empirical evidence [75], 76], and given malate’s role in the malate-aspartate shuttle (MAS), predicted reductions in malate biosynthesis could theoretically disrupt NADH transport and oxidative phosphorylation [77]. Experimental metabolomic validation is required to confirm whether computationally predicted reductions in acetyl-CoA, succinate, and malate biosynthetic capacity translate to functional bioenergetic insufficiency in dysbiosis-associated AD. Thus, the predicted attenuation of microbial pathways contributing to acetyl-CoA, succinate, and malate synthesis should be viewed as a potential modifier of host bioenergetics rather than a stand-alone determinant. The host’s own metabolic machinery can generate these intermediates, and future work will need to disentangle how microbial and host sources interact under dysbiotic conditions to shape neuronal energy metabolism.

#### One-carbon metabolism and neurotransmitter support: formate

Reduced microbial formate generation in AD has been empirically associated with altered one-carbon metabolism and mitochondrial dysfunction according to prior studies [78], 79]. Formate contributes to critical biosynthetic pathways including nucleotide and amino acid synthesis; consequently, its depletion may broaden metabolic imbalance through effects on homocysteine cycling and related methylation pathways [80], 81]. The analysis identified predicted reduced abundance of formate C-acetyltransferase (EC 2.3.1.54), a key enzyme in formate-dependent acetyl-CoA biosynthesis (Figure S50), suggesting dysbiosis-associated impairment of this metabolic route. The predicted integrity loss of this computational pathway may hold implications for bioenergetic regulation and neuroprotection, suggesting formate restoration through dysbiosis correction or direct supplementation warrants future experimental exploration in AD therapeutic development [82], [83], [84]. Again, our data imply a shift in the microbiome’s inferred contribution to formate-linked one-carbon metabolism, not a direct measurement of whole-organism one-carbon flux, which remains heavily determined by host enzymatic pathways and dietary inputs.

#### Short-chain fatty acids: lactate and acetate

Lactate serves as a critical metabolic substrate supporting neuronal energy metabolism through the astrocyte–neuron lactate shuttle (ANLS), wherein astrocytic lactate production provides energetic support during periods of high neuronal activity [85]. Empirical evidence indicates that lactate transport dysfunction in AD, manifested by decreased expression of monocarboxylate transporters MCT1, MCT2, and MCT4, may exacerbate energetic deficits and contribute to neuronal dysfunction [86], 87]. Acetate represents one of the most abundant short-chain fatty acids (SCFAs) produced by gut microbiota, exhibiting multifaceted physiological effects on host metabolism and neuroinflammation [88]. Experimental evidence demonstrates that acetate administration can reduce microglial activation and enhance cognitive performance in APP/PS1 mice [89], suggesting neuroprotective potential. However, literature also indicates that excessive acetate levels or co-stimulation with pro-inflammatory factors may paradoxically promote inflammatory cytokine release [90], highlighting concentration- and context-dependent effects. This dual anti- and pro-inflammatory capacity, regulated by both acetate concentration and the local inflammatory milieu, emphasizes the nuanced and complex role of SCFAs in modulating neuroinflammatory responses [90], 91]. This computational analysis predicted reduced abundance of enzymes supporting acetate biosynthesis (Figures S50, S58, S60), suggesting dysbiosis-associated SCFA insufficiency may compromise neuroprotective signaling, though optimal therapeutic acetate concentrations require careful titration to avoid pro-inflammatory effects. Accordingly, any dysbiosis-associated reduction in inferred microbial lactate and acetate pathways should be regarded as a putative perturbation of microbially derived SCFA support, rather than definitive proof that systemic SCFA pools are globally diminished in the host.

#### Monoterpene biosynthesis: neuroprotective phytochemical potential

Computational analysis in this study identified six enzymes with computationally predicted reduced abundance mapped within the “Terpenoid backbone biosynthesis” and “Monoterpenoid biosynthesis” pathways (Figures 9 and S64), suggesting dysbiosis-associated impairment of monoterpenoid production capacity. Empirical literature establishes that monoterpenes possess significant antioxidant and anti-inflammatory capabilities, functioning through multiple mechanisms including neutralization of reactive oxygen species, enhancement of acetylcholine availability through acetylcholinesterase inhibition, and direct suppression of amyloid-beta (Aβ) production [92]. Monoterpenes have been experimentally demonstrated to target critical pro-inflammatory mediators including p38 MAPK, cyclooxygenase-2 (COX-2), and nitric oxide synthase-2 (NOS-2), thereby modulating neuroinflammatory signaling pathways implicated in AD pathogenesis [93], 94]. Additionally, monoterpenes exhibit modulatory effects on acetylcholinesterase and β-secretase (BACE1) enzymes, suggesting potential therapeutic applicability for addressing both cholinergic deficiency and amyloid pathology in AD [92], 95]. The computational prediction of reduced monoterpenoid biosynthetic capacity in dysbiotic oral microbiota suggests that microbial-derived monoterpenes may represent an underappreciated neuroprotective mechanism whose loss could contribute to AD vulnerability.

#### Oxidative phosphorylation support: ubiquinone and electron transport

Computational pathway mapping in Figure 9 indicated relatively higher predicted glutamate levels in WT mice compared to APP/PS1 mice. While glutamate serves as the primary excitatory neurotransmitter critical for synaptic signaling, empirical evidence establishes that excessive glutamate accumulation can induce excitotoxic neuronal damage through calcium overload and oxidative stress [96], 97]. The computationally observed elevation in WT mice may reflect functional metabolic balance wherein glutamate levels are counterbalanced by metabolites that modulate glutamate release or receptor sensitivity. Specifically, this *in silico* analysis identified anthranilate – a tryptophan metabolism product catalyzed by kynureninase (EC 3.7.1.3) and arylamidase (EC 3.5.1.9) (Figure S32) – and ubiquinone (coenzyme Q10), synthesized through terpenoid-quinone biosynthesis pathways (Figure S12), as possible modulators predicted to differ between cohorts.

Ubiquinone functions as an essential electron carrier within the mitochondrial electron transport chain, transferring electrons between Complex I/II and Complex III to sustain oxidative phosphorylation and ATP generation [98], 99]. Empirical studies demonstrate that impaired ATP production and excessive reactive oxygen species (ROS) formation – consequences of electron transport chain dysfunction – have been causally linked to synaptic dysfunction, dendritic spine loss, and progressive neurodegeneration in AD [100], [101], [102]. This computational prediction of reduced ubiquinone biosynthetic enzyme abundance in dysbiotic microbiota suggests potential compromise of mitochondrial bioenergetic capacity, whereas computationally predicted efficient energy metabolism in WT mice may enhance neuronal resilience to oxidative and energetic stress.

#### Additional metabolites potentially associated with alzheimer’s disease

*In silico* analysis computationally identified additional metabolites with predicted decreased inferred abundance in AD mice, including estrone (Figure S13), allantoin (Figure S16), betaine (Figure S19), the core polysaccharide component of lipopolysaccharide (LPS) (Figure S42), and amyloid-beta (Aβ) (Figure S71).

Estrone, a neuroprotective steroid hormone, has been established in empirical literature to influence neural pathways potentially related to AD susceptibility [103], though clinical findings on estrogen-based therapeutic interventions remain inconclusive [103], 104]. Allantoin, an antioxidant purine derivative, has been experimentally associated with mitigating amyloid-beta (Aβ)-induced toxicity and reducing Tau hyperphosphorylation through modulation of the PI3K/Akt/GSK-3β signaling pathway [105], 106]. Computational prediction of reduced allantoin biosynthetic capacity in dysbiotic microbiota suggests potential compromise of this antioxidant defense mechanism. Betaine, a trimethylglycine compound, supports cellular methylation balance and has been empirically linked to anti-inflammatory activity through suppression of NLRP3 inflammasome and NF-κB signaling pathways [107], 108]. Predicted dysbiosis-associated betaine insufficiency may compromise both methylation homeostasis and anti-inflammatory capacity. Amyloid-beta (Aβ) peptides represent central neuropathological features of AD [109], 110], particularly their soluble oligomeric forms, which empirical evidence demonstrates contribute to oxidative stress and neuronal dysfunction [111]. According to the amyloid cascade hypothesis, Aβ accumulation initiates a pathogenic cascade leading to neuroinflammation and metabolic enzyme dysregulation [110]. Lipopolysaccharide (LPS), a structural component of Gram-negative bacterial cell membranes, is well-established for its role in triggering neuroinflammatory signaling through toll-like receptor activation [112]. Notably, the analysis revealed a relative computational increase in LPS core polysaccharide abundance (Figure S42) coinciding with lower predicted Aβ levels (Figure S71) in wild-type mice compared to APP/PS1 mice – a counterintuitive pattern potentially reflecting enhanced Aβ clearance through LPS-induced microglial activation, as supported by empirical evidence [113], 114]. However, this interpretation requires careful consideration: variations in LPS and Aβ levels across different AD disease stages, species-specific immune responses, and the distinction between pro-inflammatory versus pro-clearance microglial phenotypes may influence these computational patterns [115], [116], [117].

In summary, the computational metabolic interactions identified through computational analysis reveal a network of interconnected antioxidant, bioenergetic, and neurotransmitter-related processes that are putatively altered in Alzheimer’s disease (AD). This pathway mapping predicts that key compounds including glutathione (GSH), acetyl-CoA, succinate, malate, formate, and short-chain fatty acids (SCFAs) may collectively influence cellular redox balance and neuronal energy metabolism when dysbiosis-associated enzymatic capacity is compromised. Additionally, computationally predicted alterations in metabolites such as anthranilate and ubiquinone may affect excitotoxic stress buffering and mitochondrial antioxidant defense, respectively.

Beyond this primary metabolic network, *in silico* analysis identified secondary metabolites with predicted reduced biosynthetic capacity including estrone (Figure S13), allantoin (Figure S16), betaine (Figure S19), the lipopolysaccharide (LPS) core polysaccharide (Figure S42), and amyloid-beta (Aβ) (Figure S71), suggesting potential dysbiosis-associated impacts on hormonal neuroprotection, purine-derived antioxidant defense, methylation homeostasis, microbial membrane structure, and amyloidogenic processing, respectively. The concentration of enzymatic dysregulation within functionally coherent metabolic pathways – encompassing both primary neuroprotective compounds and secondary regulatory metabolites – rather than random enzymatic loss, provides direction for future biochemical analyses through targeted metabolomic validation, mitochondrial functional assessment, examination of hormonal and methylation pathways, clarificati on of LPS core polysaccharide bioactivity, and mechanistic studies in dysbiosis-corrected animal models to establish whether these computationally predicted metabolic alterations reflect biological reality and contribute causally to cognitive decline.

Crucially, these *in silico* predictions pertain to the microbial arm of metabolism and suggest that the microbiome’s potential to supply or modulate neuroprotective metabolites may be diminished, but they do not demonstrate that total host-level metabolite concentrations fall below physiological requirements.

### Systems-level integration: bidirectional ectopic occurrence, metabolic dysregulation, and neurodegeneration

The computational overlap between cross-niche enrichment patterns and predicted reductions in enzymatic and metabolic capacity suggests a hypothesis-generating model in which selective microbial redistribution signals, rather than confirmed whole-community reorganization across both niches, may be associated with altered metabolic potential. *In silico* analysis indicates that bacterial taxa, when established in their native anatomical niches, maintain metabolic profiles theoretically optimized for local nutrient availability, oxygen tension, pH conditions, and host interactions [57], 63], 78], 98], 118]. These computational predictions suggest that detection of oral-associated pathogens (*Fusobacterium*, *Streptococcus*) within the gastrointestinal tract may displace their predicted oral-centric metabolic specialization, potentially reducing their inferred capacity to synthesize oral-centric metabolites while simultaneously disrupting the predicted metabolic output of gut-resident commensals through competition for shared substrates and altered microbe–microbe interactions. Similarly, reciprocal detection of gut-associated taxa (*Muribaculum*, *Paramuribaculum*) in the oral cavity may constrain their inferred metabolic potential and displace indigenous oral commensals with distinct enzymatic repertoires. These cross-niche enrichment patterns correspond computationally to the observed reduction of 85 oral-enriched enzymes in APP/PS1 mice – a numerical and potential functional loss that, according to our *in silico* predictions, may not be compensated by alternative metabolic routes or by the enzymatic repertoires of bacteria detected at ectopic sites.

It is important to emphasize that these *in silico* observations are based on 16S rRNA amplicon profiles and therefore capture patterns of cross-niche taxonomic enrichment rather than direct evidence of viable microbial translocation. The detection of typically oral-associated taxa (*Fusobacterium*, *Streptococcus*) within gut communities and reciprocal enrichment of gut-associated taxa (*Muribaculum*, *Paramuribaculum*) in the oral microbiome is thus interpreted as ectopic occurrence or cross-niche enrichment, not as definitive proof of bidirectional colonization. Alternative mechanisms, including sample cross-contamination during collection or processing, detection of non-viable microbial DNA disseminated via hematogenous or lymphatic routes, or parallel ecological reshaping of anatomically distinct niches under shared host factors, cannot be excluded on the basis of the present 16S rRNA dataset. Consequently, the present findings should be regarded as hypothesis-generating evidence that cross-niche enrichment patterns along the oral–gut axis may coincide with dysbiosis-linked neuroprotective metabolite insufficiency, while definitive demonstration of bidirectional colonization will require strain-resolved metagenomics, culture-based validation, and tracking of viable microorganisms in future studies.

### Hierarchical metabolic collapse and convergent pathway dysfunction

The observed dysbiosis initiates a possible hierarchical metabolic collapse wherein depletion of oral microbiome-specific enzymatic repertoires simultaneously impairs three functionally coupled metabolic networks essential for neuronal integrity [118], [119], [120]. Specifically, antioxidant biosynthetic pathways and electron transport systems become functionally dissociated from oxidative phosphorylation, thereby destabilizing downstream one-carbon metabolism and neurotransmitter precursor synthesis [57], 63], 78], 98]. This metabolic decoupling propagates through self-reinforcing feedback mechanisms: diminished ATP availability constrains the activity of energy-dependent antioxidant enzymes, escalating oxidative damage that subsequently suppresses transcriptional and post-translational regulation of metabolic enzyme expression [58], 100], 121], 122]. The resulting metabolic dysfunction represents a convergent pathobiological process wherein simultaneous deficiencies in bioenergetic capacity, redox homeostasis, and inflammatory regulation collectively manifest as progressive cognitive decline [41], 100], 119], 123]. This systems-level dysregulation represents a form of metabolic convergence wherein dysbiosis simultaneously impairs multiple independent pathways supporting a single critical function-neuronal energy production, antioxidant defense, and synaptic transmission. These findings collectively substantiate an integrated mechanistic model wherein spatial dysregulation of microbial communities disrupts ecosystem-specific metabolic functions, thereby diminishing the production of neurodefensive molecules and precipitating cognitive dysfunction via compounded deficiencies in cellular energy metabolism, oxidative balance, and inflammatory homeostasis.

### Methodological limitations of predictive functional profiling

A central methodological limitation of this work is the reliance on predictive functional profiling rather than direct functional measurement. PICRUSt infers enzyme (EC) abundances and KEGG pathway content from 16S rRNA taxonomic profiles by reference-based imputation, and these *in silico* predictions cannot be considered equivalent to shotgun metagenomic sequencing or experimental metabolomics. The accuracy of these predictions is constrained by the completeness and phylogenetic breadth of the reference genome database, the Nearest Sequenced Taxon Index of community members, and the assumption that closely related taxa share similar functional repertoires. Consequently, the inferred enzyme abundances and pathway activities are subject to both false-positive and false-negative biases and should be interpreted as approximate indicators of potential functional capacity rather than as quantitative measures of realized metabolism. In this context, the enzyme and metabolite patterns reported here are best viewed as hypothesis-generating leads that require validation in future studies using strain-resolved metagenomics and targeted metabolomic or fluxomic analyses, particularly in non-human AD models such as APP/PS1 mice.

A further limitation is that the present analysis does not distinguish between microbial and host contributions to the metabolite pools of interest. Our PICRUSt-based pipeline infers enzyme and pathway abundances from microbial 16S rRNA profiles and therefore quantifies only a microbiome-associated synthetic potential. Many of the highlighted metabolites (e.g., glutathione, acetyl-CoA, succinate, lactate, monoterpenoids) can also be synthesized *de novo* by host tissues or derived from diet. The current results should therefore not be interpreted as evidence of absolute systemic deficiency, but rather as a hypothesis that diminished microbial biosynthetic capacity may interact with host metabolism in ways that favor neurodegenerative progression. Confirming this will require integrated host–microbiome metabolomics and functional studies.

An additional limitation is that the gut microbiome did not show significant inter-cohort separation by ANOSIM based on weighted UniFrac distances. Therefore, the present data do not support a conclusion of global gut community restructuring in APP/PS1 mice. Instead, gut-related interpretations should be confined to significant richness differences, selective taxonomic variation, and predicted functional changes that warrant further validation. Taken together, these considerations underscore that the present work should be viewed as a hypothesis-generating *in silico* analysis constrained by legacy 16S and predictive functional pipelines, with extensive metabolic interpretation that is intentionally framed as tentative and subject to revision as higher-resolution sequencing and direct metagenomic/metabolomic validation become available.

### Additional study constraints and future directions

While this investigation provides computationally integrated evidence for dysbiosis-associated metabolic insufficiency in Alzheimer’s disease, several constraints merit acknowledgment. The cross-sectional design precludes temporal precedence determination between dysbiosis and cognitive decline. Moreover, the proposed mechanisms of ectopic occurrence along the oral–gut axis remain provisional; causal attribution will require germ-free models, antimicrobial perturbation, and transplantation experiments. The analysis circumscribes investigation to oral and enteric microbiota, omitting accessory mucosal compartments. In addition, no direct empirical linkage is established here between dysbiosis and behavioral phenotypes; mechanistic validation will require combined behavioral assessment, neuropathological quantification, and multi-omics integration. Conversely, this investigation uniquely characterizes dual mucosal niches simultaneously, enabling direct detection of reciprocal cross-niche enrichment patterns. The integration of phylogenetic composition with predictive metabolomic networks establishes mechanistic hypotheses linking discrete bacterial taxa to neuroprotective molecules (glutathione, acetyl-CoA, succinate, formate, short-chain fatty acids), furnishing defined therapeutic targets. The identification of 85 dysregulated enzymes within 25 interconnected pathways prioritizes experimental validation. This systems-level model reconceptualizes dysbiosis as a spatial redistribution of microbial communities, proposing novel compartmentalization-targeted interventions. By explicitly framing predictions as hypothesis-generating, this study establishes a conceptual scaffold for subsequent investigation of the oral–gut–brain axis in Alzheimer’s disease pathogenesis.

## Conclusions

This *in silico* analysis supports a predicted unified dysbiosis–metabolic insufficiency model in Alzheimer’s disease, in which altered oral–gut microbial traffic disrupts neuroprotective metabolite synthesis along the oral–gut–brain axis. Predicted loss of 85 orally enriched enzymes across 25 KEGG pathways suggests linked disturbances in antioxidant defenses, bioenergetic metabolism, and one-carbon pathways. Together, these shifts may contribute to cognitive decline through interacting bioenergetic, redox, and inflammatory vulnerabilities. The framework points to possible stage-adapted interventions, ranging from microbiome-focused strategies to metabolite support and barrier/anti-inflammatory approaches at more advanced stages. Nonetheless, comprehensive validation using metabolomics, cognitive rescue studies, and germ-free models will be important before considering clinical application.

## Supplementary Material

Supplementary Material

Supplementary Material

Supplementary Material

Supplementary Material

Supplementary Material

Supplementary Material

Supplementary Material

Supplementary Material

Supplementary Material

Supplementary Material

Supplementary Material

Supplementary Material

Supplementary Material

Supplementary Material

Supplementary Material
