# Supplementary material for: In silico dysbiosis-associated neuroprotective metabolite insufficiency in Alzheimer’s disease
Source: Open Med (Wars). 2026 Jul 25;21(1):20261484. doi: 10.1515/med-2026-1484 (PMC13398440; doi:10.1515/med-2026-1484)
Supplement: Supplementary file 1 — Supplementary Material [file j_med-2026-1484_suppl_001.docx]

Fig. S1. Specaccum or species accumulation curve of the oral (a) and gut (b) microbiome samples of wild type male mice (WT) and age-matched transgenic male littermates expressing amyloid precursor protein/presenilin-1 (APP/PS1).


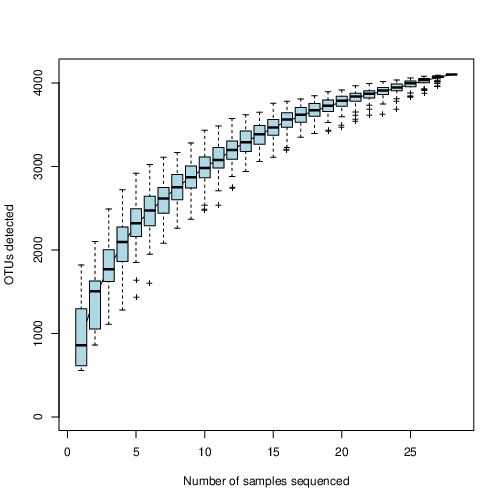

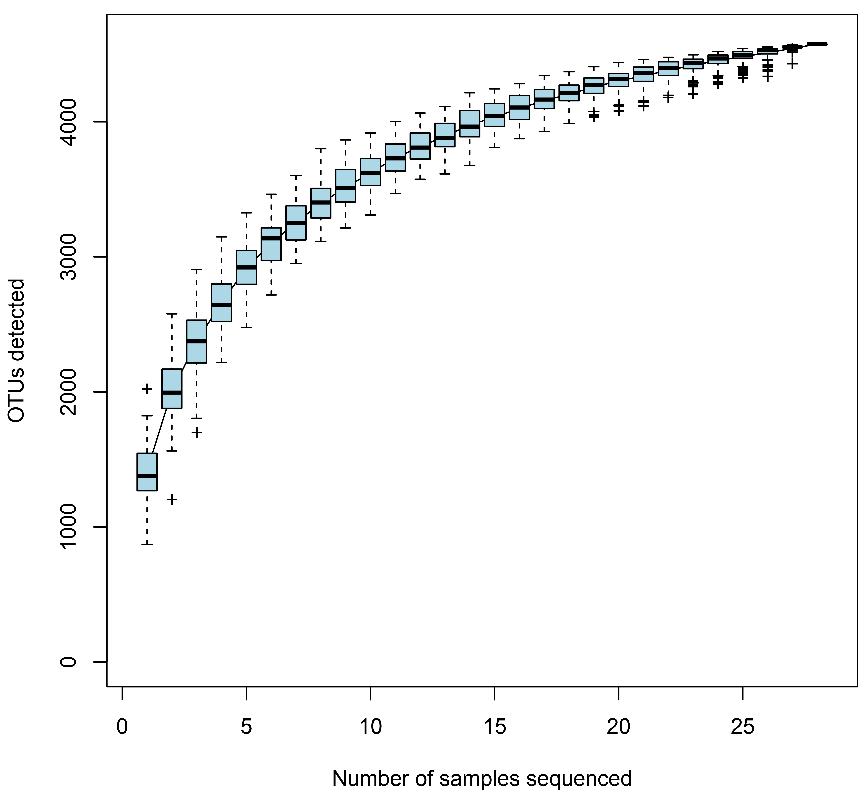


**(a)**

**(b)**

Fig. S2. Anosim plot of oral (a) and gut (b) microbiomes of wild type male mice (WT) and age-matched transgenic male littermates expressing amyloid precursor protein/presenilin-1 (APP/PS1).


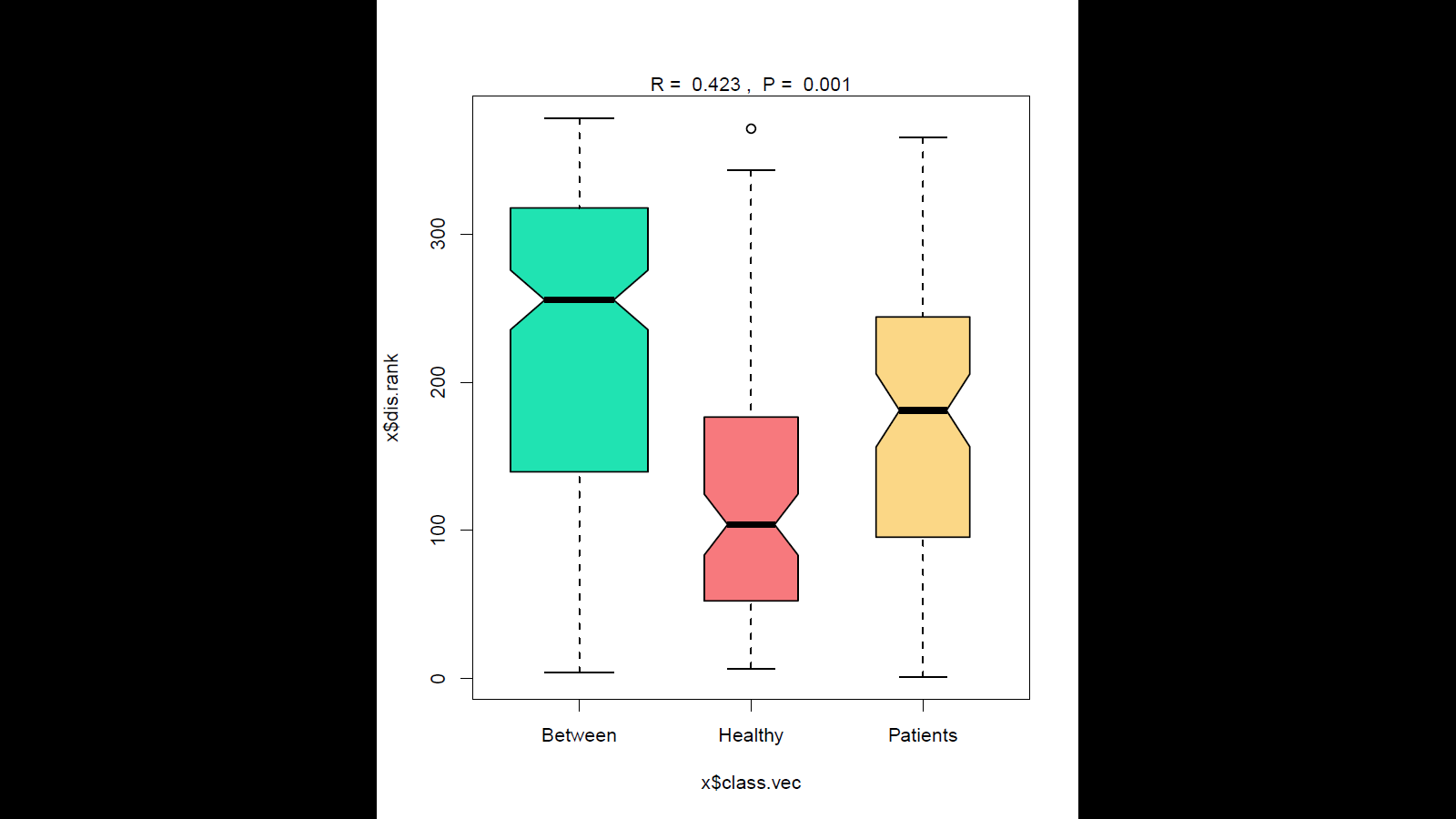


**Between**

**APP/PS1**

**R = 0.423, P = 0.001**

**WT**

**Rank**

**(a)**


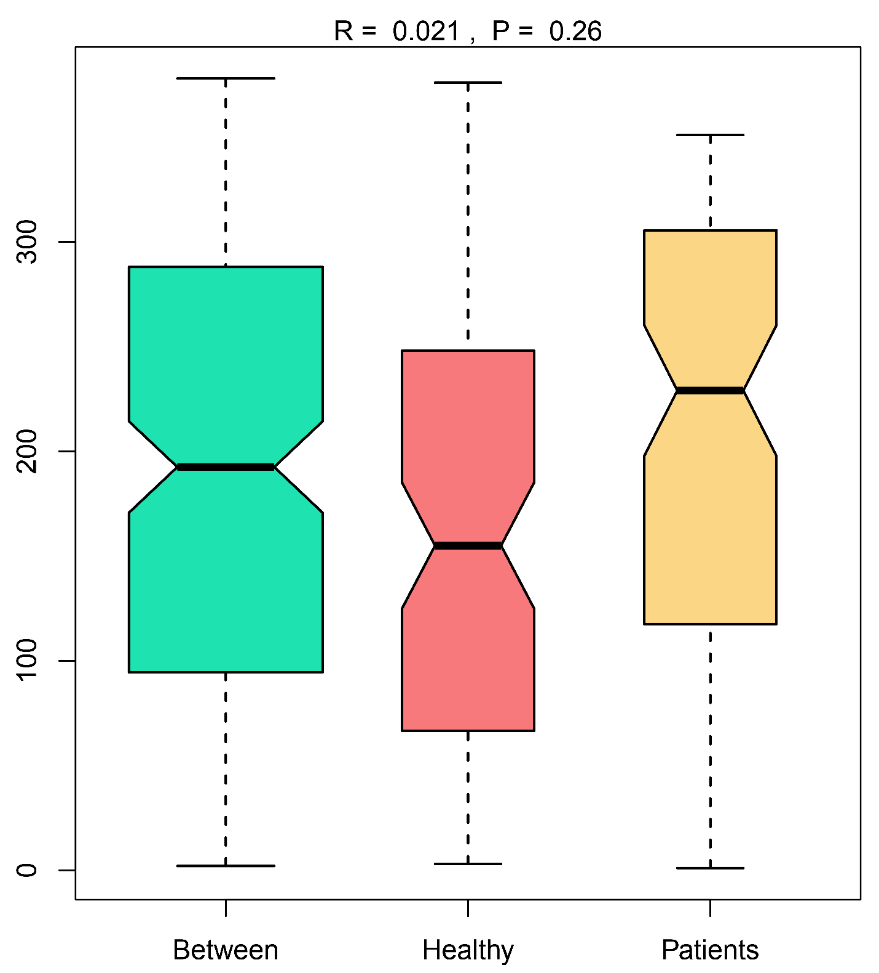


**WT**

**APP/PS1**

**Between**

**R = 0.021, P = 0.26**

**Rank**

**(b)**

Fig. S3. Boxplots of observed species (Sobs), alongside the “Chao1” and “ACE” alpha diversity indices, characterizing the oral (a) and gut (b) microbiomes of wild type male mice (WT) and age-correlated transgenic male littermates expressing amyloid precursor protein/presenilin-1 (APP/PS1). Additional information is available in Table S2.


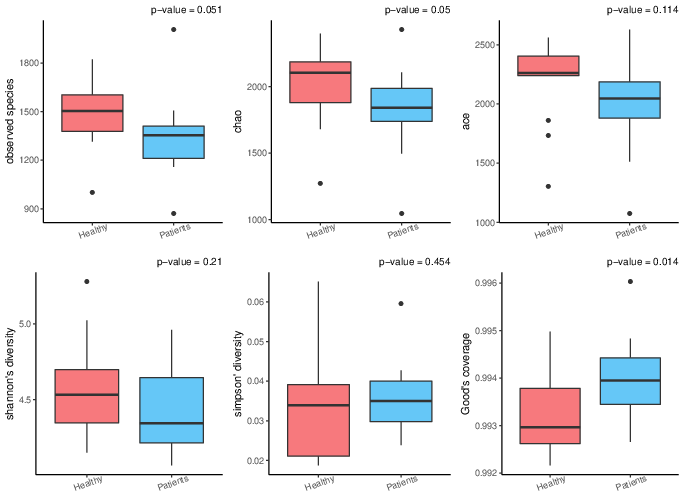


**P value = 0.051**

**P value = 0.114**

**P value = 0.005**

**Observed species**

**Chao1**

**ACE**

**WT**

**WT**

**WT**

**APP/PS1**

**APP/PS1**

**APP/PS1**

**(b)**


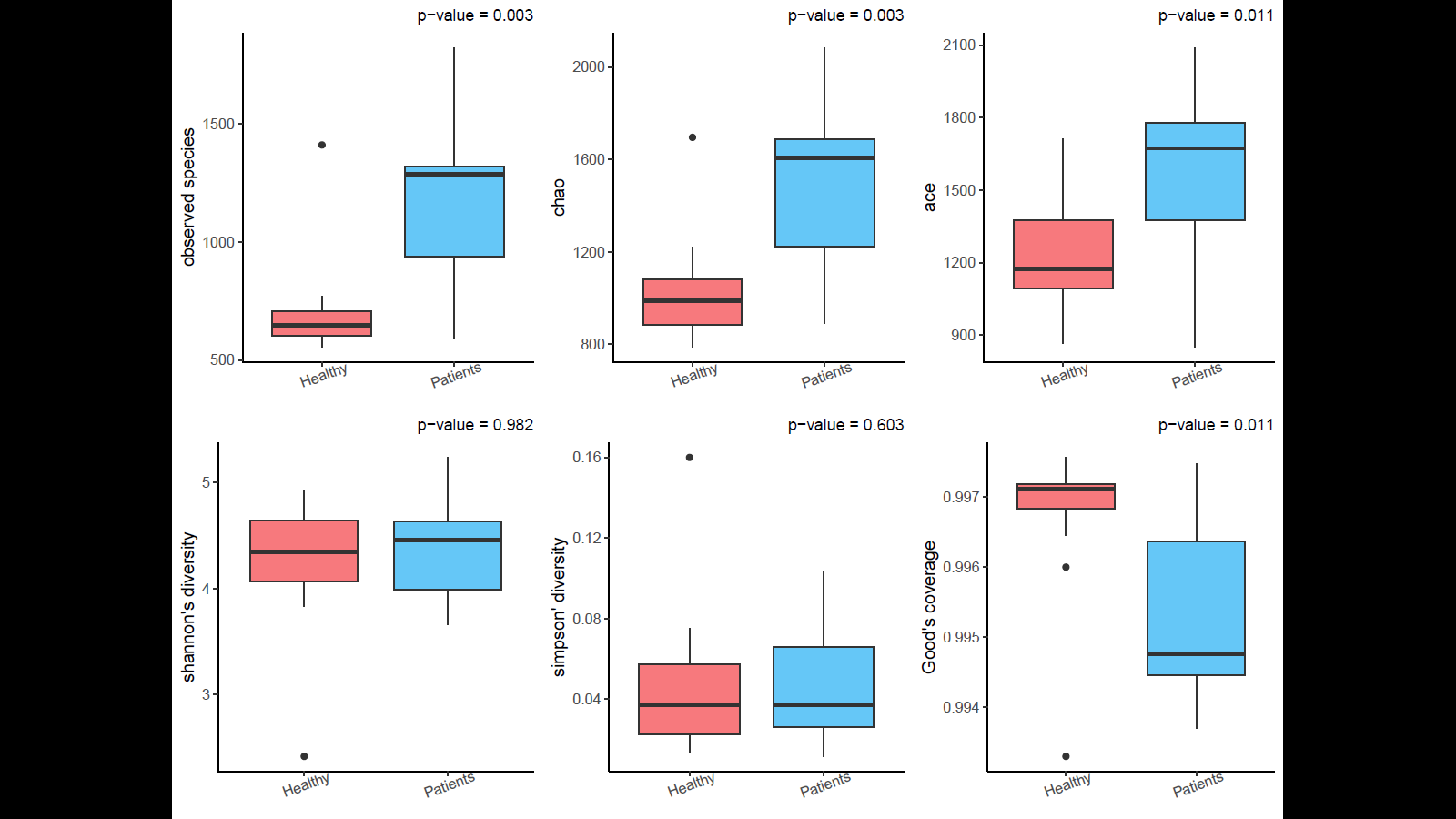


**P value = 0.003**

**P value = 0.011**

**P value = 0.003**

**Observed species**

**Chao1**

**ACE**

**WT**

**WT**

**WT**

**APP/PS1**

**APP/PS1**

**APP/PS1**

**(a)**

Fig. S4. Number of observed species as a rarefaction metric, delineating the least sequencing depth necessary to encompass all samples within the oral (a) and gut (b) microbiome datasets, facilitating the assessment of taxonomic relative abundance as a metric of beta diversity across the gut microbiomes of wild type male mice (WT) and age-matched transgenic male littermates expressing amyloid precursor protein/presenilin-1 (APP/PS1).


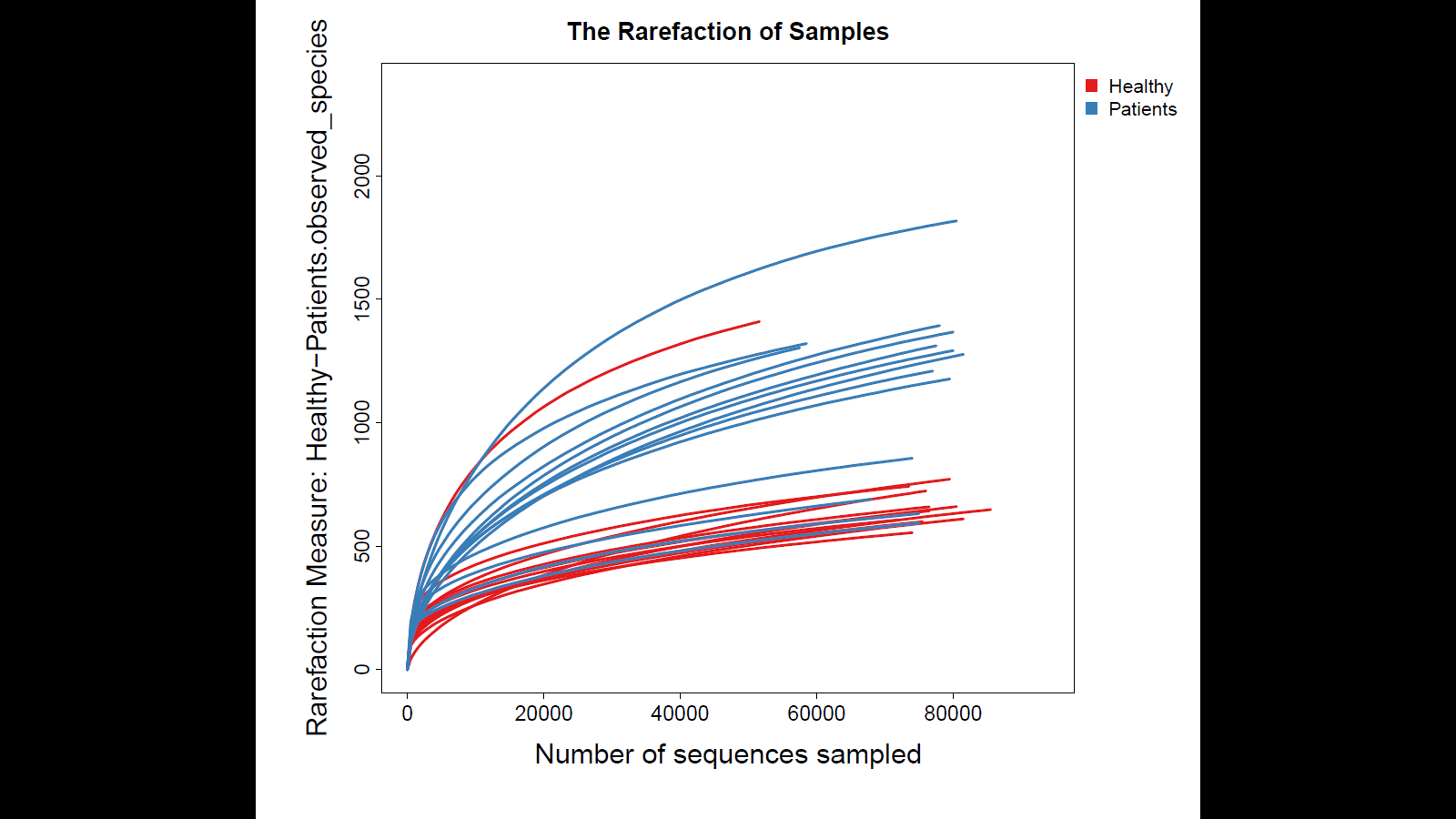


**51600**

**Number of sequences sampled**

**The rarefaction of samples**


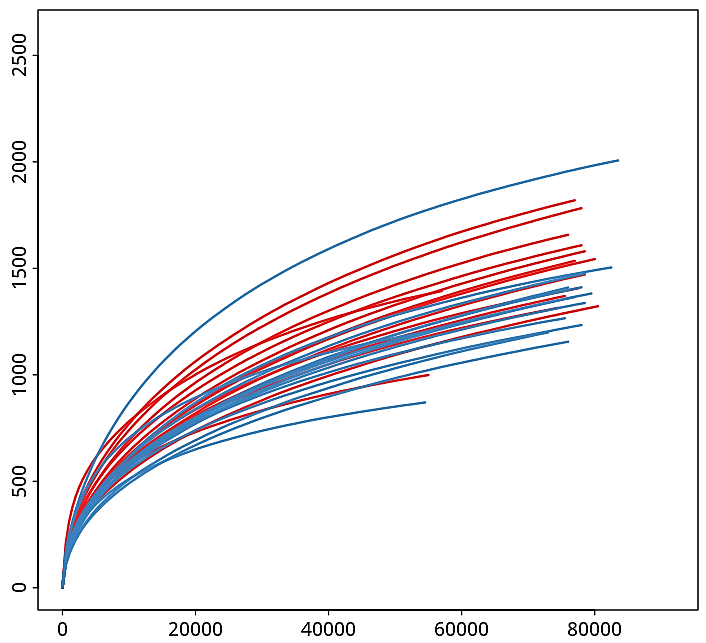


**55000**

**WT**

**APP/PS1**

**WT**

**APP/PS1**

**Number of OTUs**

**Number of OTUs**

**(a)**

**(b)**


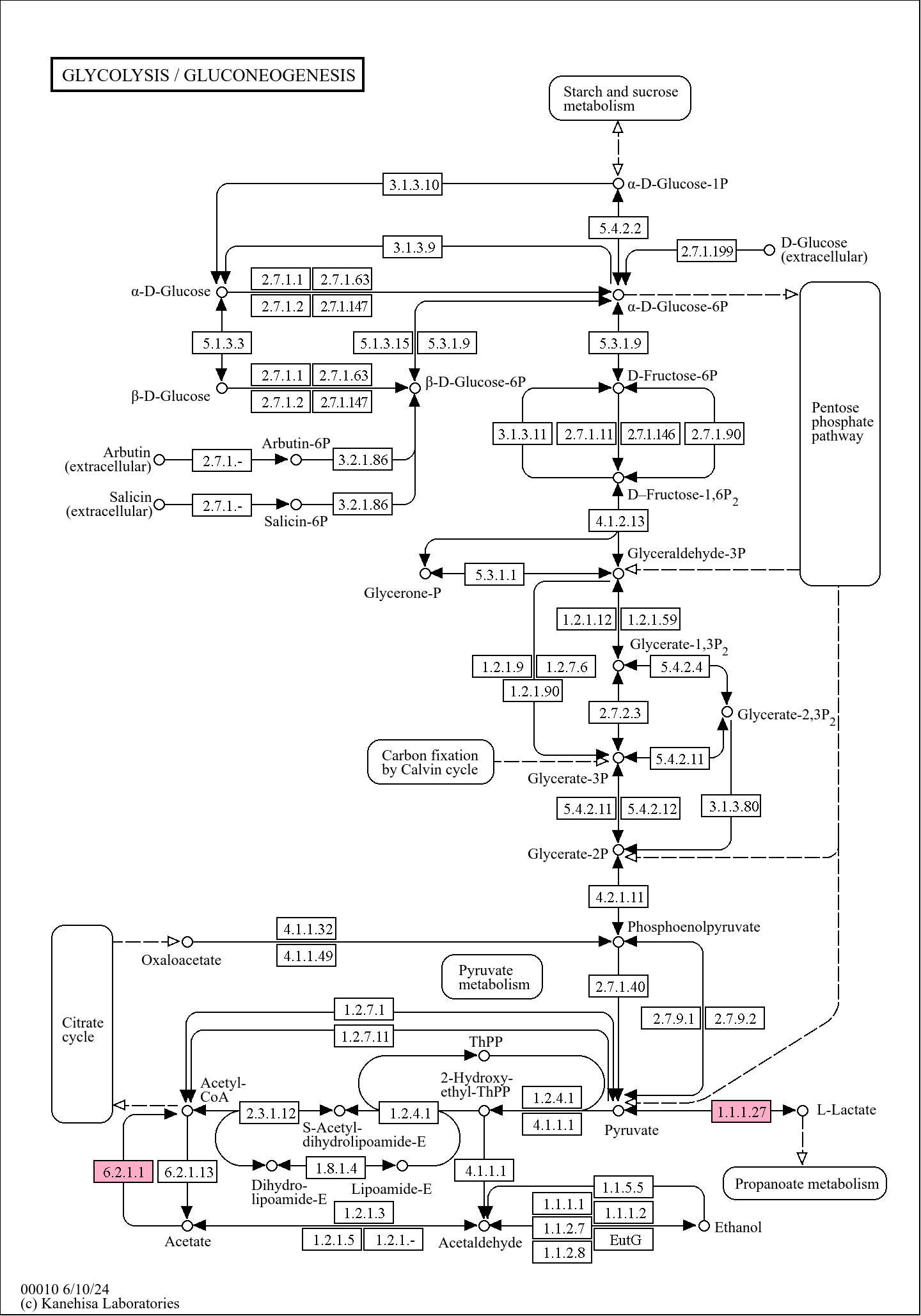
Fig. S5. Enzymatic profile of the "Glycolysis / Gluconeogenesis" KEGG pathway in the oral microbiome, exhibiting a ≥ 0.2-fold decrease in abundance in transgenic male littermates expressing amyloid precursor protein/presenilin-1 (APP/PS1) compared to age-matched wild-type controls. *EC:6.2.1.1 = Acetate--CoA ligase, *EC:1.1.1.27 = L-lactate dehydrogenase. The red asterisk (*) symbol functions as a crucial visual marker, highlighting specific KEGG enzymes that have been either substantiated through scientific inquiry or are hypothesized to potentially play pivotal roles in the onset or progression of Alzheimer’s disease (AD). None of the enzymes enriched in this pathway in oral WT microbiome showed increased abundance in the gut microbiome of WT mice relative to APP/PS1 mice. Furthermore, a substantial number of these enzymes were completely undetected in the gut microbial ecosystem. Consequently, these enzymes were not considered from our most recent study examining the influence of the gut microbiome on Alzheimer's disease (AD).


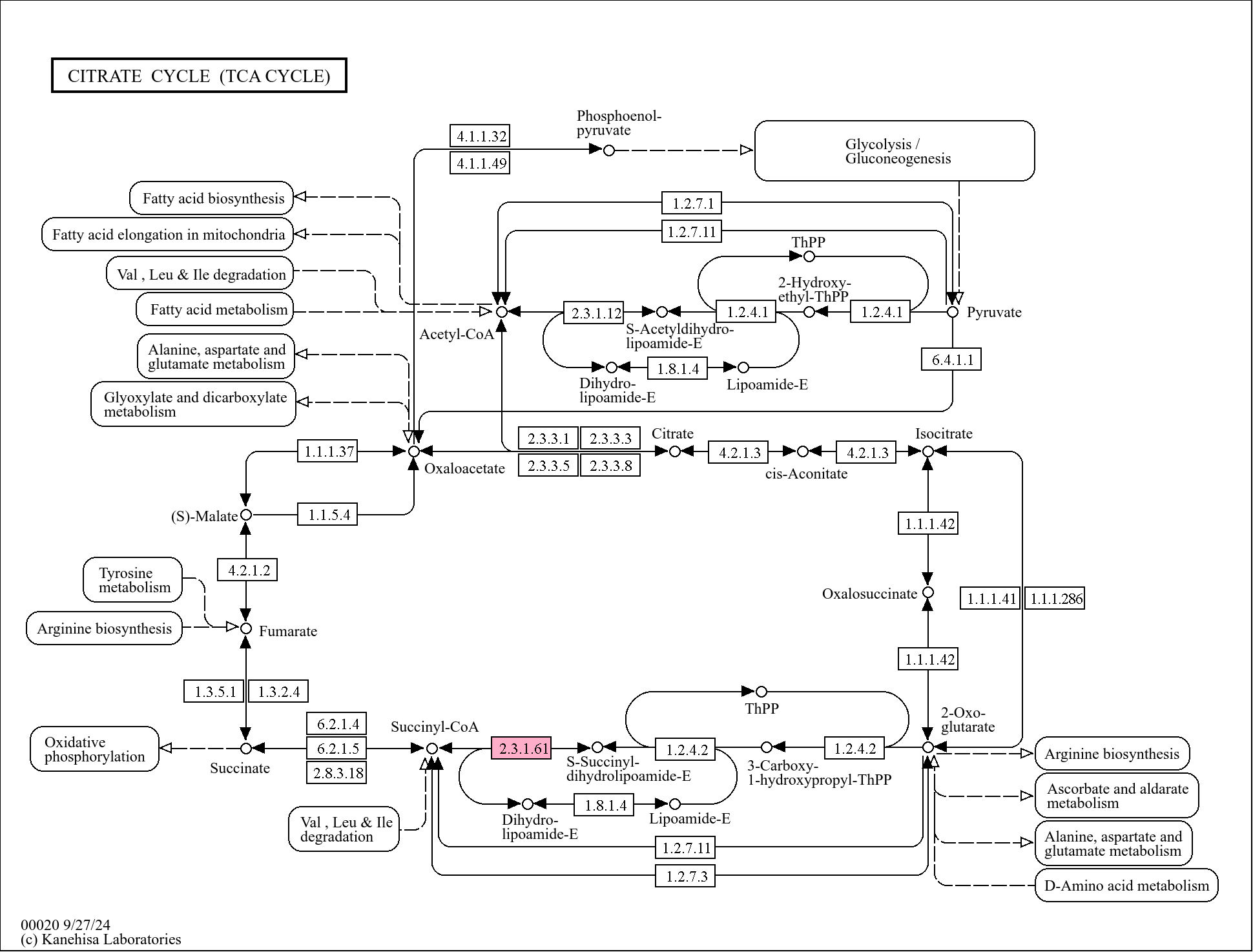
Fig. S6. Enzymatic profile of the "Citrate cycle (TCA cycle)" KEGG pathway in the oral microbiome, exhibiting a ≥ 0.2-fold decrease in abundance in transgenic male littermates expressing amyloid precursor protein/presenilin-1 (APP/PS1) compared to age-matched wild-type controls. EC:2.3.1.61 = Dihydrolipoyllysine-residue succinyltransferase. None of the enzymes enriched in this pathway in oral WT microbiome showed increased abundance in the gut microbiome of WT mice relative to APP/PS1 mice. Furthermore, a substantial number of these enzymes were completely undetected in the gut microbial ecosystem. Consequently, these enzymes were not considered from our most recent study examining the influence of the gut microbiome on Alzheimer's disease (AD).


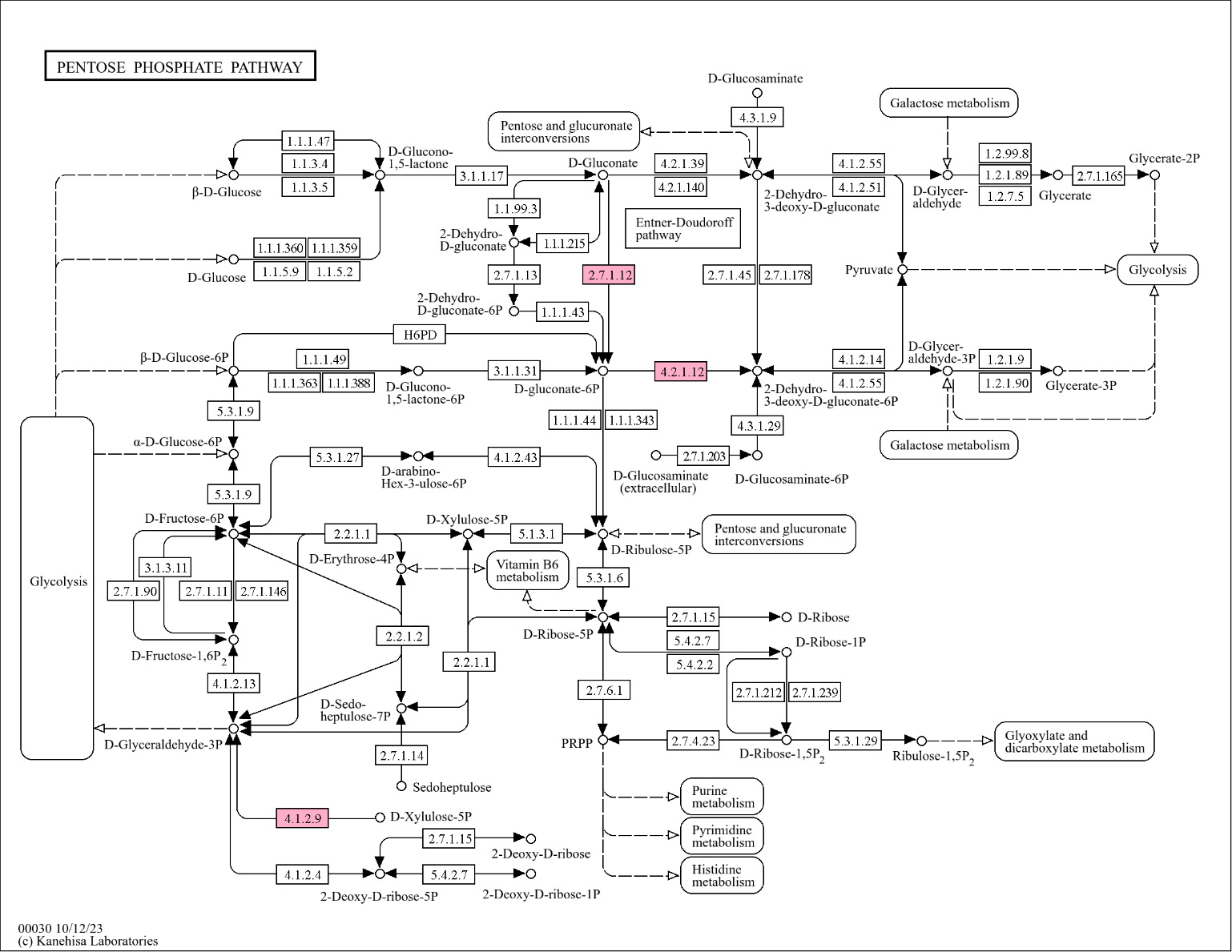
Fig. S7. Enzymatic profile of the "Pentose phosphate pathway" KEGG pathway in the oral microbiome, exhibiting a ≥ 0.2-fold decrease in abundance in transgenic male littermates expressing amyloid precursor protein/presenilin-1 (APP/PS1) compared to age-matched wild-type controls. EC:2.7.1.12 = Gluconokinase, EC:4.1.2.9 = Phosphoketolase, EC:4.2.1.12 = Phosphogluconate dehydratase. None of the enzymes enriched in this pathway in oral WT microbiome showed increased abundance in the gut microbiome of WT mice relative to APP/PS1 mice. Furthermore, a substantial number of these enzymes were completely undetected in the gut microbial ecosystem. Consequently, these enzymes were not considered from our most recent study examining the influence of the gut microbiome on Alzheimer's disease (AD).


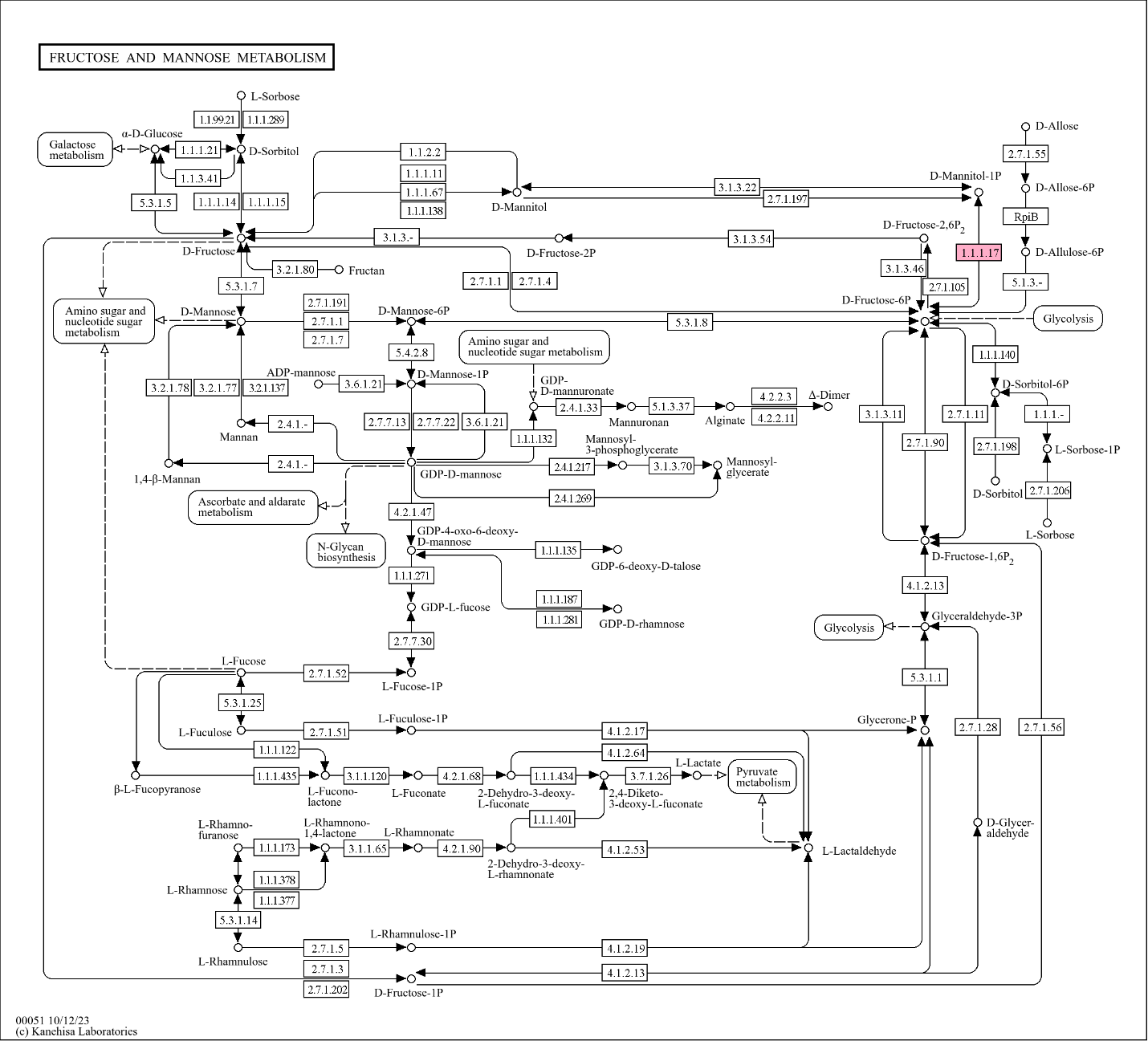
Fig. S8. Enzymatic profile of the "Fructose and mannose metabolism" KEGG pathway in the oral microbiome, exhibiting a ≥ 0.2-fold decrease in abundance in transgenic male littermates expressing amyloid precursor protein/presenilin-1 (APP/PS1) compared to age-matched wild-type controls. EC:1.1.1.17 = Mannitol-1-phosphate 5-dehydrogenase. None of the enzymes enriched in this pathway in oral WT microbiome showed increased abundance in the gut microbiome of WT mice relative to APP/PS1 mice. Furthermore, a substantial number of these enzymes were completely undetected in the gut microbial ecosystem. Consequently, these enzymes were not considered from our most recent study examining the influence of the gut microbiome on Alzheimer's disease (AD).


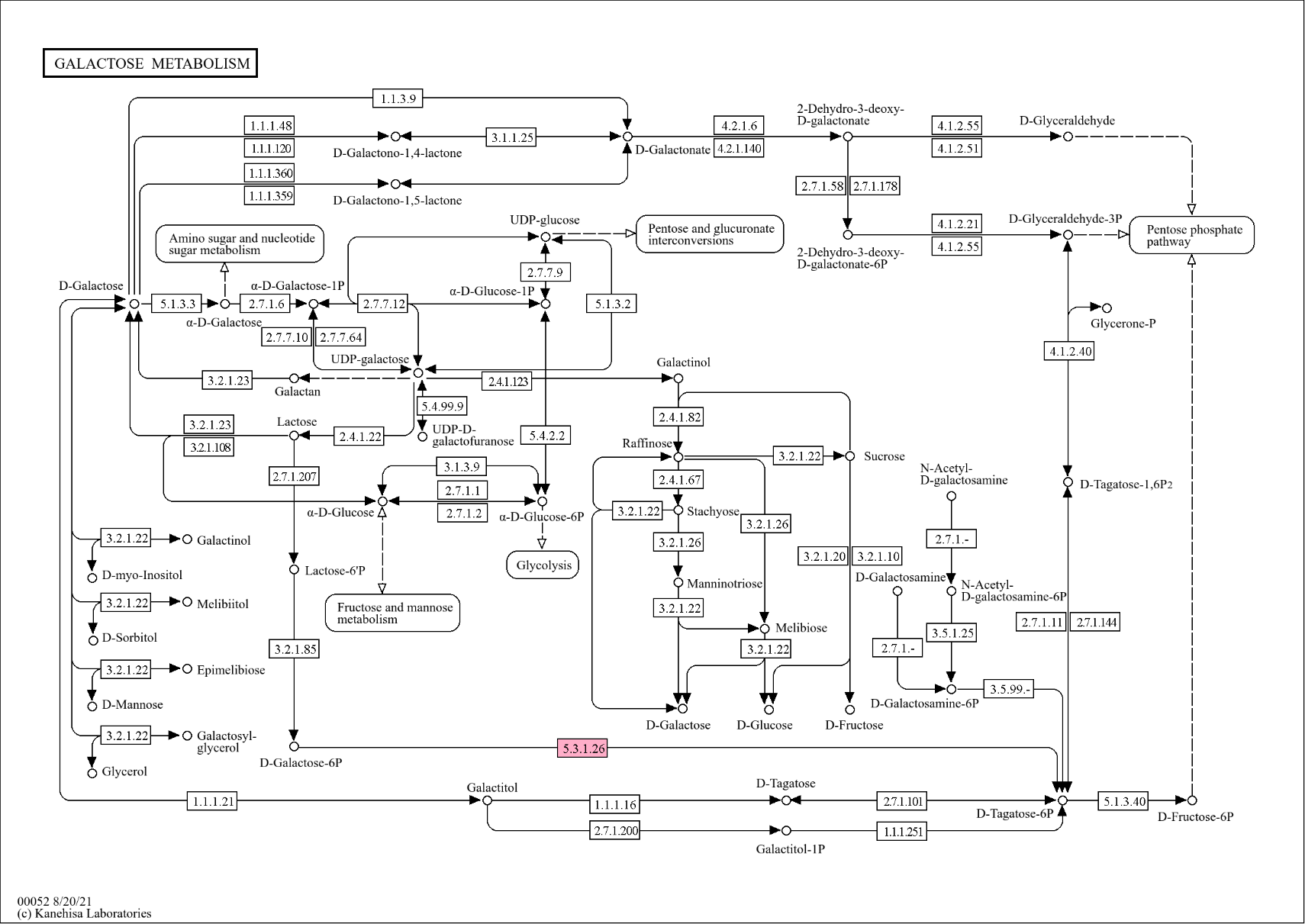
Fig. S9. Enzymatic profile of the "Galactose metabolism" KEGG pathway in the oral microbiome, exhibiting a ≥ 0.2-fold decrease in abundance in transgenic male littermates expressing amyloid precursor protein/presenilin-1 (APP/PS1) compared to age-matched wild-type controls. EC:5.3.1.26 = Galactose-6-phosphate isomerase. None of the enzymes enriched in this pathway in oral WT microbiome showed increased abundance in the gut microbiome of WT mice relative to APP/PS1 mice. Furthermore, a substantial number of these enzymes were completely undetected in the gut microbial ecosystem. Consequently, these enzymes were not considered from our most recent study examining the influence of the gut microbiome on Alzheimer's disease (AD).


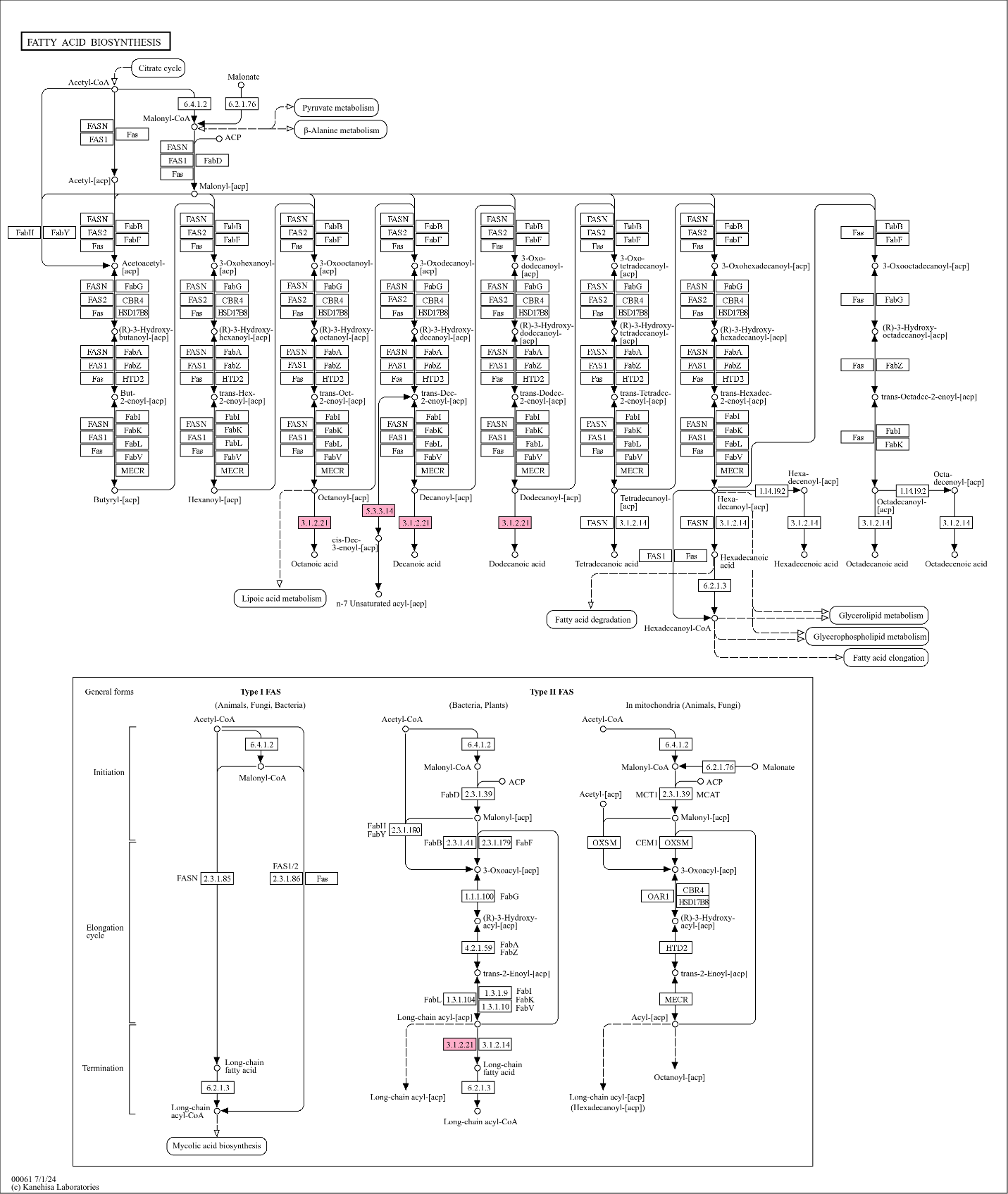
Fig. S10. Enzymatic profile of the "Fatty acid biosynthesis" KEGG pathway in the oral microbiome, exhibiting a ≥ 0.2-fold decrease in abundance in transgenic male littermates expressing amyloid precursor protein/presenilin-1 (APP/PS1) compared to age-matched wild-type controls. EC:3.1.2.21 = Dodecanoyl-[acyl-carrier-protein] hydrolase; EC:5.3.3.14 = Trans-2-decenoyl-[acyl-carrier-protein] isomerase. None of the enzymes enriched in this pathway in oral WT microbiome showed increased abundance in the gut microbiome of WT mice relative to APP/PS1 mice. Furthermore, a substantial number of these enzymes were completely undetected in the gut microbial ecosystem. Consequently, these enzymes were not considered from our most recent study examining the influence of the gut microbiome on Alzheimer's disease (AD).


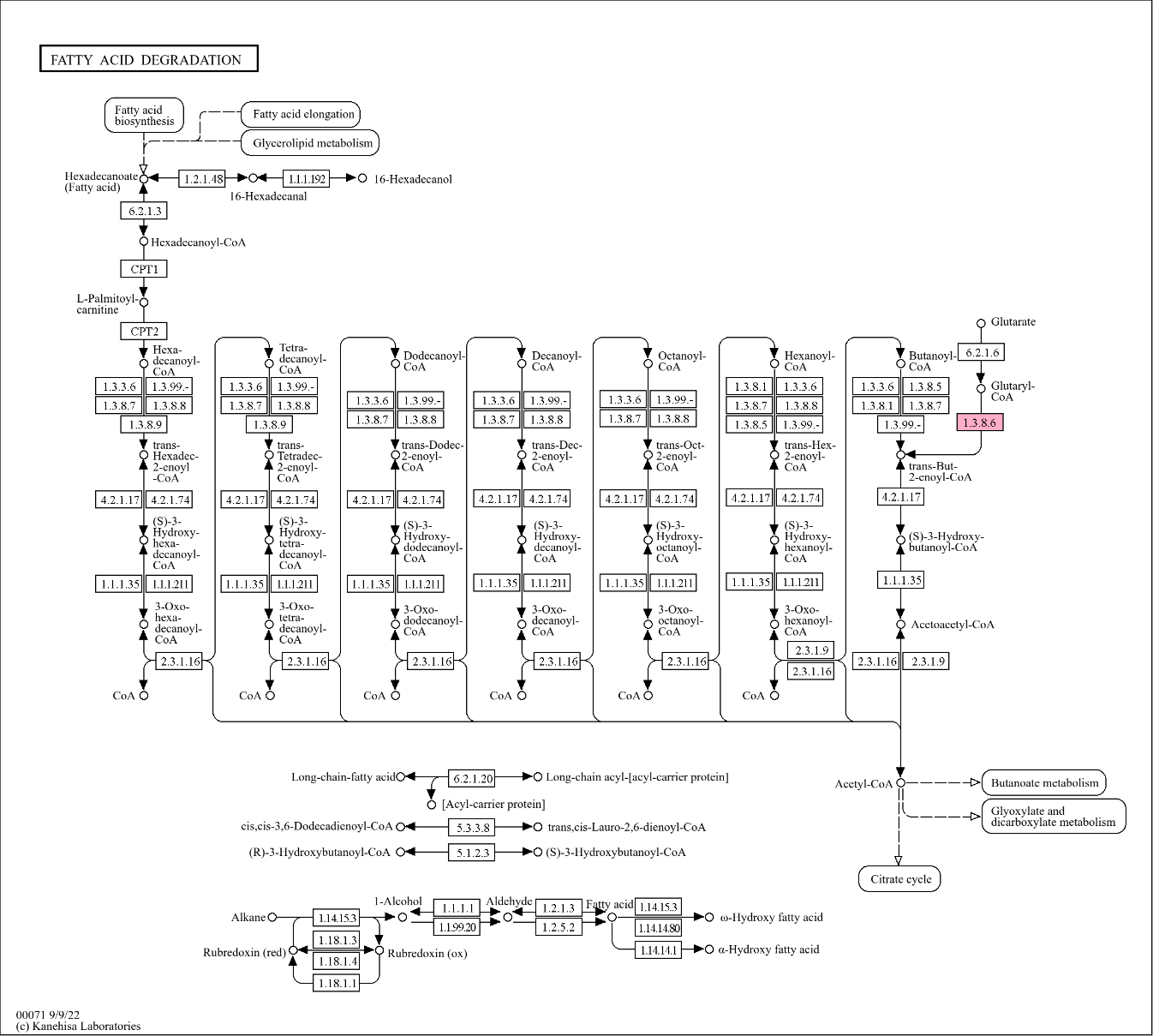
Fig. S11. Enzymatic profile of the "Fatty acid degradation" KEGG pathway in the oral microbiome, exhibiting a ≥ 0.2-fold decrease in abundance in transgenic male littermates expressing amyloid precursor protein/presenilin-1 (APP/PS1) compared to age-matched wild-type controls. EC:1.3.8.6 = Glutaryl-CoA dehydrogenase (ETF). None of the enzymes enriched in this pathway in oral WT microbiome showed increased abundance in the gut microbiome of WT mice relative to APP/PS1 mice. Furthermore, a substantial number of these enzymes were completely undetected in the gut microbial ecosystem. Consequently, these enzymes were not considered from our most recent study examining the influence of the gut microbiome on Alzheimer's disease (AD).


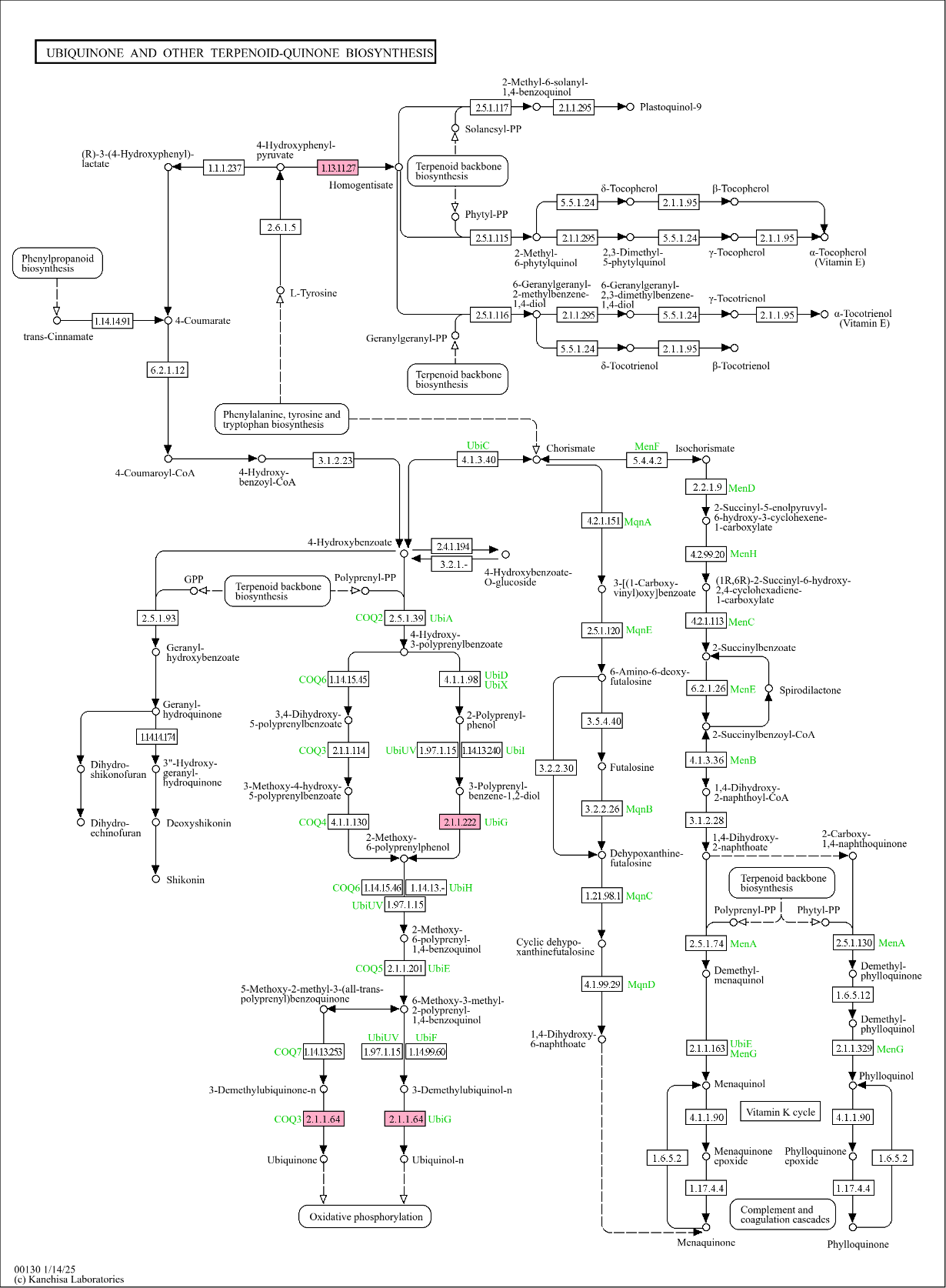
Fig. S12. Enzymatic profile of the "Ubiquinone and other terpenoid-quinone biosynthesis" KEGG pathway in the oral microbiome, exhibiting a ≥ 0.2-fold decrease in abundance in transgenic male littermates expressing amyloid precursor protein/presenilin-1 (APP/PS1) compared to age-matched wild-type controls. EC:1.13.11.27 = 4-hydroxyphenylpyruvate dioxygenase; EC:2.1.1.222 = 2-polyprenyl-6-hydroxyphenol methylase; EC:2.1.1.64 = 3-demethylubiquinol 3-O-methyltransferase. None of the enzymes enriched in this pathway in oral WT microbiome showed increased abundance in the gut microbiome of WT mice relative to APP/PS1 mice. Furthermore, a substantial number of these enzymes were completely undetected in the gut microbial ecosystem. Consequently, these enzymes were not considered from our most recent study examining the influence of the gut microbiome on Alzheimer's disease (AD).


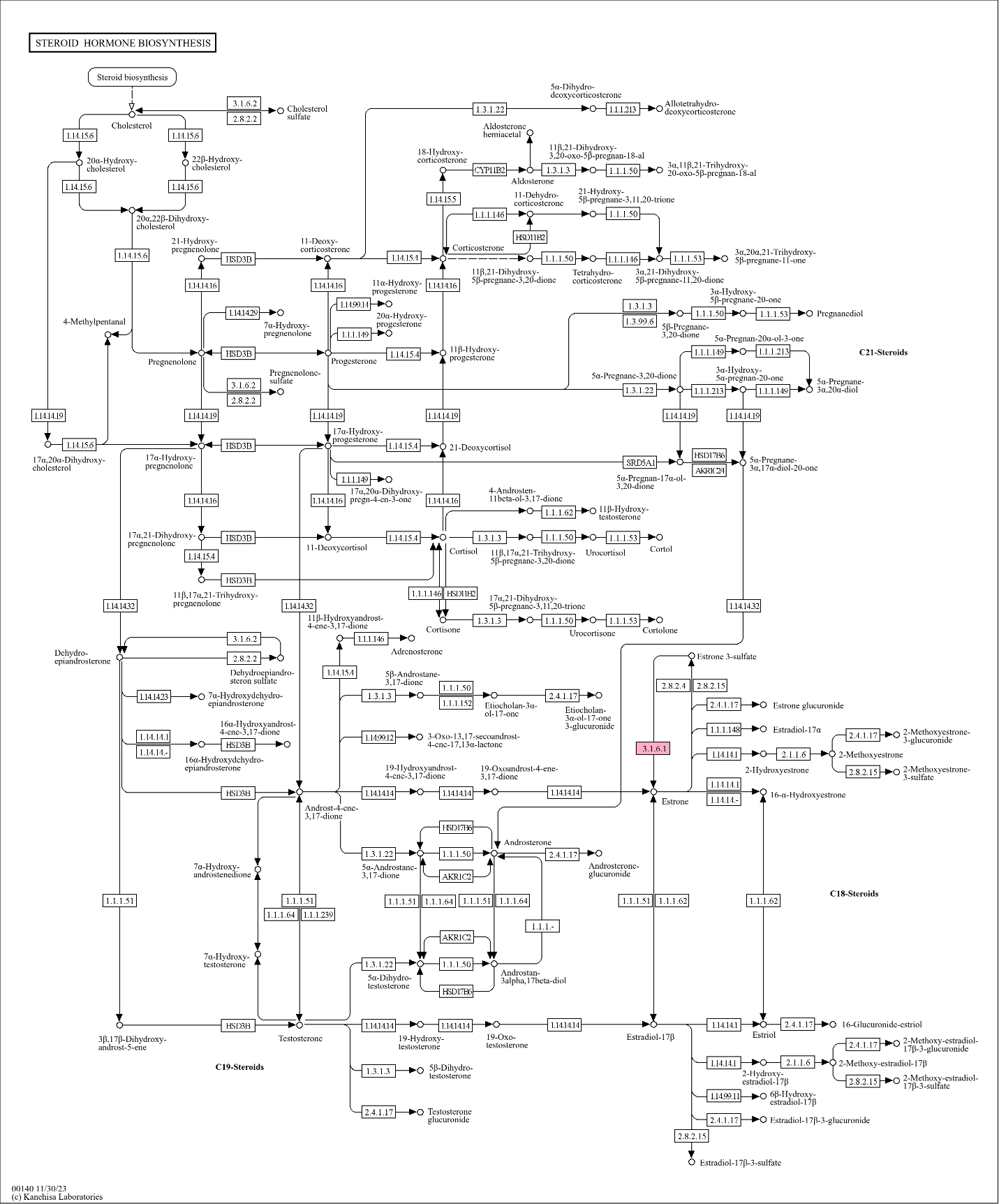
Fig. S13. Enzymatic profile of the "Steroid hormone biosynthesis" KEGG pathway in the oral microbiome, exhibiting a ≥ 0.2-fold decrease in abundance in transgenic male littermates expressing amyloid precursor protein/presenilin-1 (APP/PS1) compared to age-matched wild-type controls. EC:3.1.6.1 = Arylsulfatase. None of the enzymes enriched in this pathway in oral WT microbiome showed increased abundance in the gut microbiome of WT mice relative to APP/PS1 mice. Furthermore, a substantial number of these enzymes were completely undetected in the gut microbial ecosystem. Consequently, these enzymes were not considered from our most recent study examining the influence of the gut microbiome on Alzheimer's disease (AD).


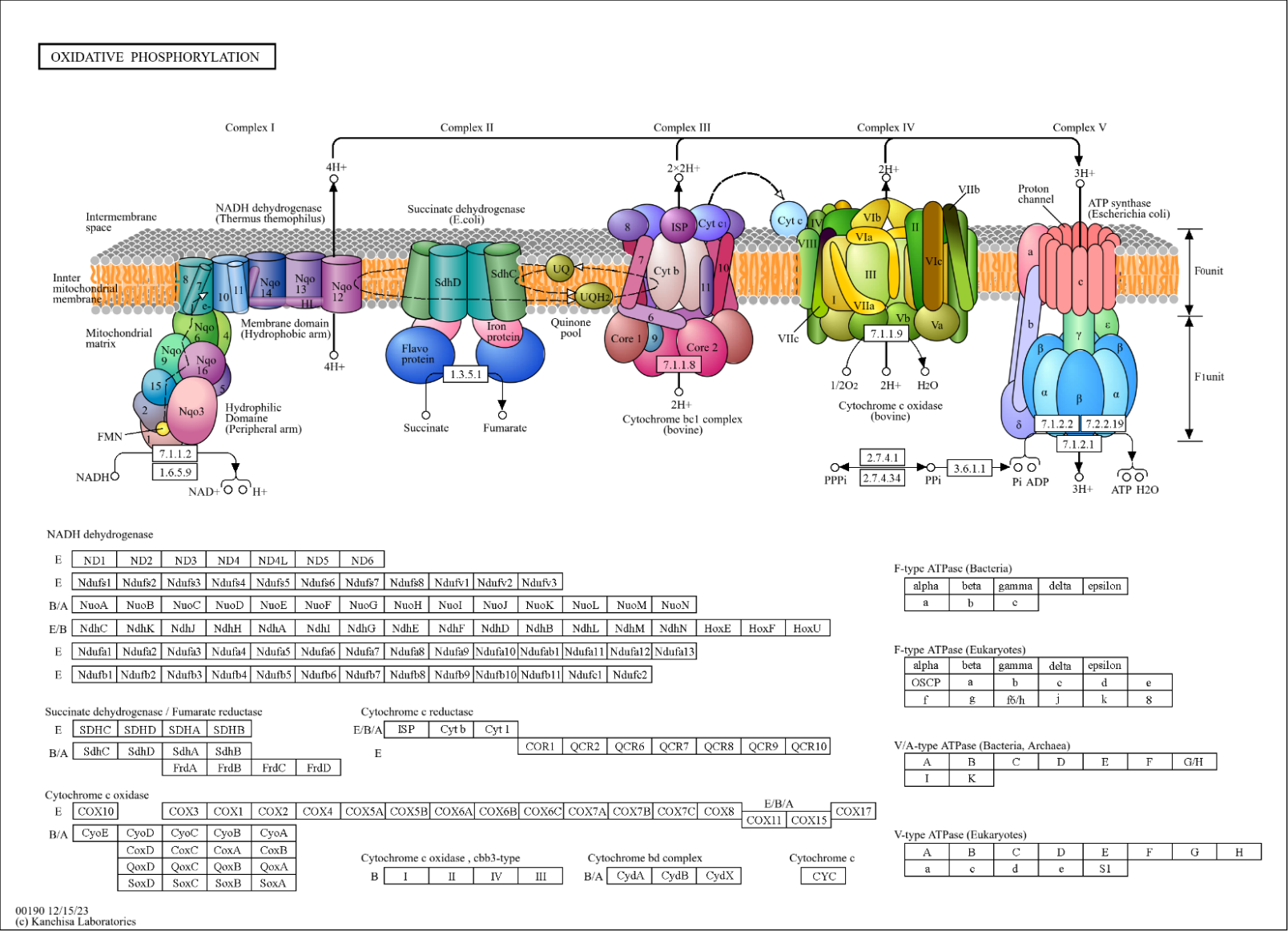
Fig. S14. Enzymatic profile of the "Oxidative phosphorylation" KEGG pathway in the oral microbiome, exhibiting a ≥ 0.2-fold decrease in abundance in transgenic male littermates expressing amyloid precursor protein/presenilin-1 (APP/PS1) compared to age-matched wild-type controls. *EC:1.10.2.2 (EC:7.1.1.8) = Quinol--cytochrome-c reductase. The red asterisk (*) symbol functions as a crucial visual marker, highlighting specific KEGG enzymes that have been either substantiated through scientific inquiry or are hypothesized to potentially play pivotal roles in the onset or progression of Alzheimer’s disease (AD). None of the enzymes enriched in this pathway in oral WT microbiome showed increased abundance in the gut microbiome of WT mice relative to APP/PS1 mice. Furthermore, a substantial number of these enzymes were completely undetected in the gut microbial ecosystem. Consequently, these enzymes were not considered from our most recent study examining the influence of the gut microbiome on Alzheimer's disease (AD).


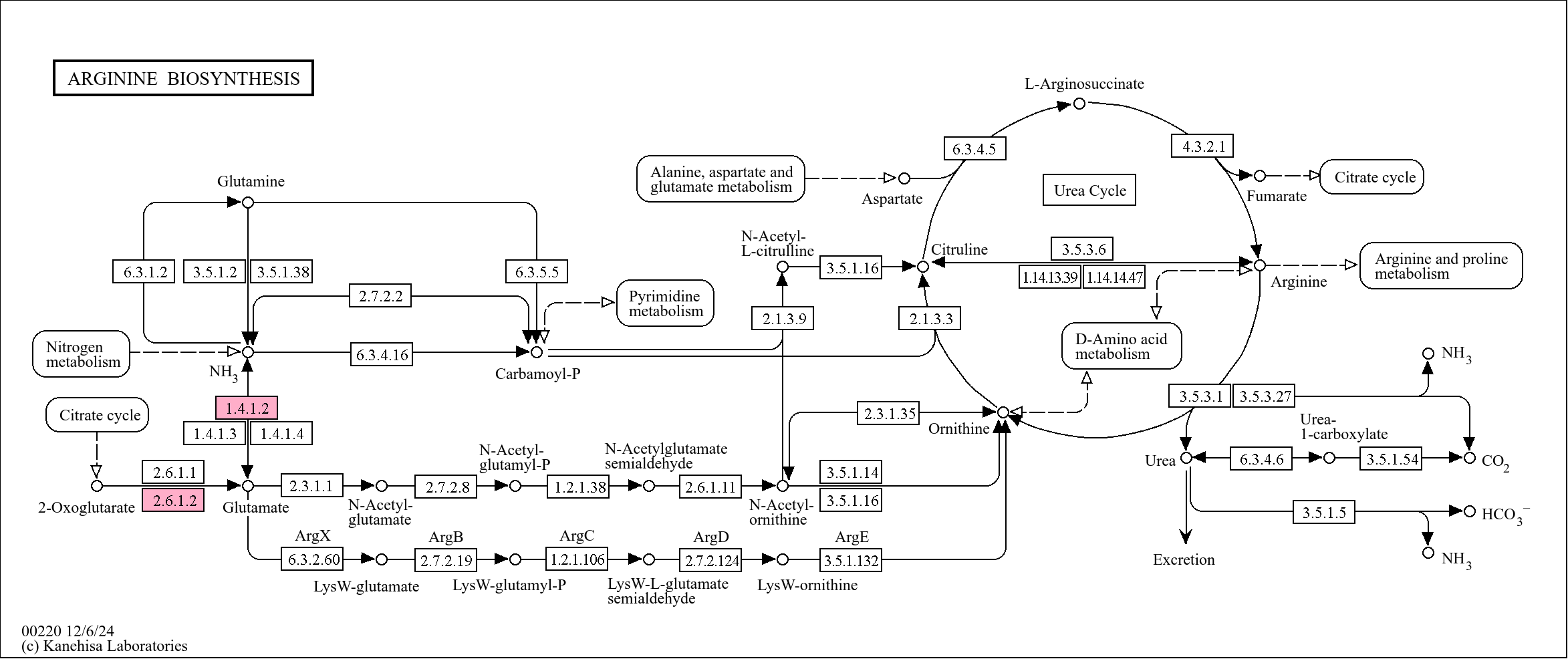
Fig. S15. Enzymatic profile of the "Arginine biosynthesis" KEGG pathway in the oral microbiome, exhibiting a ≥ 0.2-fold decrease in abundance in transgenic male littermates expressing amyloid precursor protein/presenilin-1 (APP/PS1) compared to age-matched wild-type controls. EC:2.6.1.2 = Alanine transaminase; *EC:1.4.1.2 = Glutamate dehydrogenase. The red asterisk (*) symbol functions as a crucial visual marker, highlighting specific KEGG enzymes that have been either substantiated through scientific inquiry or are hypothesized to potentially play pivotal roles in the onset or progression of Alzheimer’s disease (AD). None of the enzymes enriched in this pathway in oral WT microbiome showed increased abundance in the gut microbiome of WT mice relative to APP/PS1 mice. Furthermore, a substantial number of these enzymes were completely undetected in the gut microbial ecosystem. Consequently, these enzymes were not considered from our most recent study examining the influence of the gut microbiome on Alzheimer's disease (AD).


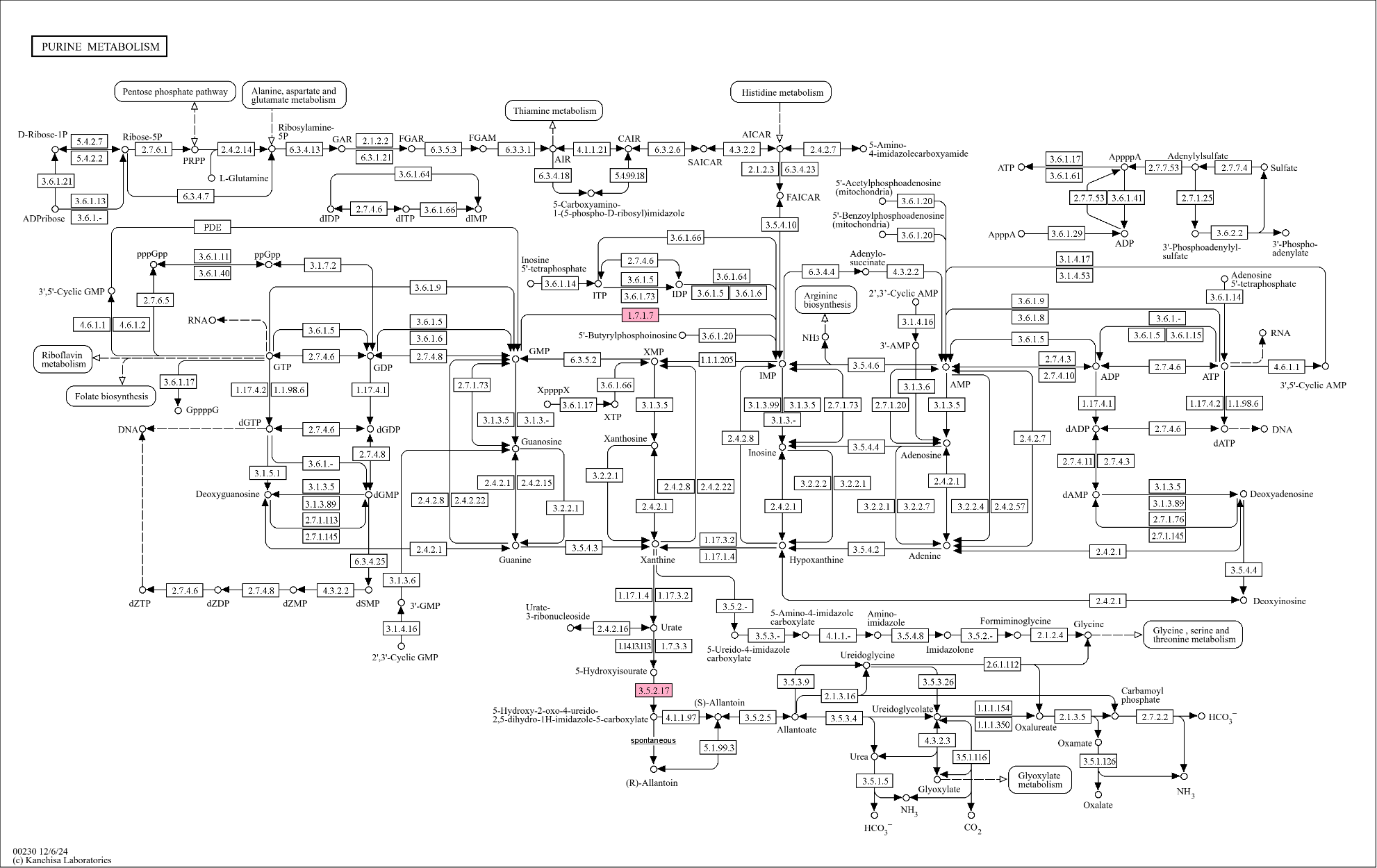
Fig. S16. Enzymatic profile of the "Purine metabolism" KEGG pathway in the oral microbiome, exhibiting a ≥ 0.2-fold decrease in abundance in transgenic male littermates expressing amyloid precursor protein/presenilin-1 (APP/PS1) compared to age-matched wild-type controls. EC:1.7.1.7 = GMP reductase; EC:3.5.2.17 = Hydroxyisourate hydrolase. None of the enzymes enriched in this pathway in oral WT microbiome showed increased abundance in the gut microbiome of WT mice relative to APP/PS1 mice. Furthermore, a substantial number of these enzymes were completely undetected in the gut microbial ecosystem. Consequently, these enzymes were not considered from our most recent study examining the influence of the gut microbiome on Alzheimer's disease (AD).


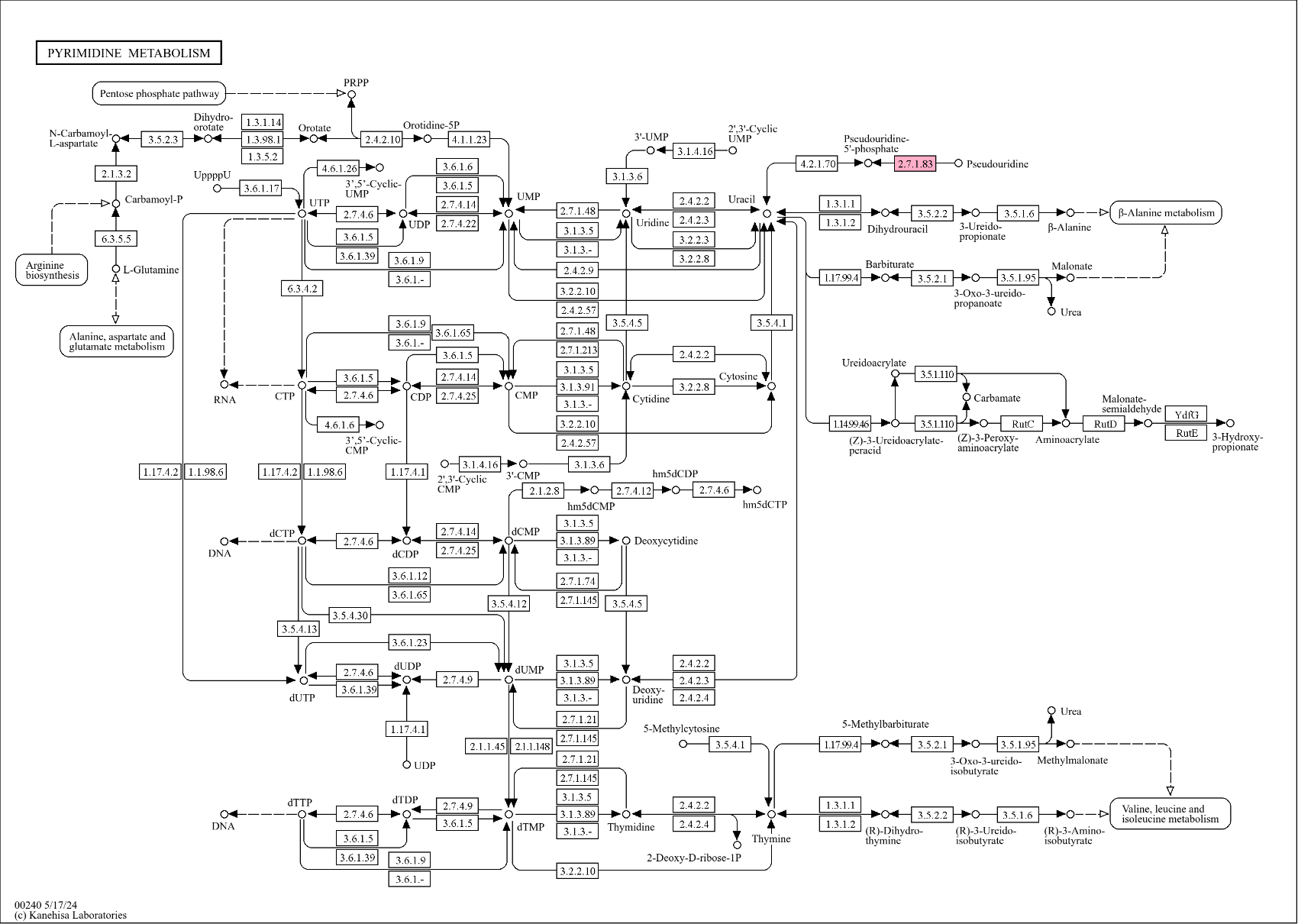
Fig. S17. Enzymatic profile of the "Pyrimidine metabolism" KEGG pathway in the oral microbiome, exhibiting a ≥ 0.2-fold decrease in abundance in transgenic male littermates expressing amyloid precursor protein/presenilin-1 (APP/PS1) compared to age-matched wild-type controls. EC:2.7.1.83 = Pseudouridine kinase. None of the enzymes enriched in this pathway in oral WT microbiome showed increased abundance in the gut microbiome of WT mice relative to APP/PS1 mice. Furthermore, a substantial number of these enzymes were completely undetected in the gut microbial ecosystem. Consequently, these enzymes were not considered from our most recent study examining the influence of the gut microbiome on Alzheimer's disease (AD).


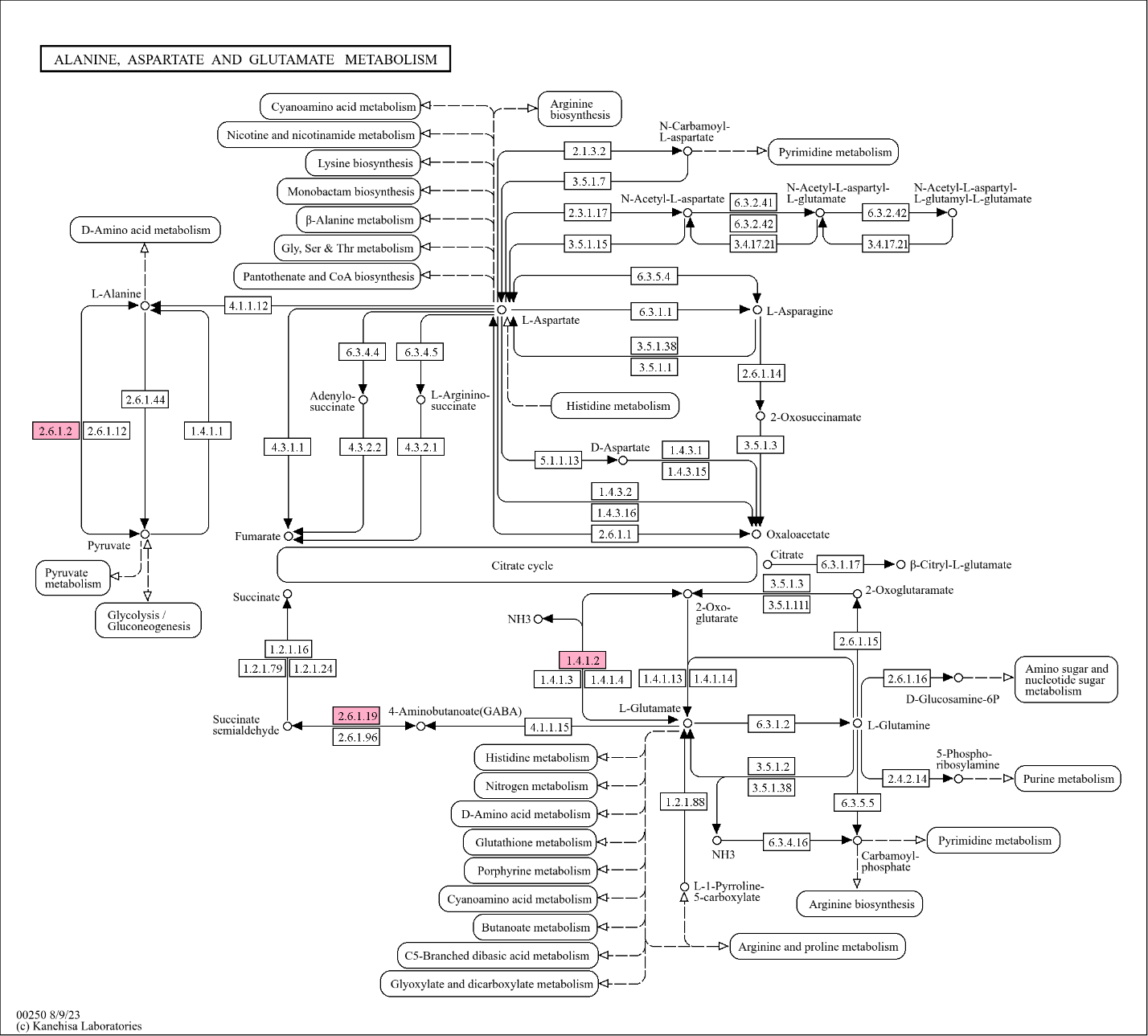
Fig. S18. Enzymatic profile of the "Alanine, aspartate and glutamate metabolism" KEGG pathway in the oral microbiome, exhibiting a ≥ 0.2-fold decrease in abundance in transgenic male littermates expressing amyloid precursor protein/presenilin-1 (APP/PS1) compared to age-matched wild-type controls. EC:2.6.1.19 = 4-aminobutyrate--2-oxoglutarate transaminase; EC:2.6.1.2 = Alanine transaminase; *EC:1.4.1.2 = Glutamate dehydrogenase. The red asterisk (*) symbol functions as a crucial visual marker, highlighting specific KEGG enzymes that have been either substantiated through scientific inquiry or are hypothesized to potentially play pivotal roles in the onset or progression of Alzheimer’s disease (AD). None of the enzymes enriched in this pathway in oral WT microbiome showed increased abundance in the gut microbiome of WT mice relative to APP/PS1 mice. Furthermore, a substantial number of these enzymes were completely undetected in the gut microbial ecosystem. Consequently, these enzymes were not considered from our most recent study examining the influence of the gut microbiome on Alzheimer's disease (AD).


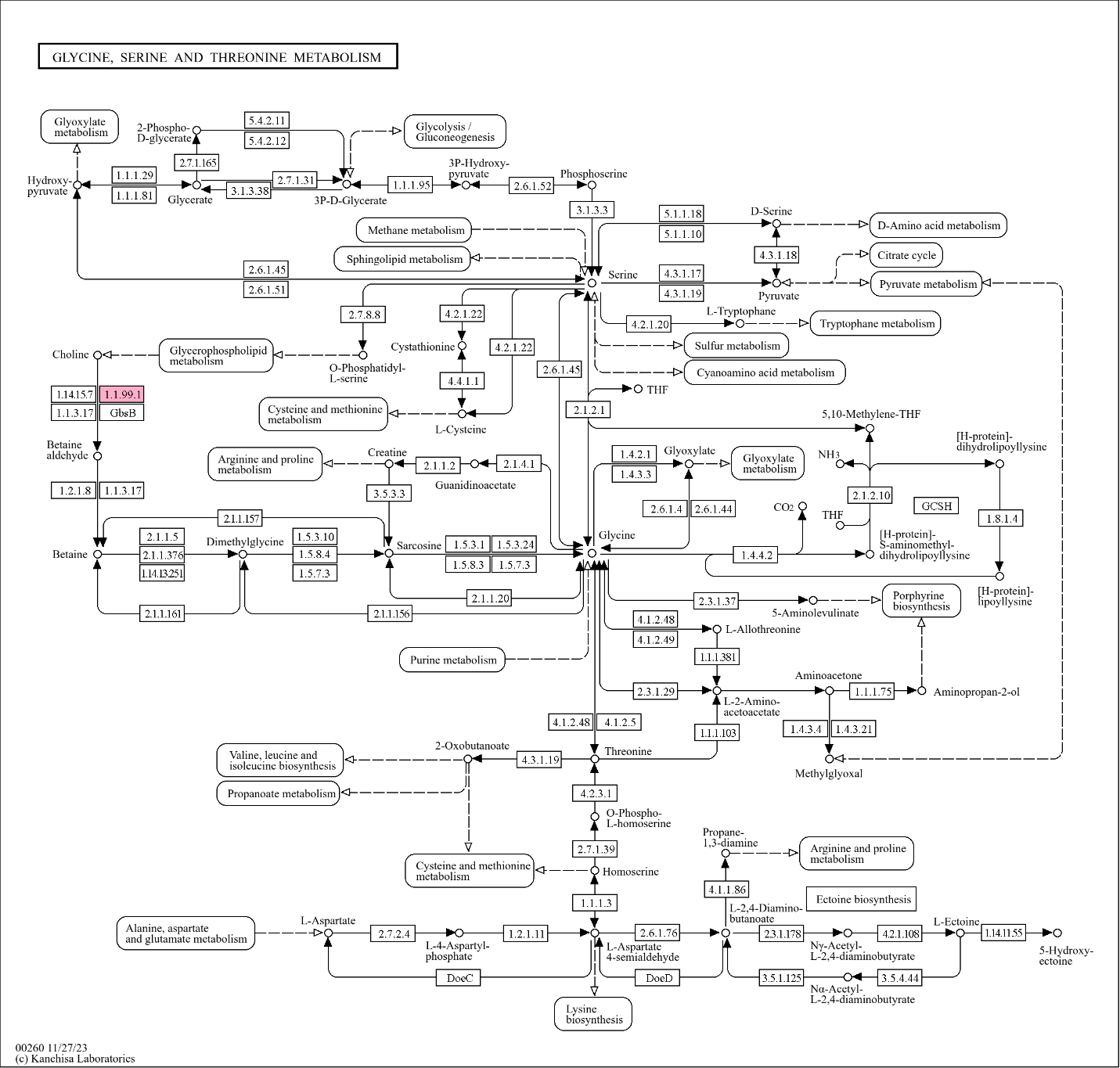
Fig. S19. Enzymatic profile of the "Glycine, serine and threonine metabolism" KEGG pathway in the oral microbiome, exhibiting a ≥ 0.2-fold decrease in abundance in transgenic male littermates expressing amyloid precursor protein/presenilin-1 (APP/PS1) compared to age-matched wild-type controls. *EC:1.1.99.1 = Choline dehydrogenase. The red asterisk (*) symbol functions as a crucial visual marker, highlighting specific KEGG enzymes that have been either substantiated through scientific inquiry or are hypothesized to potentially play pivotal roles in the onset or progression of Alzheimer’s disease (AD). None of the enzymes enriched in this pathway in oral WT microbiome showed increased abundance in the gut microbiome of WT mice relative to APP/PS1 mice. Furthermore, a substantial number of these enzymes were completely undetected in the gut microbial ecosystem. Consequently, these enzymes were not considered from our most recent study examining the influence of the gut microbiome on Alzheimer's disease (AD).


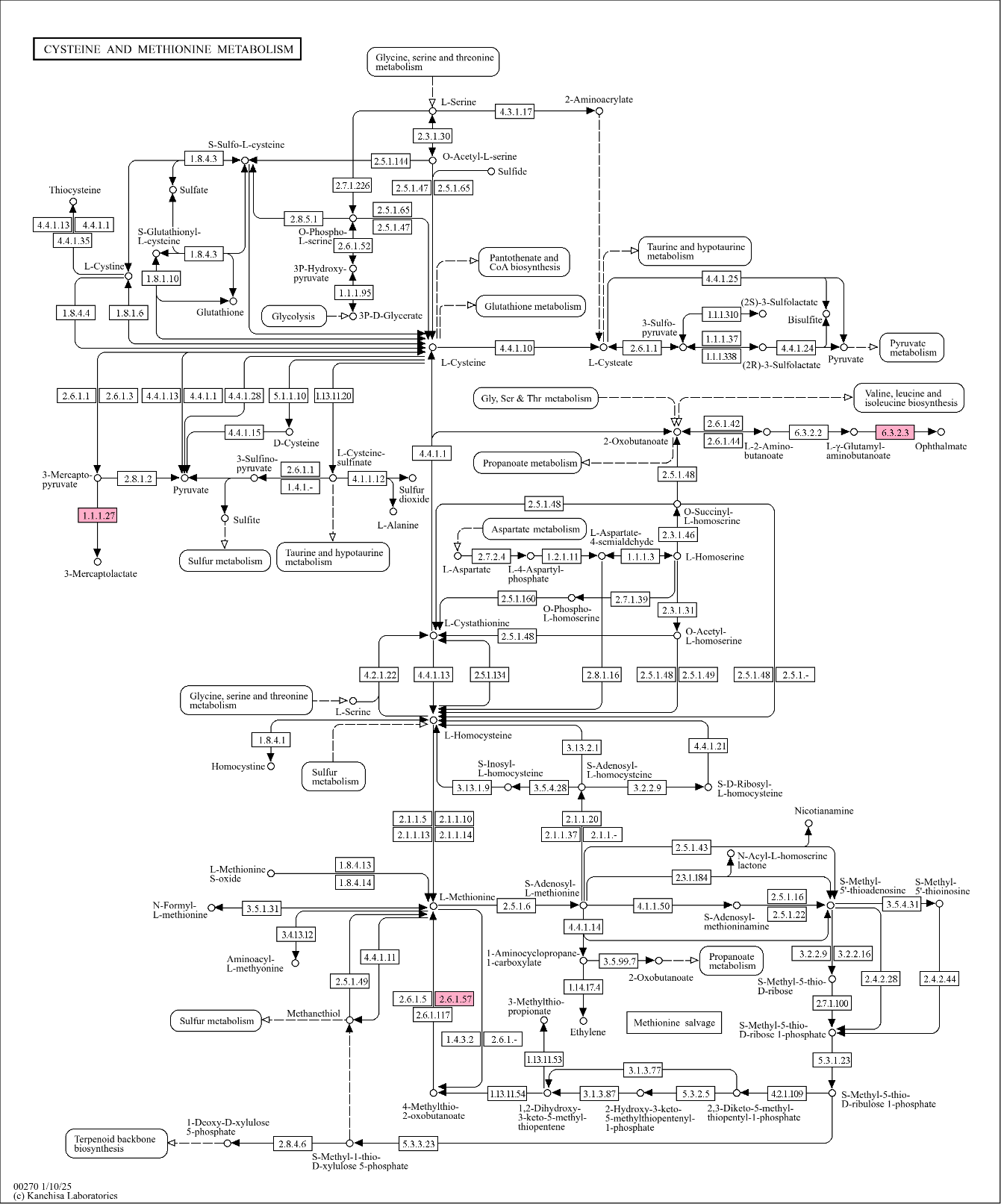
Fig. S20. Enzymatic profile of the "Cysteine and methionine metabolism" KEGG pathway in the oral microbiome, exhibiting a ≥ 0.2-fold decrease in abundance in transgenic male littermates expressing amyloid precursor protein/presenilin-1 (APP/PS1) compared to age-matched wild-type controls. EC:2.6.1.57 = Aromatic-amino-acid transaminase; EC:6.3.2.3 = Glutathione synthase; *EC:1.1.1.27 = L-lactate dehydrogenase. The red asterisk (*) symbol functions as a crucial visual marker, highlighting specific KEGG enzymes that have been either substantiated through scientific inquiry or are hypothesized to potentially play pivotal roles in the onset or progression of Alzheimer’s disease (AD). None of the enzymes enriched in this pathway in oral WT microbiome showed increased abundance in the gut microbiome of WT mice relative to APP/PS1 mice. Furthermore, a substantial number of these enzymes were completely undetected in the gut microbial ecosystem. Consequently, these enzymes were not considered from our most recent study examining the influence of the gut microbiome on Alzheimer's disease (AD).


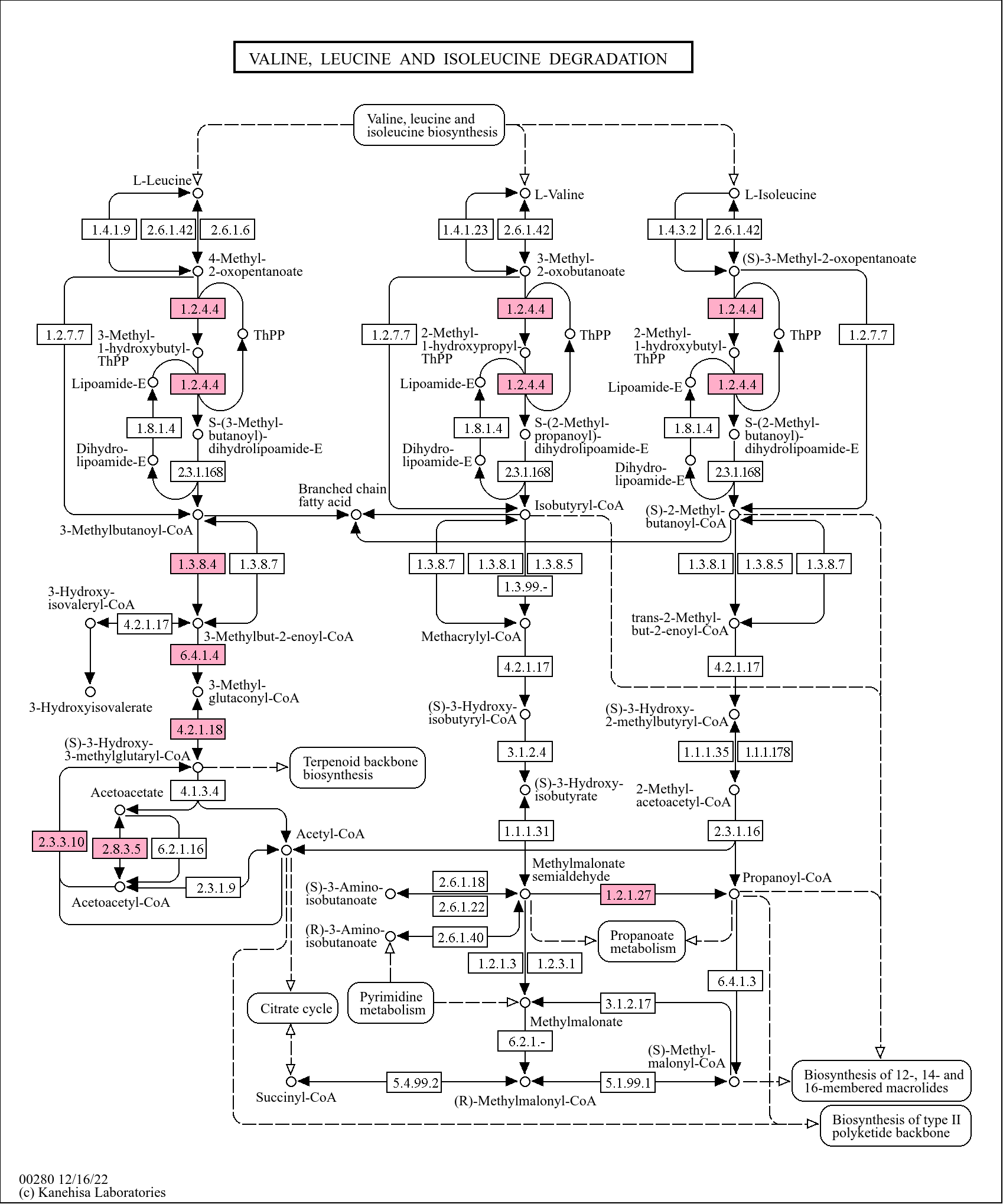
Fig. S21. Enzymatic profile of the "Valine, leucine and isoleucine degradation" KEGG pathway in the oral microbiome, exhibiting a ≥ 0.2-fold decrease in abundance in transgenic male littermates expressing amyloid precursor protein/presenilin-1 (APP/PS1) compared to age-matched wild-type controls. EC:1.2.1.27 = Methylmalonate-semialdehyde dehydrogenase (CoA acylating); EC:1.2.4.4 = 3-Methyl-2-oxobutanoate dehydrogenase (2-methylpropanoyl-transferring); EC:1.3.8.4 = Isovaleryl-CoA dehydrogenase; EC:2.3.3.10 = Hydroxymethylglutaryl-CoA synthase; EC:2.8.3.5 = 3-Oxoacid CoA-transferase; EC:4.2.1.18 = Methylglutaconyl-CoA hydratas; EC:6.4.1.4 = Methylcrotonoyl-CoA carboxylase. None of the enzymes enriched in this pathway in oral WT microbiome showed increased abundance in the gut microbiome of WT mice relative to APP/PS1 mice. Furthermore, a substantial number of these enzymes were completely undetected in the gut microbial ecosystem. Consequently, these enzymes were not considered from our most recent study examining the influence of the gut microbiome on Alzheimer's disease (AD).


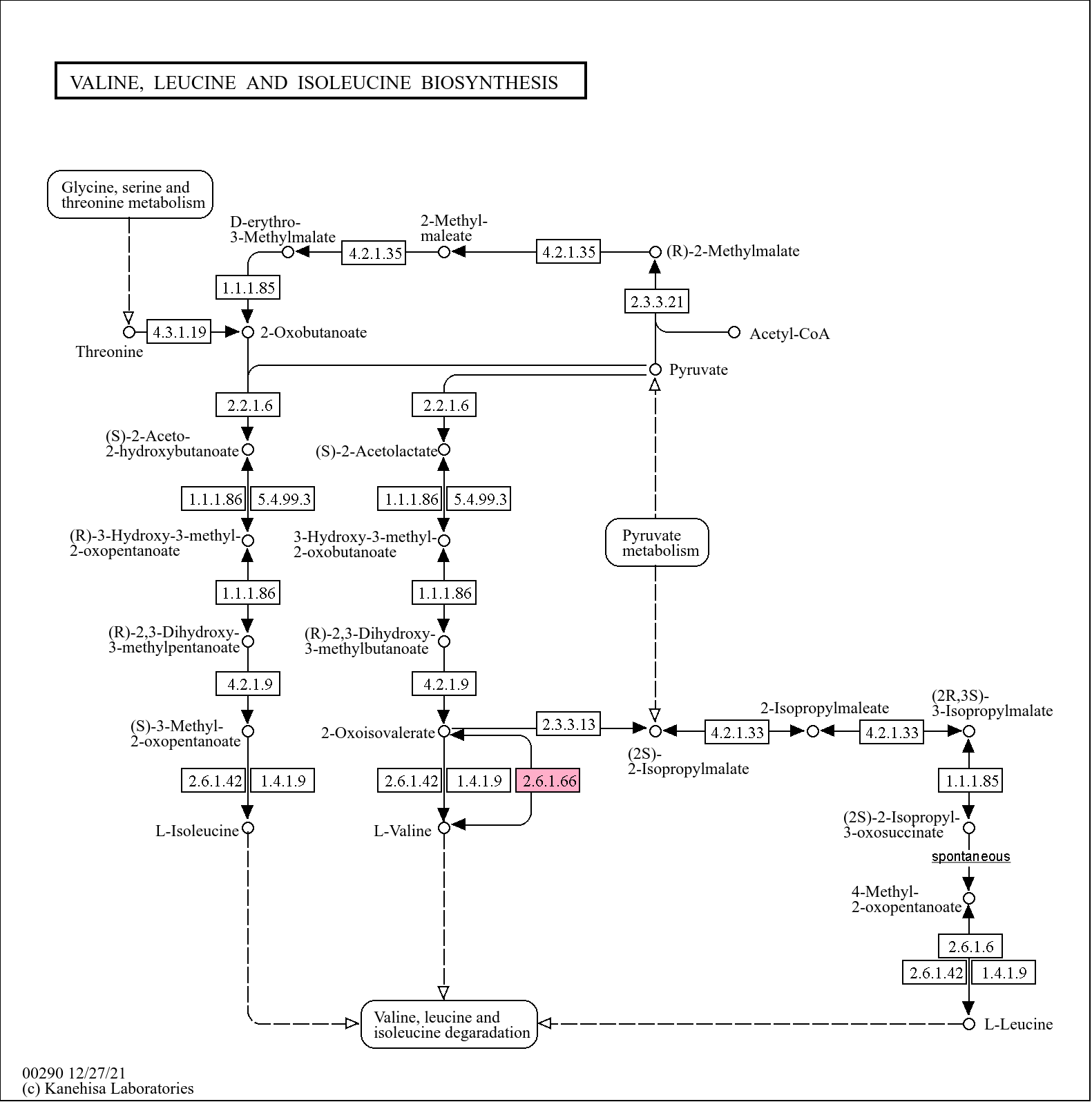
Fig. S22. Enzymatic profile of the "Valine, leucine and isoleucine biosynthesis" KEGG pathway in the oral microbiome, exhibiting a ≥ 0.2-fold decrease in abundance in transgenic male littermates expressing amyloid precursor protein/presenilin-1 (APP/PS1) compared to age-matched wild-type controls. EC:2.6.1.66 = Valine--pyruvate transaminase. None of the enzymes enriched in this pathway in oral WT microbiome showed increased abundance in the gut microbiome of WT mice relative to APP/PS1 mice. Furthermore, a substantial number of these enzymes were completely undetected in the gut microbial ecosystem. Consequently, these enzymes were not considered from our most recent study examining the influence of the gut microbiome on Alzheimer's disease (AD).


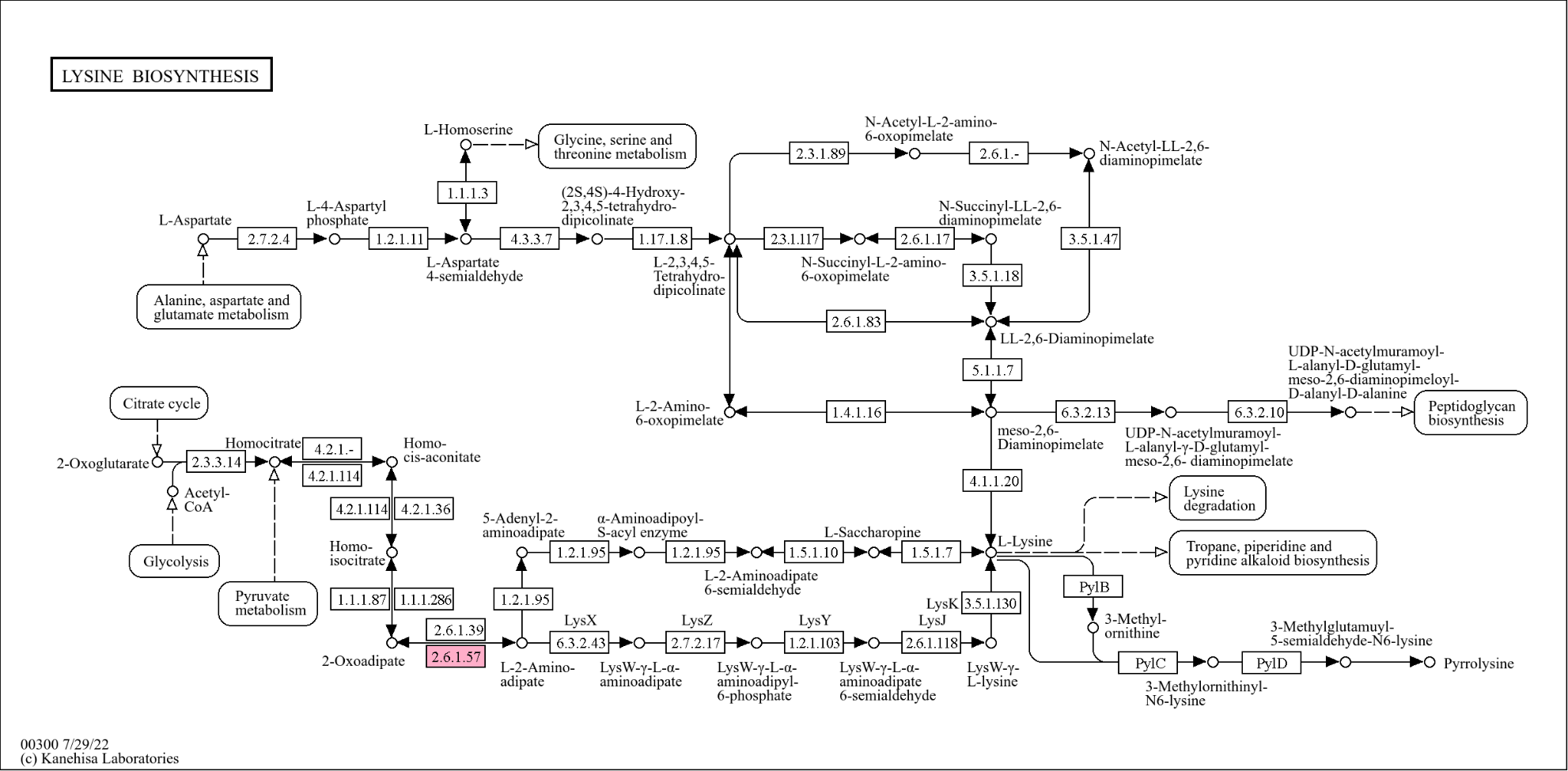
Fig. S23. Enzymatic profile of the "Lysine biosynthesis" KEGG pathway in the oral microbiome, exhibiting a ≥ 0.2-fold decrease in abundance in transgenic male littermates expressing amyloid precursor protein/presenilin-1 (APP/PS1) compared to age-matched wild-type controls. EC:2.6.1.57 = Aromatic-amino-acid transaminase. None of the enzymes enriched in this pathway in oral WT microbiome showed increased abundance in the gut microbiome of WT mice relative to APP/PS1 mice. Furthermore, a substantial number of these enzymes were completely undetected in the gut microbial ecosystem. Consequently, these enzymes were not considered from our most recent study examining the influence of the gut microbiome on Alzheimer's disease (AD).


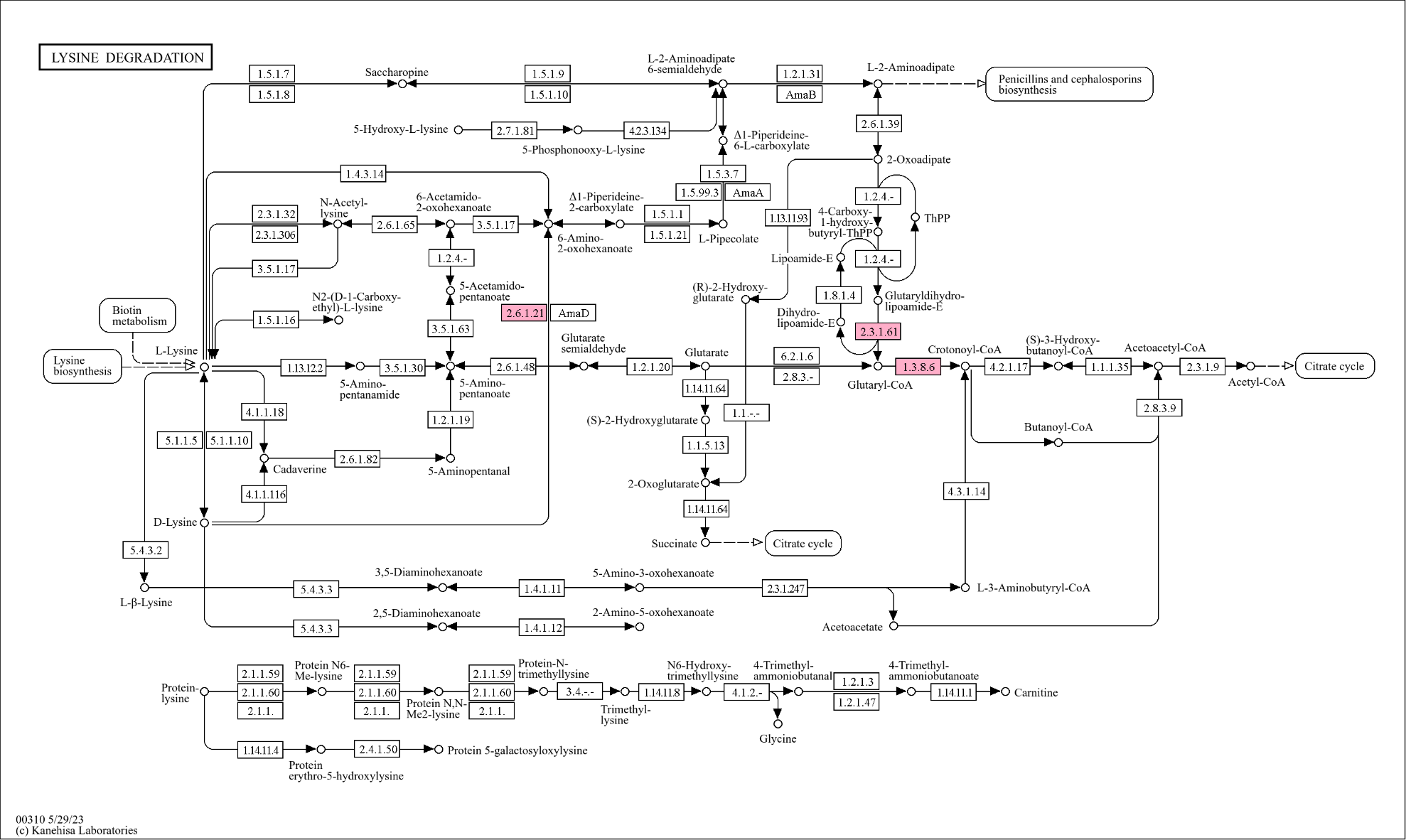
Fig. S24. Enzymatic profile of the "Lysine degradation" KEGG pathway in the oral microbiome, exhibiting a ≥ 0.2-fold decrease in abundance in transgenic male littermates expressing amyloid precursor protein/presenilin-1 (APP/PS1) compared to age-matched wild-type controls. EC:1.3.8.6 = Glutaryl-CoA dehydrogenase (ETF); EC:2.3.1.61 = Dihydrolipoyllysine-residue succinyltransferase, EC:2.6.1.21 = D-amino-acid transaminase. None of the enzymes enriched in this pathway in oral WT microbiome showed increased abundance in the gut microbiome of WT mice relative to APP/PS1 mice. Furthermore, a substantial number of these enzymes were completely undetected in the gut microbial ecosystem. Consequently, these enzymes were not considered from our most recent study examining the influence of the gut microbiome on Alzheimer's disease (AD).


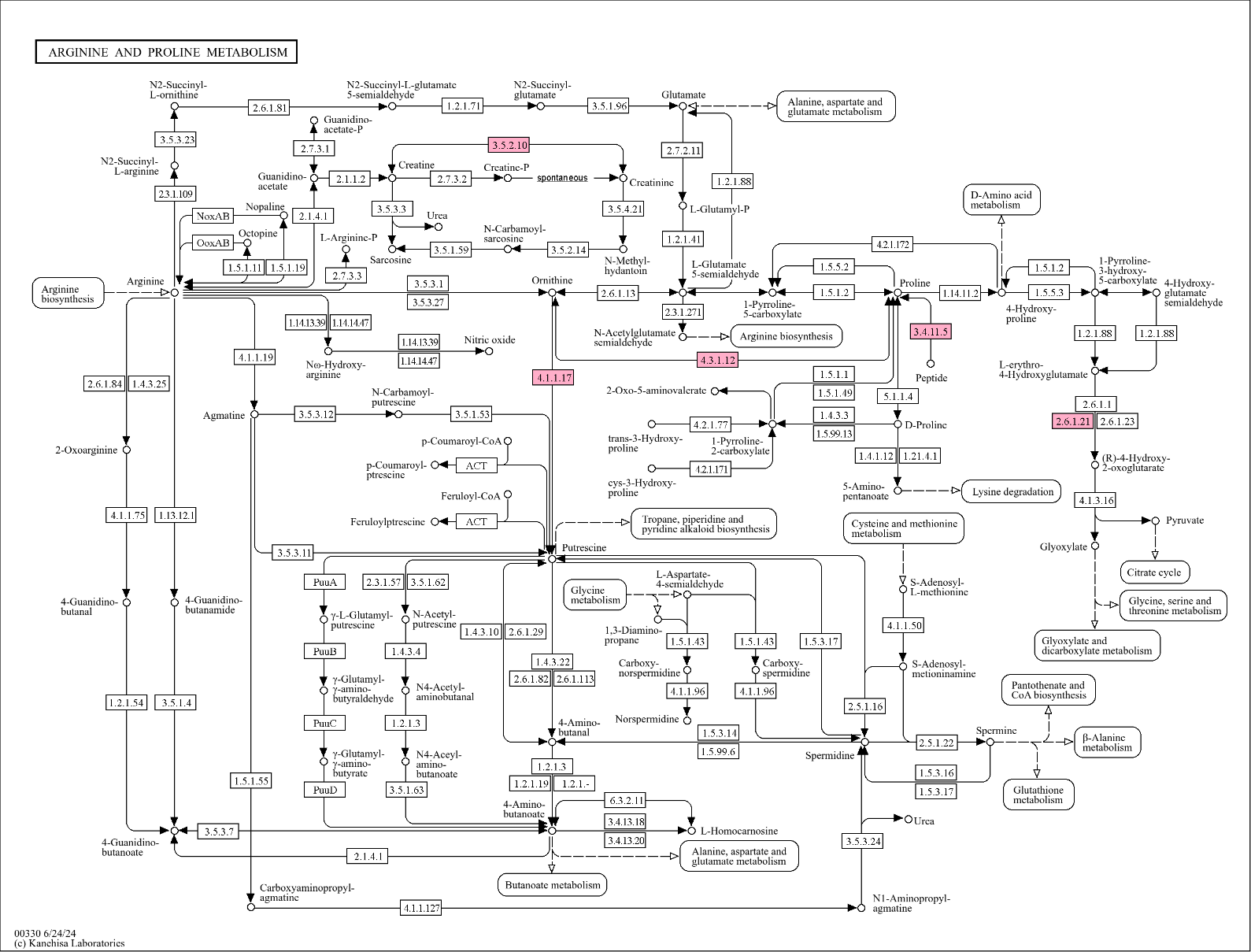
Fig. S25. Enzymatic profile of the "Arginine and proline metabolism" KEGG pathway in the oral microbiome, exhibiting a ≥ 0.2-fold decrease in abundance in transgenic male littermates expressing amyloid precursor protein/presenilin-1 (APP/PS1) compared to age-matched wild-type controls. EC:2.6.1.21 = D-amino-acid transaminase; EC:3.4.11.5 = Prolyl aminopeptidase; EC:3.5.2.10 = Creatininase; EC:4.1.1.17 = Ornithine decarboxylase; EC:4.3.1.12 = Ornithine cyclodeaminase. None of the enzymes enriched in this pathway in oral WT microbiome showed increased abundance in the gut microbiome of WT mice relative to APP/PS1 mice. Furthermore, a substantial number of these enzymes were completely undetected in the gut microbial ecosystem. Consequently, these enzymes were not considered from our most recent study examining the influence of the gut microbiome on Alzheimer's disease (AD).


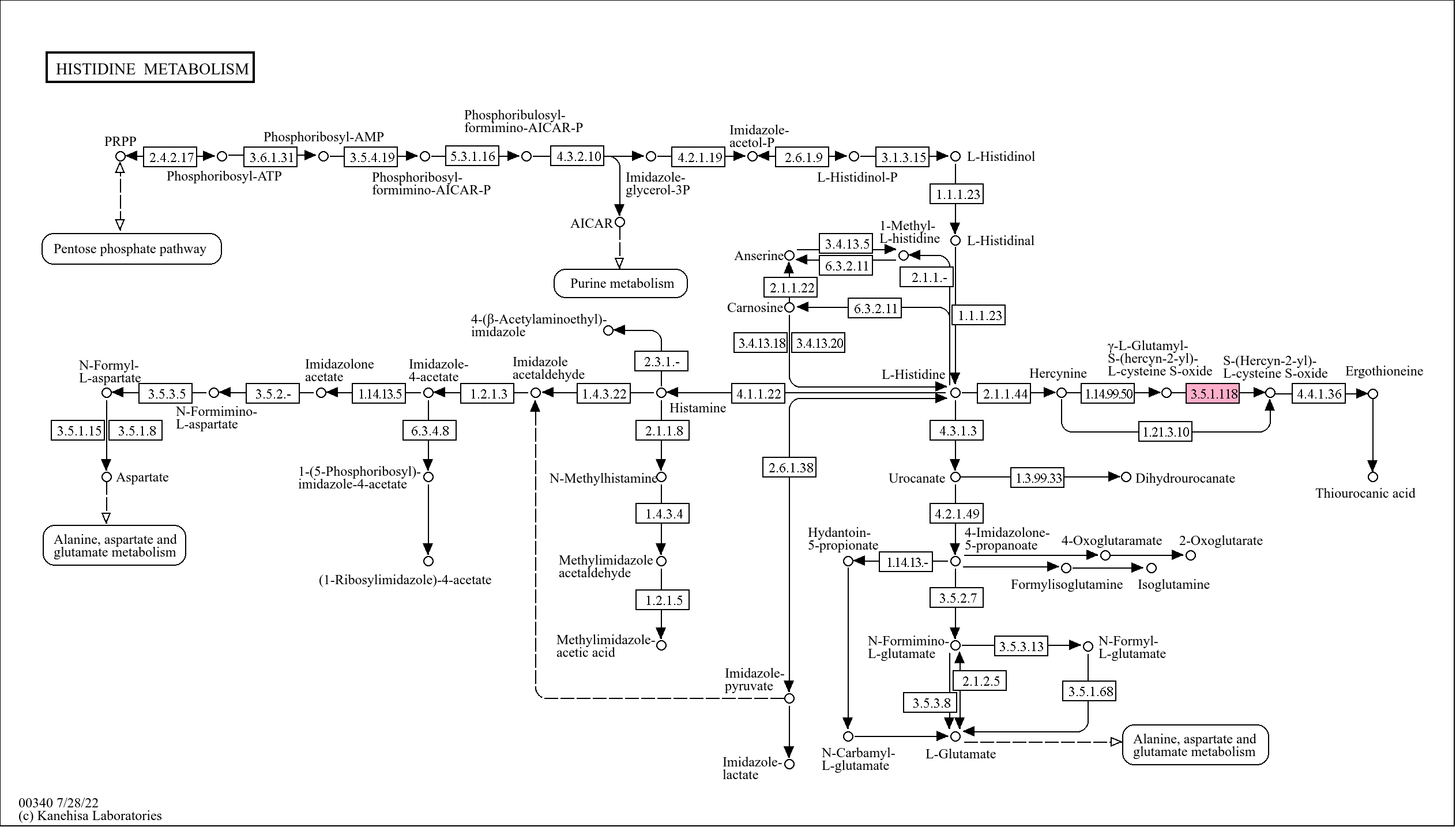
Fig. S26. Enzymatic profile of the "Histidine metabolism" KEGG pathway in the oral microbiome, exhibiting a ≥ 0.2-fold decrease in abundance in transgenic male littermates expressing amyloid precursor protein/presenilin-1 (APP/PS1) compared to age-matched wild-type controls. EC:3.5.1.118 = Gamma-glutamyl hercynylcysteine S-oxide hydrolase. None of the enzymes enriched in this pathway in oral WT microbiome showed increased abundance in the gut microbiome of WT mice relative to APP/PS1 mice. Furthermore, a substantial number of these enzymes were completely undetected in the gut microbial ecosystem. Consequently, these enzymes were not considered from our most recent study examining the influence of the gut microbiome on Alzheimer's disease (AD).


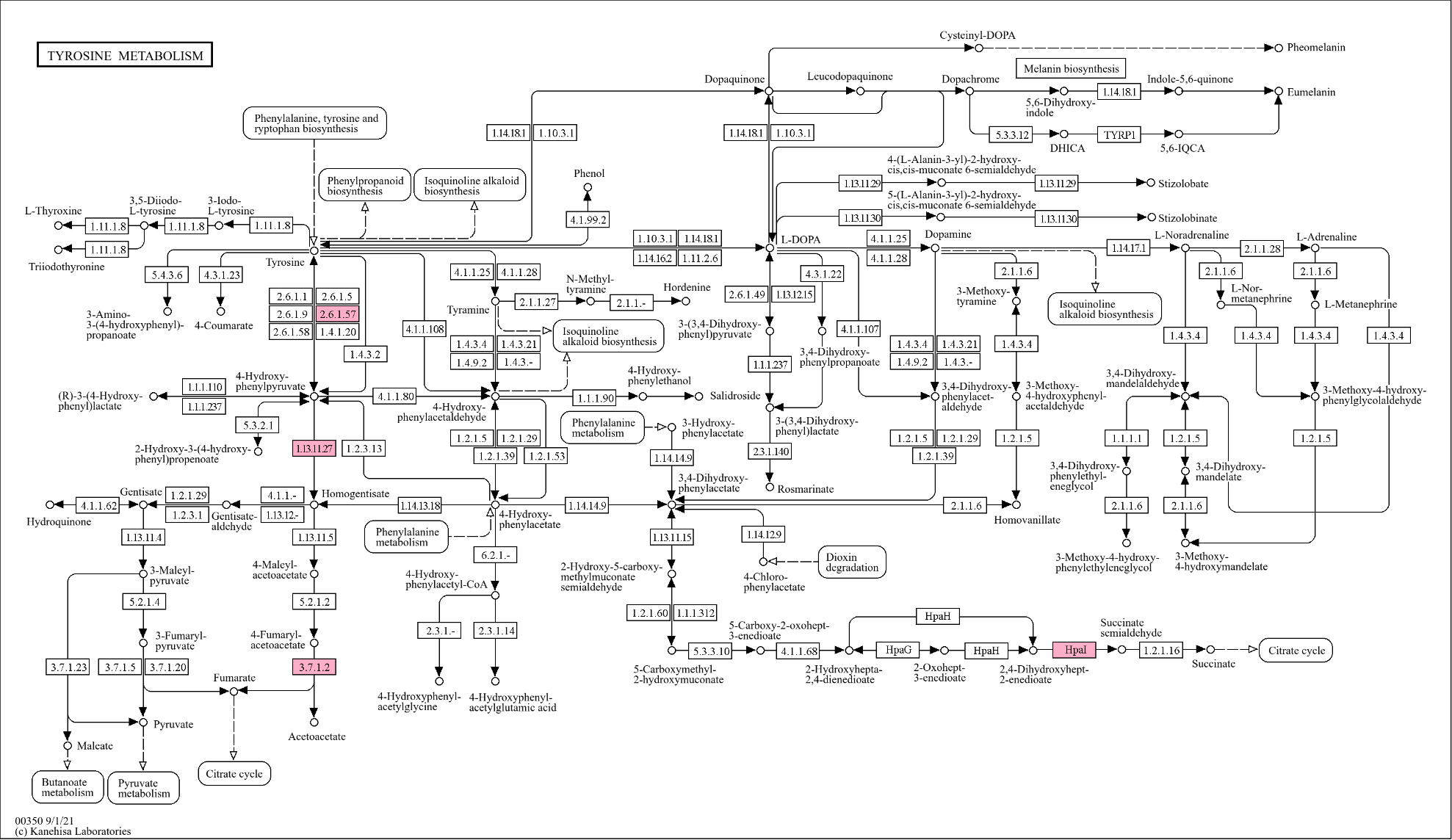
Fig. S27. Enzymatic profile of the "Tyrosine metabolism" KEGG pathway in the oral microbiome, exhibiting a ≥ 0.2-fold decrease in abundance in transgenic male littermates expressing amyloid precursor protein/presenilin-1 (APP/PS1) compared to age-matched wild-type controls. EC:1.13.11.27 = 4-hydroxyphenylpyruvate dioxygenase; EC:2.6.1.57 = Aromatic-amino-acid transaminase; EC:3.7.1.2 = Fumaryl acetoacetase; EC:4.1.2.52 = 4-hydroxy-2-oxoheptanedioate aldolase (HpaI). None of the enzymes enriched in this pathway in oral WT microbiome showed increased abundance in the gut microbiome of WT mice relative to APP/PS1 mice. Furthermore, a substantial number of these enzymes were completely undetected in the gut microbial ecosystem. Consequently, these enzymes were not considered from our most recent study examining the influence of the gut microbiome on Alzheimer's disease (AD).


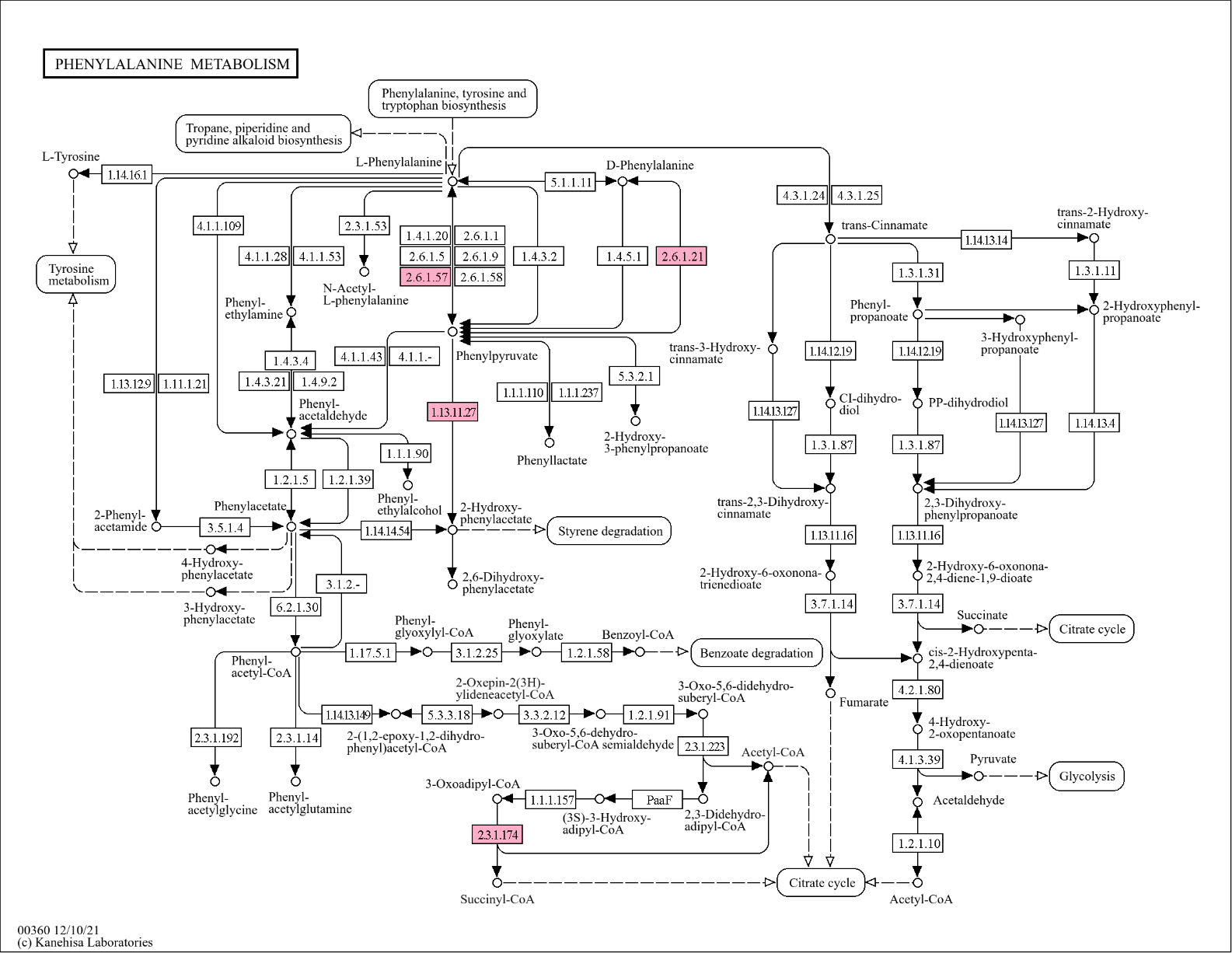
Fig. S28. Enzymatic profile of the "Phenylalanine metabolism" KEGG pathway in the oral microbiome, exhibiting a ≥ 0.2-fold decrease in abundance in transgenic male littermates expressing amyloid precursor protein/presenilin-1 (APP/PS1) compared to age-matched wild-type controls. EC:1.13.11.27 = 4-hydroxyphenylpyruvate dioxygenase; EC:2.6.1.21 = D-amino-acid transaminase; EC:2.6.1.57 = Aromatic-amino-acid transaminase; EC:1.1.1.174 = 3-Oxoadipyl-CoA thiolase. None of the enzymes enriched in this pathway in oral WT microbiome showed increased abundance in the gut microbiome of WT mice relative to APP/PS1 mice. Furthermore, a substantial number of these enzymes were completely undetected in the gut microbial ecosystem. Consequently, these enzymes were not considered from our most recent study examining the influence of the gut microbiome on Alzheimer's disease (AD).


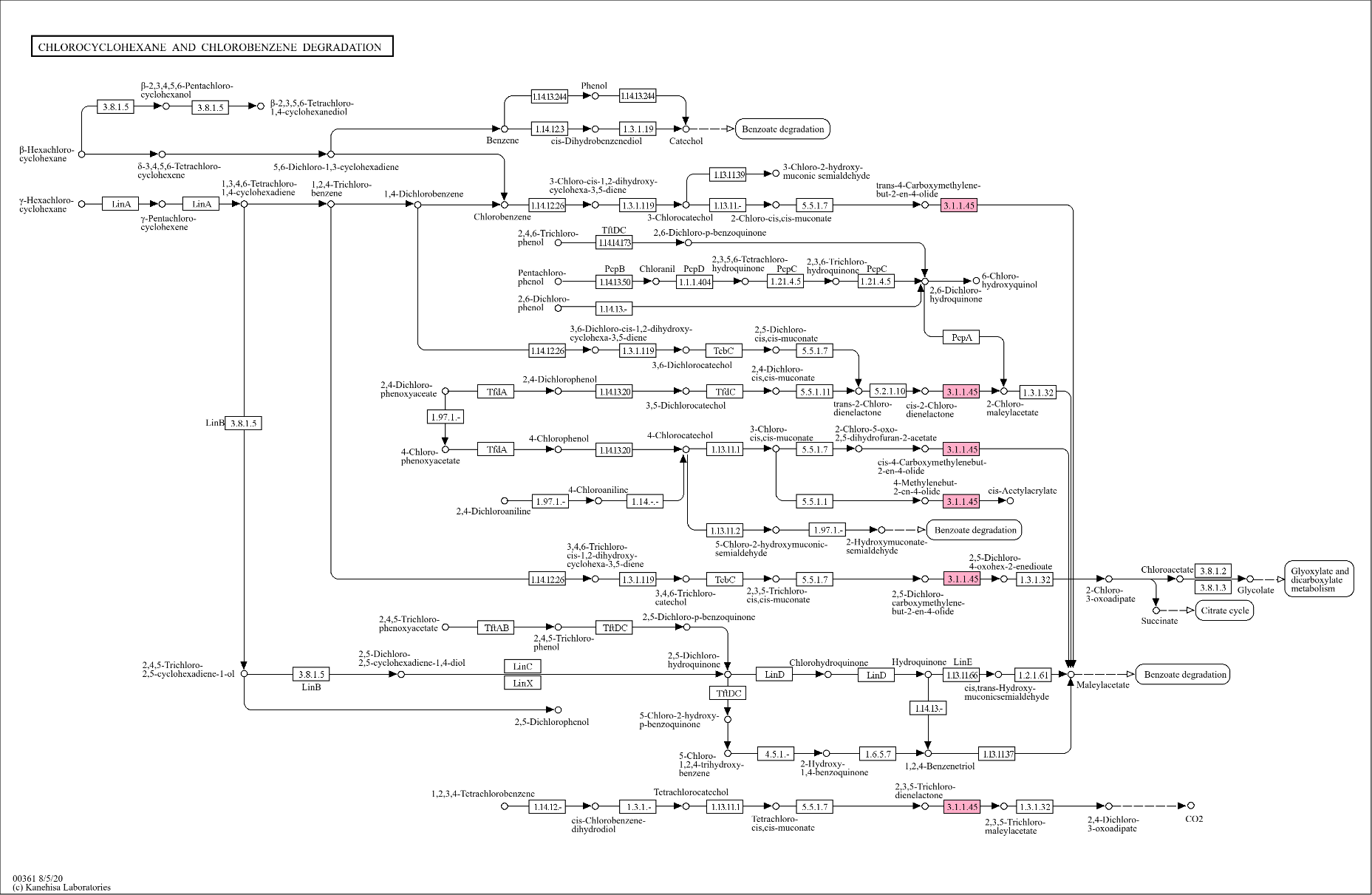
Fig. S29. Enzymatic profile of the "Chlorocyclohexane and chlorobenzene degradation" KEGG pathway in the oral microbiome, exhibiting a ≥ 0.2-fold decrease in abundance in transgenic male littermates expressing amyloid precursor protein/presenilin-1 (APP/PS1) compared to age-matched wild-type controls. EC:3.1.1.45 = Carboxymethylene butenolidase. None of the enzymes enriched in this pathway in oral WT microbiome showed increased abundance in the gut microbiome of WT mice relative to APP/PS1 mice. Furthermore, a substantial number of these enzymes were completely undetected in the gut microbial ecosystem. Consequently, these enzymes were not considered from our most recent study examining the influence of the gut microbiome on Alzheimer's disease (AD).


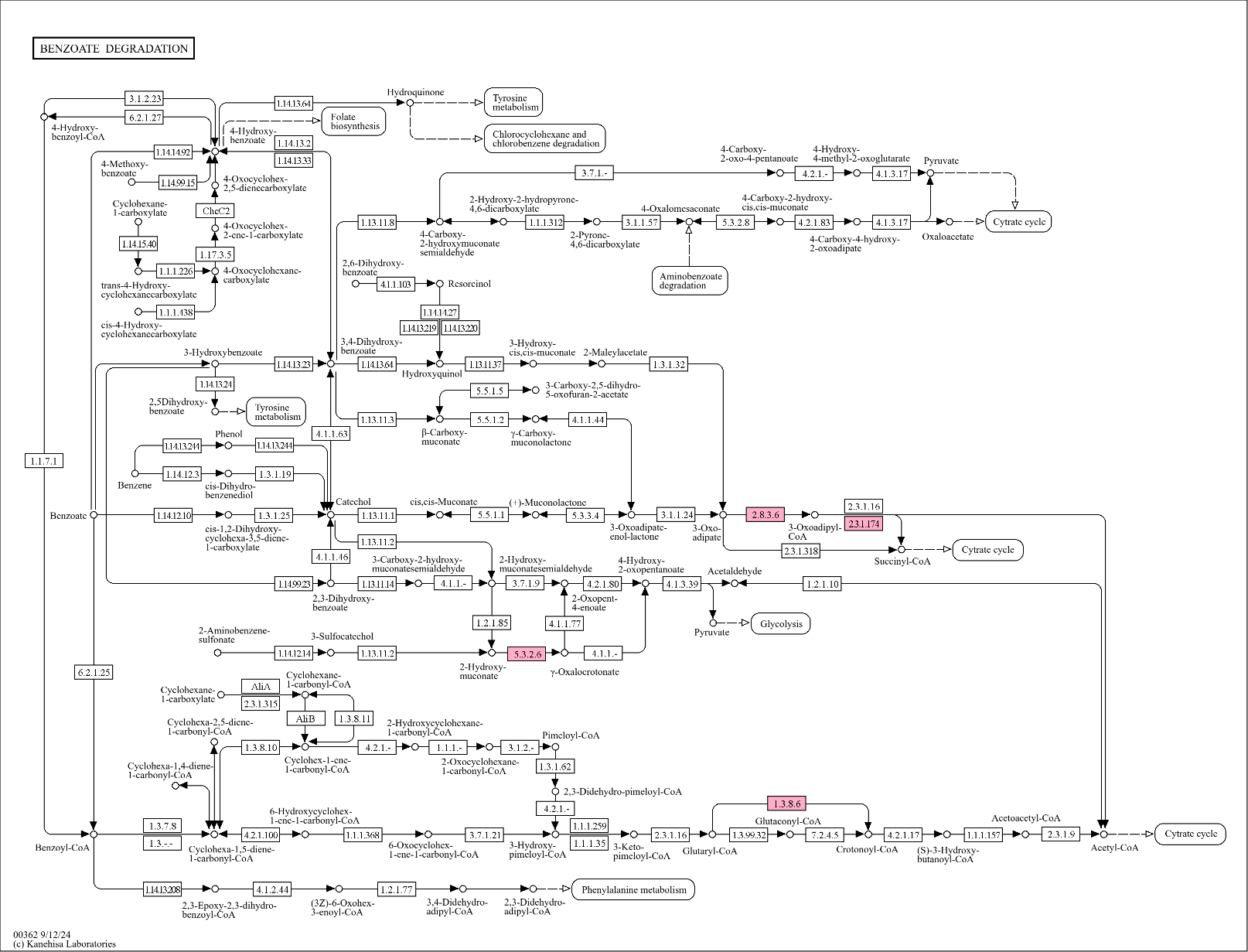
Fig. S30. Enzymatic profile of the "Benzoate degradation" KEGG pathway in the oral microbiome, exhibiting a ≥ 0.2-fold decrease in abundance in transgenic male littermates expressing amyloid precursor protein/presenilin-1 (APP/PS1) compared to age-matched wild-type controls. EC:1.3.8.6 = Glutaryl-CoA dehydrogenase (ETF); EC:2.8.3.6 = 3-Oxoadipate CoA-transferase; EC:5.3.2.6 = 2-hydroxymuconate tautomerase; EC:1.1.1.174 = 3-Oxoadipyl-CoA thiolase. None of the enzymes enriched in this pathway in oral WT microbiome showed increased abundance in the gut microbiome of WT mice relative to APP/PS1 mice. Furthermore, a substantial number of these enzymes were completely undetected in the gut microbial ecosystem. Consequently, these enzymes were not considered from our most recent study examining the influence of the gut microbiome on Alzheimer's disease (AD).


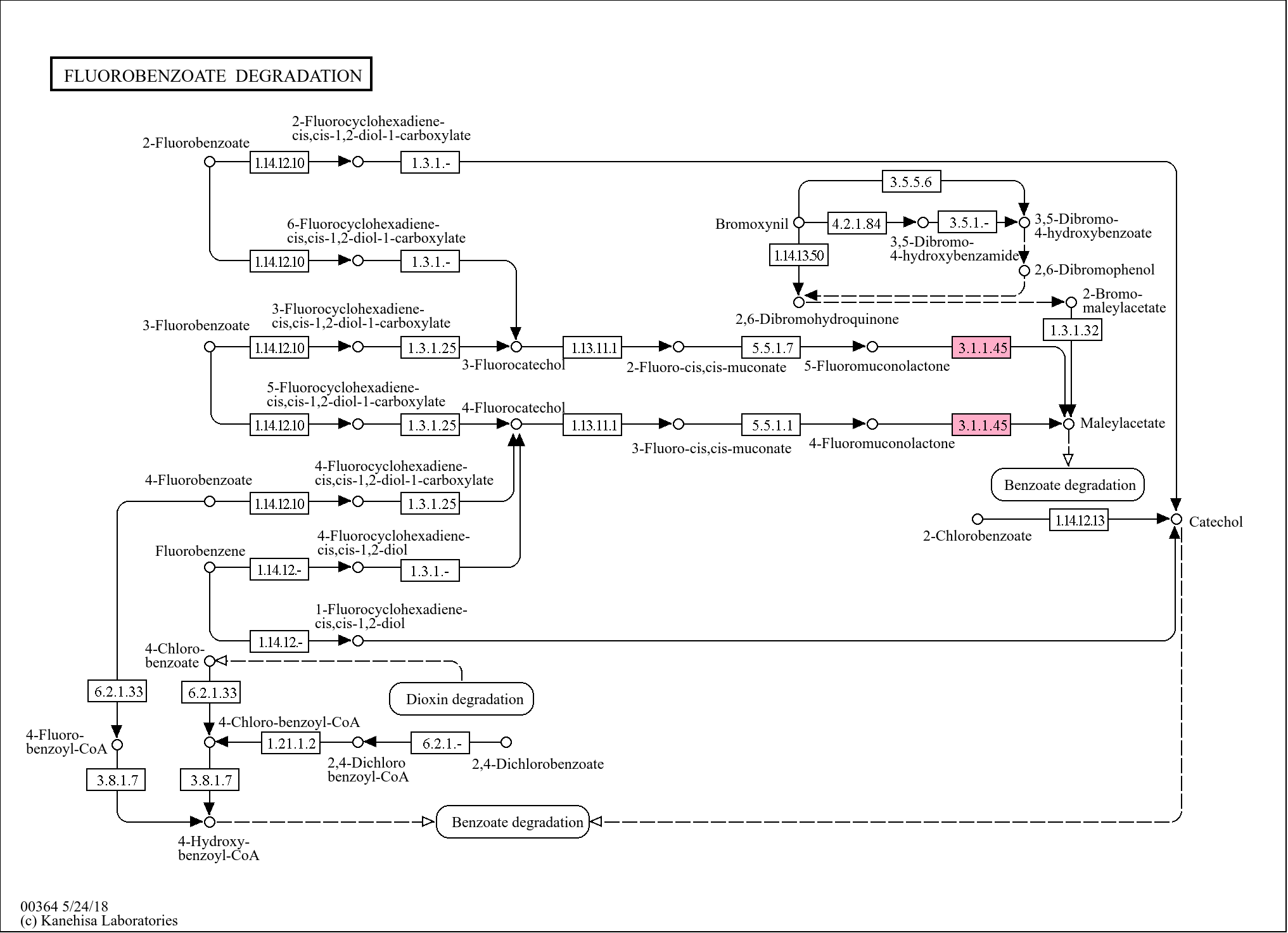
Fig. S31. Enzymatic profile of the "Fluorobenzoate degradation" KEGG pathway in the oral microbiome, exhibiting a ≥ 0.2-fold decrease in abundance in transgenic male littermates expressing amyloid precursor protein/presenilin-1 (APP/PS1) compared to age-matched wild-type controls. EC:3.1.1.45 = Carboxymethylene butenolidase. None of the enzymes enriched in this pathway in oral WT microbiome showed increased abundance in the gut microbiome of WT mice relative to APP/PS1 mice. Furthermore, a substantial number of these enzymes were completely undetected in the gut microbial ecosystem. Consequently, these enzymes were not considered from our most recent study examining the influence of the gut microbiome on Alzheimer's disease (AD).


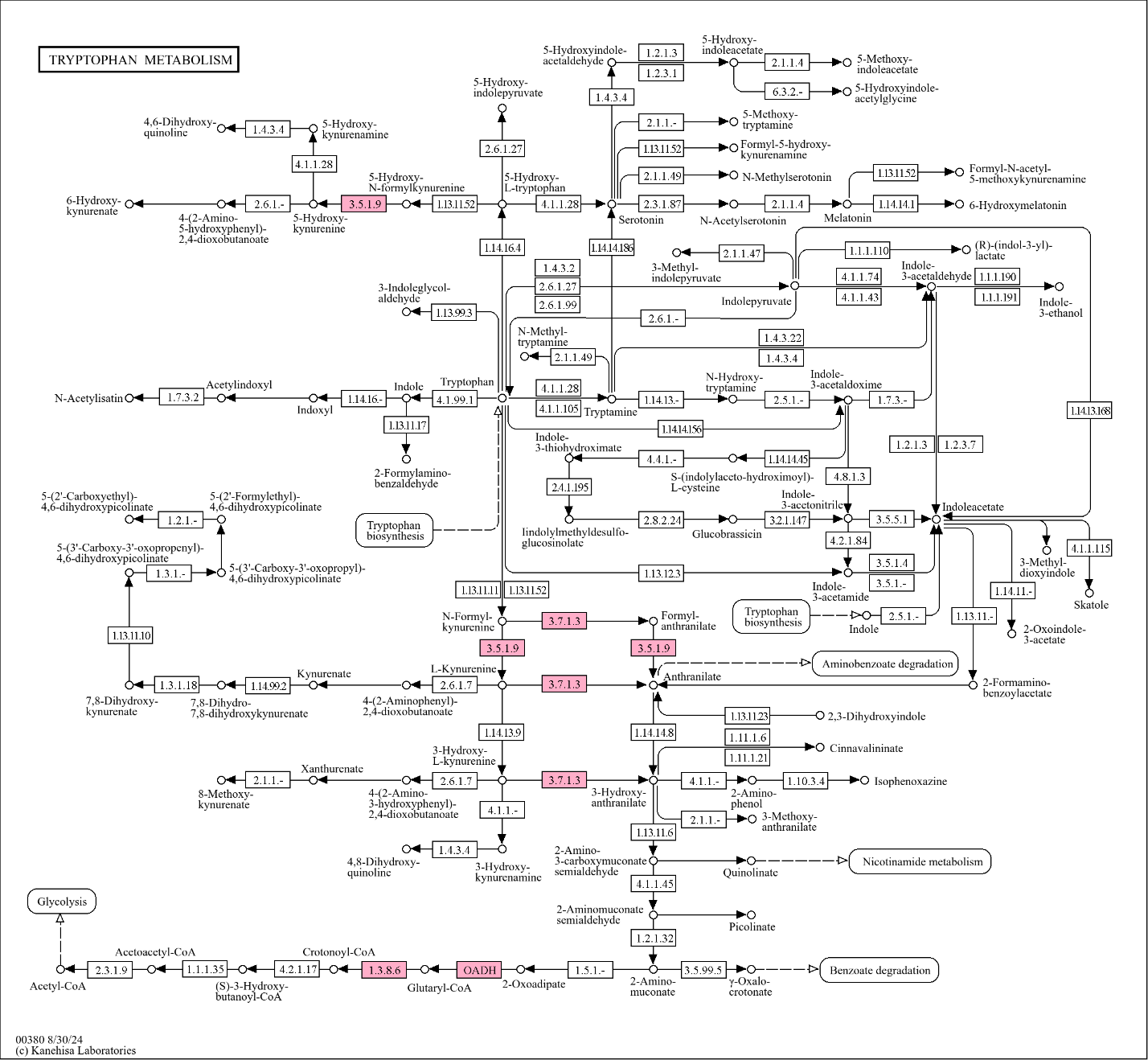
Fig. S32. Enzymatic profile of the "Tryptophan metabolism" KEGG pathway in the oral microbiome, exhibiting a ≥ 0.2-fold decrease in abundance in transgenic male littermates expressing amyloid precursor protein/presenilin-1 (APP/PS1) compared to age-matched wild-type controls. EC:1.3.8.6 = Glutaryl-CoA dehydrogenase (ETF); EC:2.3.1.61 = Dihydrolipoyllysine-residue succinyltransferase (OADH); EC:3.5.1.9 = Arylformamidase; EC:3.7.1.3 = Kynureninase. None of the enzymes enriched in this pathway in oral WT microbiome showed increased abundance in the gut microbiome of WT mice relative to APP/PS1 mice. Furthermore, a substantial number of these enzymes were completely undetected in the gut microbial ecosystem. Consequently, these enzymes were not considered from our most recent study examining the influence of the gut microbiome on Alzheimer's disease (AD).


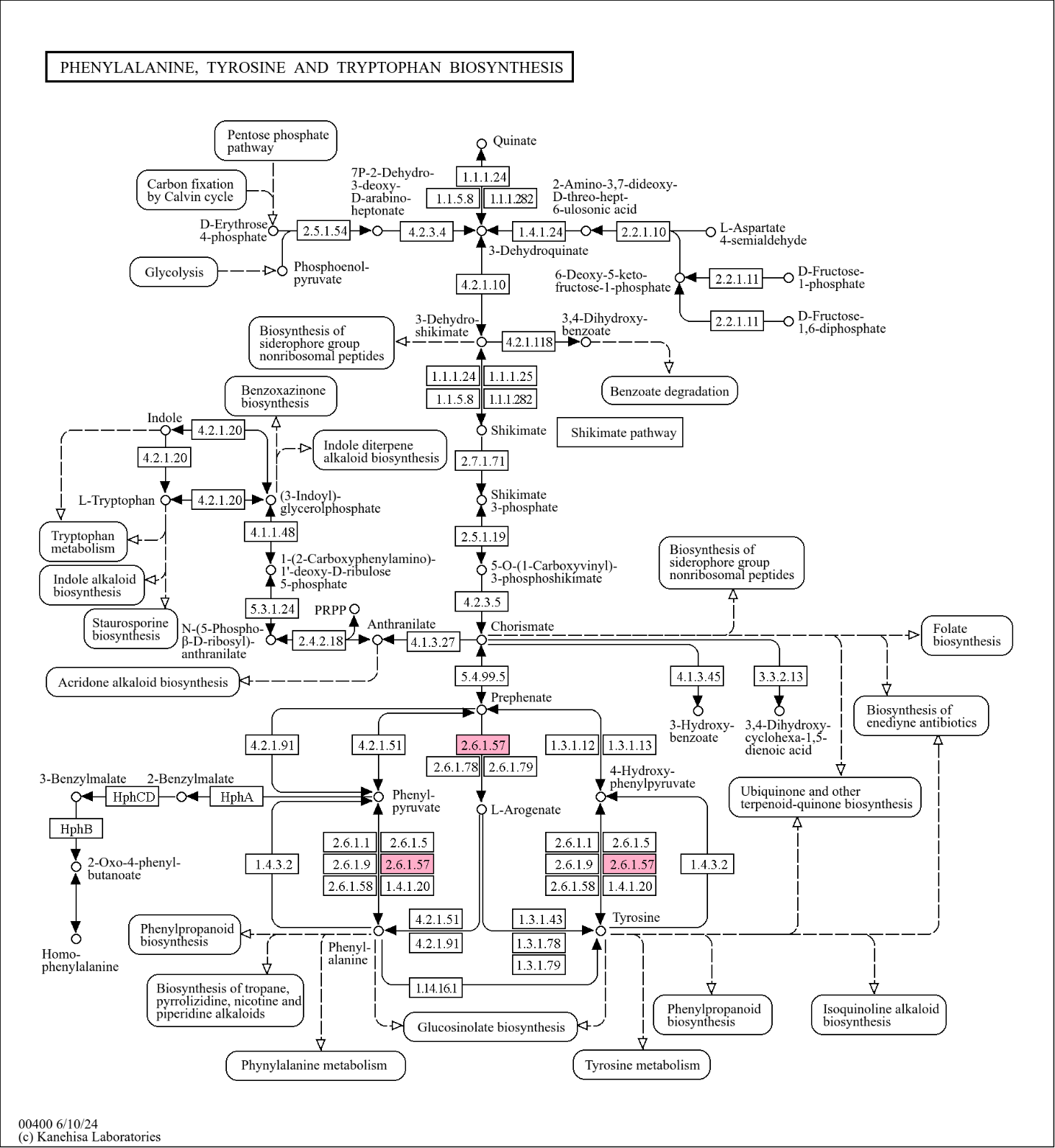
Fig. S33. Enzymatic profile of the "Phenylalanine, tyrosine and tryptophan biosynthesis" KEGG pathway in the oral microbiome, exhibiting a ≥ 0.2-fold decrease in abundance in transgenic male littermates expressing amyloid precursor protein/presenilin-1 (APP/PS1) compared to age-matched wild-type controls. EC:2.6.1.57 = Aromatic-amino-acid transaminase. None of the enzymes enriched in this pathway in oral WT microbiome showed increased abundance in the gut microbiome of WT mice relative to APP/PS1 mice. Furthermore, a substantial number of these enzymes were completely undetected in the gut microbial ecosystem. Consequently, these enzymes were not considered from our most recent study examining the influence of the gut microbiome on Alzheimer's disease (AD).


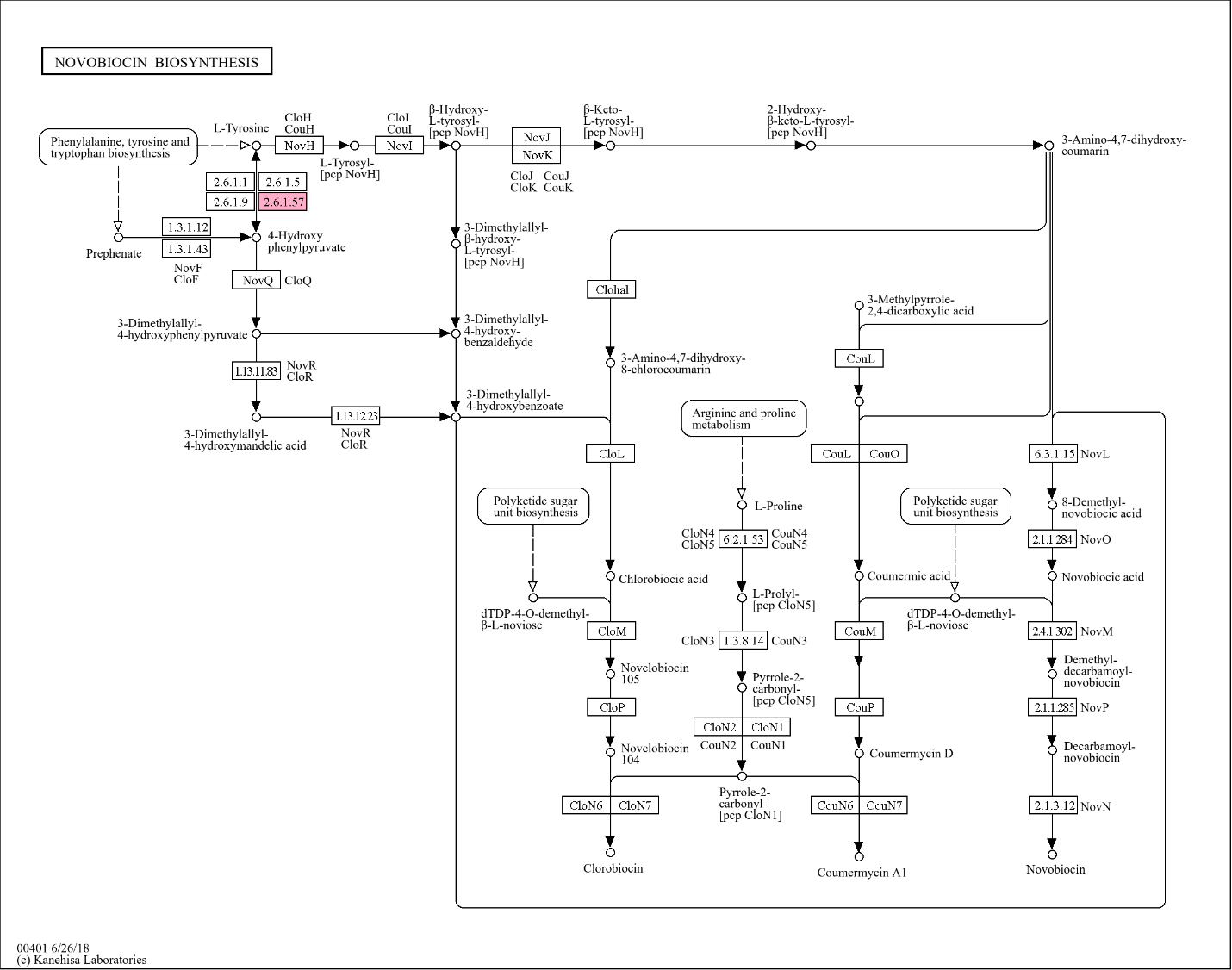
Fig. S34. Enzymatic profile of the "Novobiocin biosynthesis" KEGG pathway in the oral microbiome, exhibiting a ≥ 0.2-fold decrease in abundance in transgenic male littermates expressing amyloid precursor protein/presenilin-1 (APP/PS1) compared to age-matched wild-type controls. EC:2.6.1.57 = Aromatic-amino-acid transaminase. None of the enzymes enriched in this pathway in oral WT microbiome showed increased abundance in the gut microbiome of WT mice relative to APP/PS1 mice. Furthermore, a substantial number of these enzymes were completely undetected in the gut microbial ecosystem. Consequently, these enzymes were not considered from our most recent study examining the influence of the gut microbiome on Alzheimer's disease (AD).


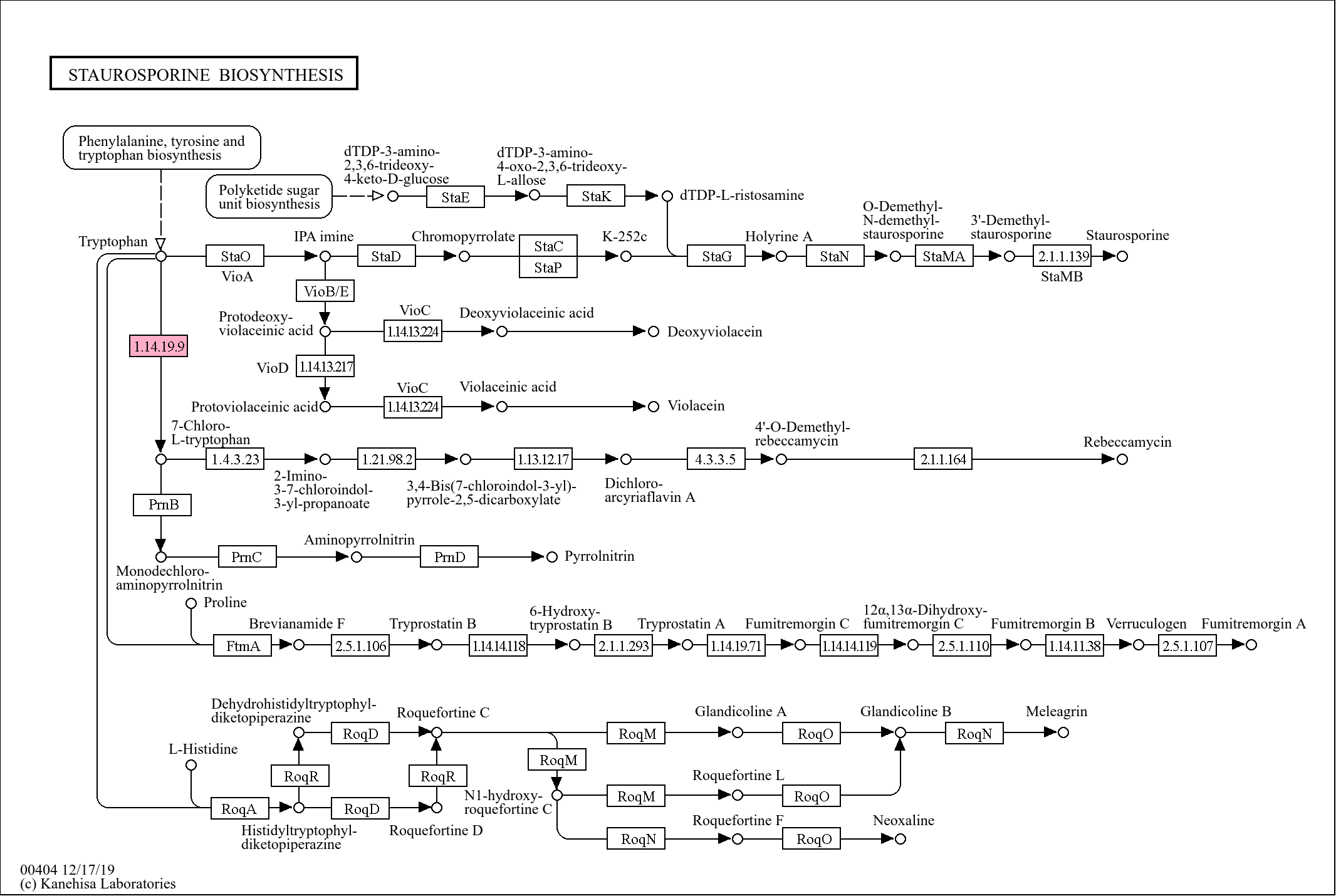
Fig. S35. Enzymatic profile of the "Staurosporine biosynthesis" KEGG pathway in the oral microbiome, exhibiting a ≥ 0.2-fold decrease in abundance in transgenic male littermates expressing amyloid precursor protein/presenilin-1 (APP/PS1) compared to age-matched wild-type controls. EC:1.14.19.9 = Tryptophan 7-halogenase. None of the enzymes enriched in this pathway in oral WT microbiome showed increased abundance in the gut microbiome of WT mice relative to APP/PS1 mice. Furthermore, a substantial number of these enzymes were completely undetected in the gut microbial ecosystem. Consequently, these enzymes were not considered from our most recent study examining the influence of the gut microbiome on Alzheimer's disease (AD).


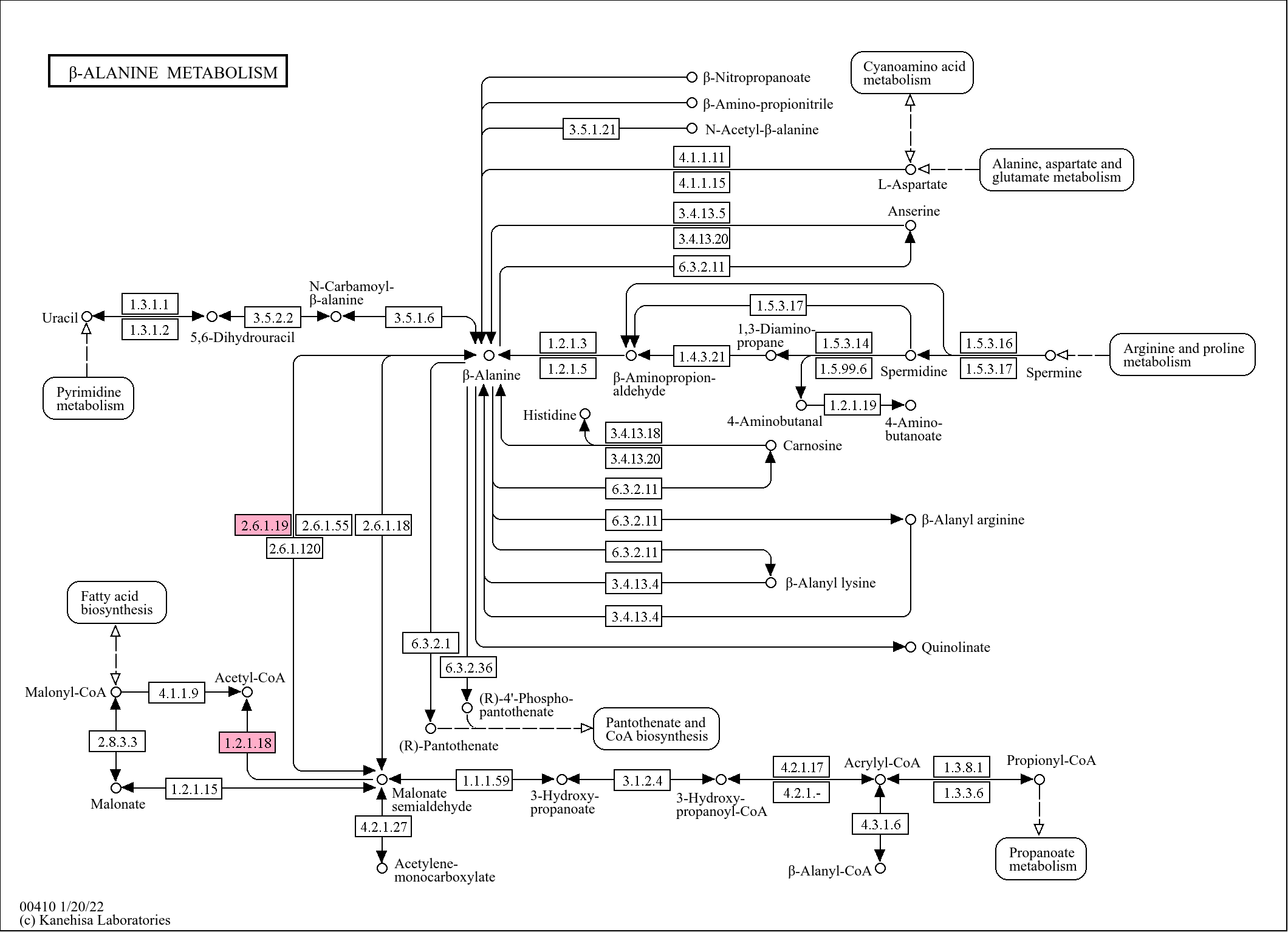
Fig. S36. Enzymatic profile of the "β-Alanine metabolism" KEGG pathway in the oral microbiome, exhibiting a ≥ 0.2-fold decrease in abundance in transgenic male littermates expressing amyloid precursor protein/presenilin-1 (APP/PS1) compared to age-matched wild-type controls. EC:1.2.1.18 = Malonate-semialdehyde dehydrogenase (acetylating); EC:2.6.1.19 = 4-aminobutyrate--2-oxoglutarate transaminase. None of the enzymes enriched in this pathway in oral WT microbiome showed increased abundance in the gut microbiome of WT mice relative to APP/PS1 mice. Furthermore, a substantial number of these enzymes were completely undetected in the gut microbial ecosystem. Consequently, these enzymes were not considered from our most recent study examining the influence of the gut microbiome on Alzheimer's disease (AD).


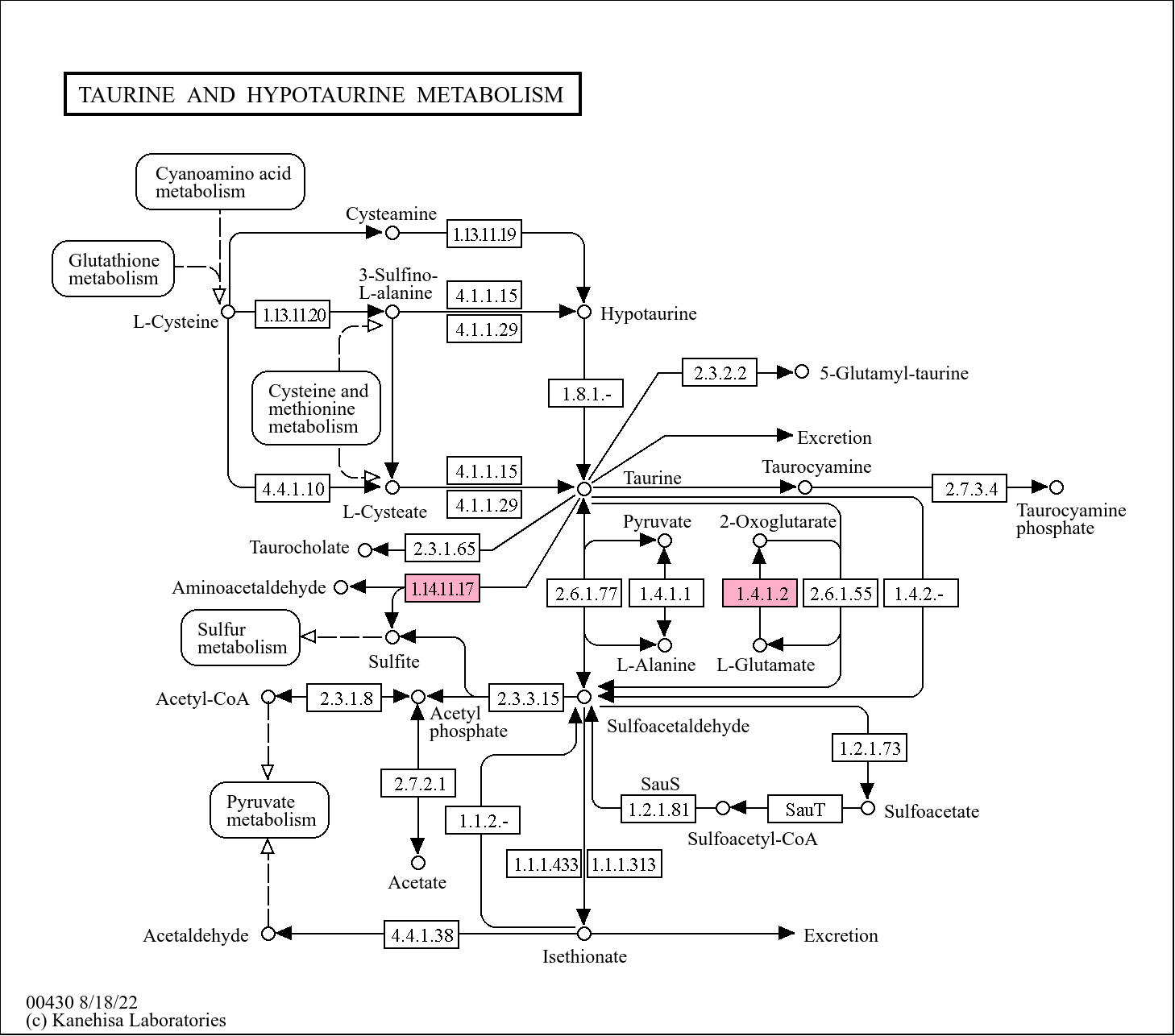
Fig. S37. Enzymatic profile of the "Taurine and hypotaurine metabolism" KEGG pathway in the oral microbiome, exhibiting a ≥ 0.2-fold decrease in abundance in transgenic male littermates expressing amyloid precursor protein/presenilin-1 (APP/PS1) compared to age-matched wild-type controls. EC:1.14.11.17 = Taurine dioxygenase; *EC:1.4.1.2 = Glutamate dehydrogenase. The red asterisk (*) symbol functions as a crucial visual marker, highlighting specific KEGG enzymes that have been either substantiated through scientific inquiry or are hypothesized to potentially play pivotal roles in the onset or progression of Alzheimer’s disease (AD). None of the enzymes enriched in this pathway in oral WT microbiome showed increased abundance in the gut microbiome of WT mice relative to APP/PS1 mice. Furthermore, a substantial number of these enzymes were completely undetected in the gut microbial ecosystem. Consequently, these enzymes were not considered from our most recent study examining the influence of the gut microbiome on Alzheimer's disease (AD).


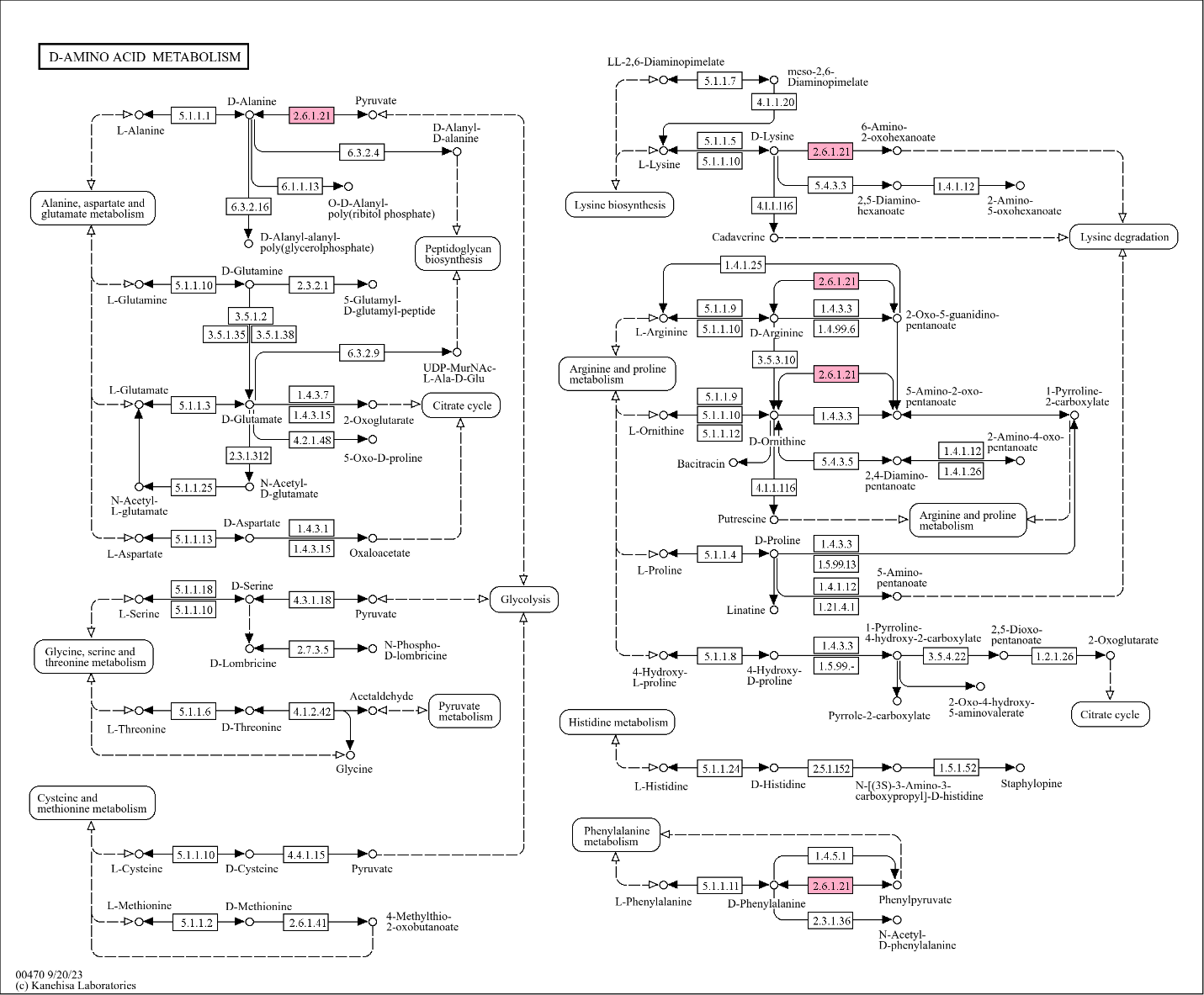
Fig. S38. Enzymatic profile of the "D-Amino acid metabolism" KEGG pathway in the oral microbiome, exhibiting a ≥ 0.2-fold decrease in abundance in transgenic male littermates expressing amyloid precursor protein/presenilin-1 (APP/PS1) compared to age-matched wild-type controls. EC:2.6.1.21 = D-amino-acid transaminase. None of the enzymes enriched in this pathway in oral WT microbiome showed increased abundance in the gut microbiome of WT mice relative to APP/PS1 mice. Furthermore, a substantial number of these enzymes were completely undetected in the gut microbial ecosystem. Consequently, these enzymes were not considered from our most recent study examining the influence of the gut microbiome on Alzheimer's disease (AD).


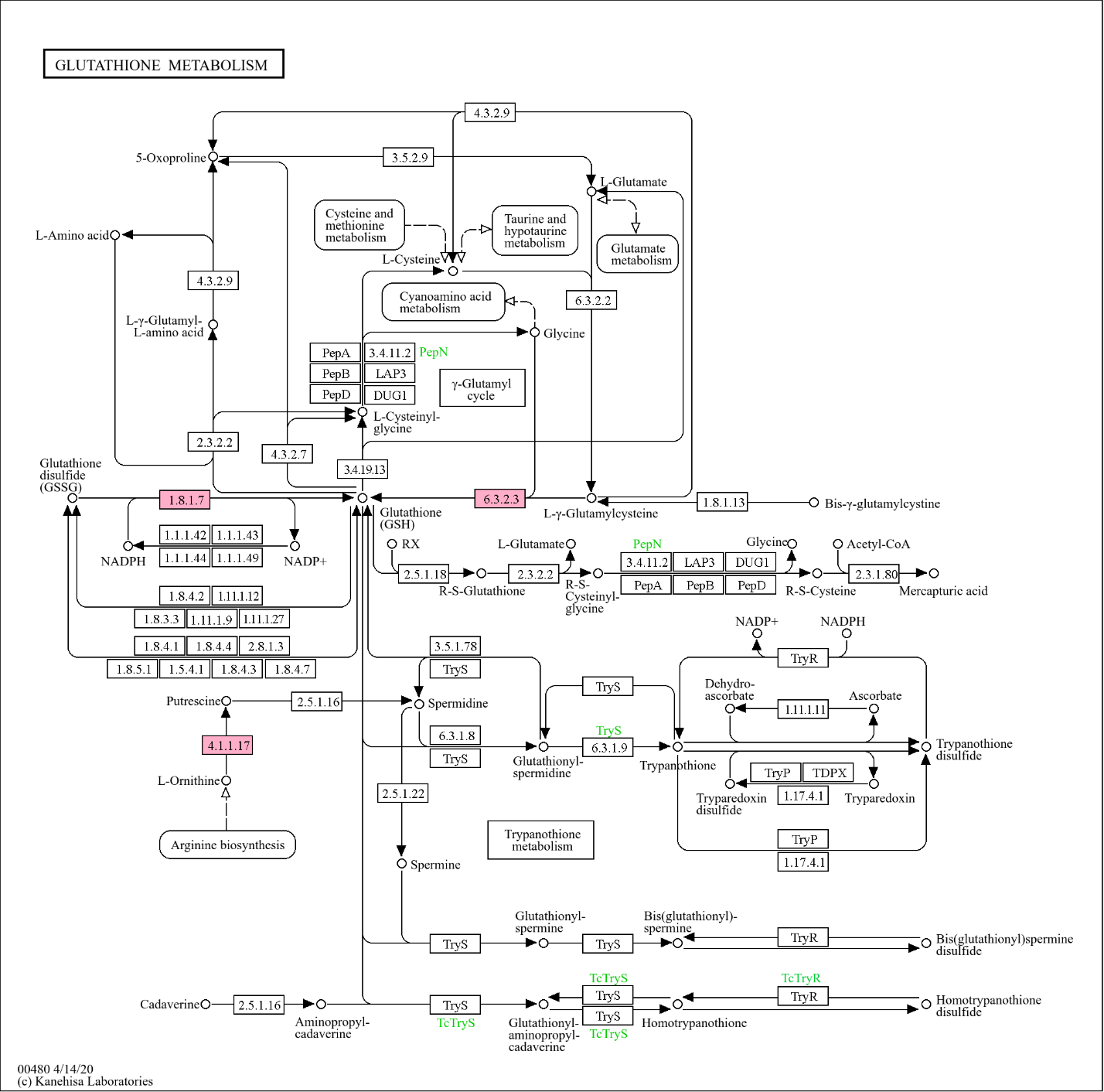
Fig. S39. Enzymatic profile of the "Glutathione metabolism" KEGG pathway in the oral microbiome, exhibiting a ≥ 0.2-fold decrease in abundance in transgenic male littermates expressing amyloid precursor protein/presenilin-1 (APP/PS1) compared to age-matched wild-type controls. EC:4.1.1.17 = Ornithine decarboxylase; EC:6.3.2.3 = Glutathione synthase; *EC:1.8.1.7 = Glutathione-disulfide reductase. The red asterisk (*) symbol functions as a crucial visual marker, highlighting specific KEGG enzymes that have been either substantiated through scientific inquiry or are hypothesized to potentially play pivotal roles in the onset or progression of Alzheimer’s disease (AD). None of the enzymes enriched in this pathway in oral WT microbiome showed increased abundance in the gut microbiome of WT mice relative to APP/PS1 mice. Furthermore, a substantial number of these enzymes were completely undetected in the gut microbial ecosystem. Consequently, these enzymes were not considered from our most recent study examining the influence of the gut microbiome on Alzheimer's disease (AD).


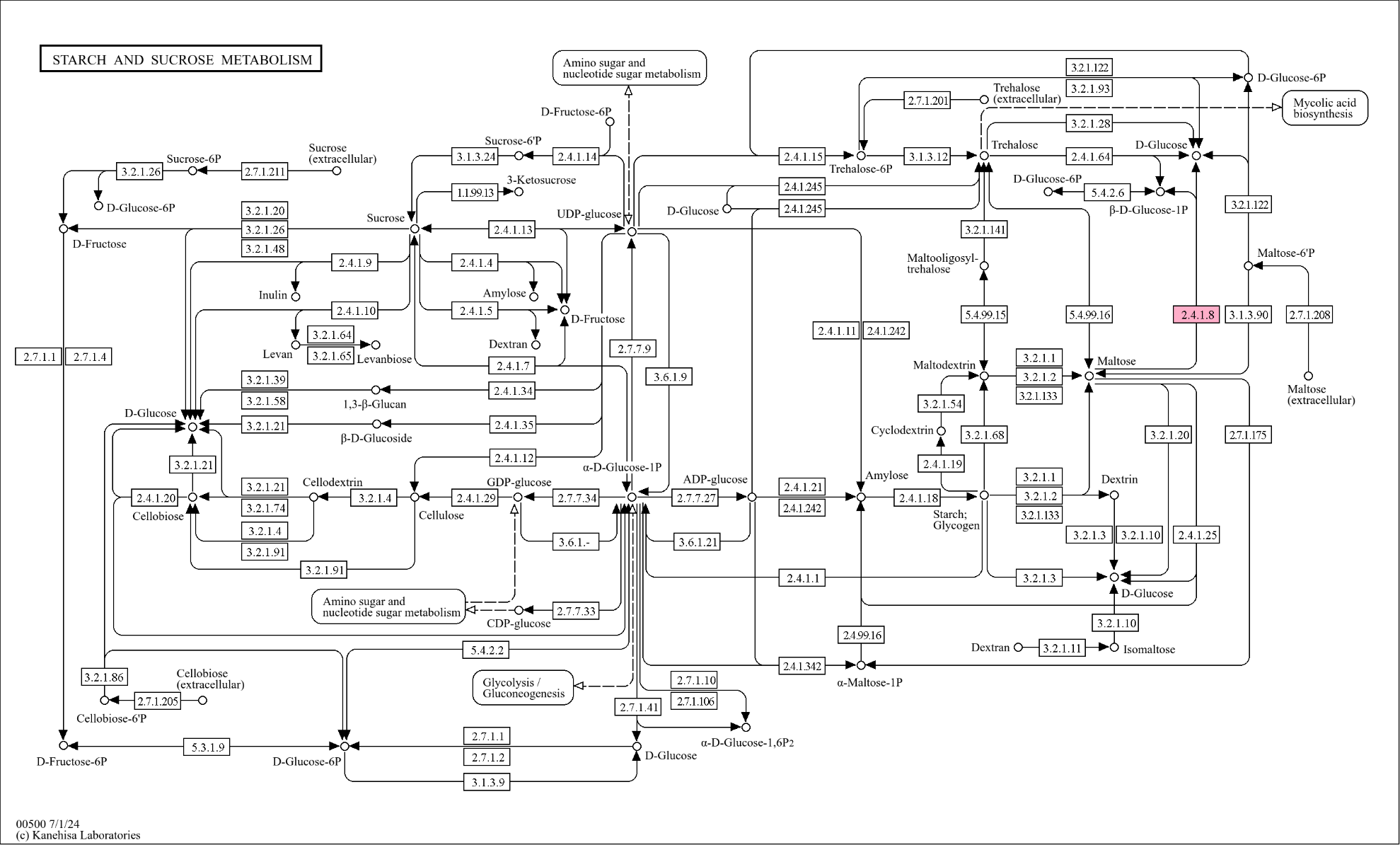
Fig. S40. Enzymatic profile of the "Starch and sucrose metabolism" KEGG pathway in the oral microbiome, exhibiting a ≥ 0.2-fold decrease in abundance in transgenic male littermates expressing amyloid precursor protein/presenilin-1 (APP/PS1) compared to age-matched wild-type controls. EC:2.4.1.8 = Maltose phosphorylase. None of the enzymes enriched in this pathway in oral WT microbiome showed increased abundance in the gut microbiome of WT mice relative to APP/PS1 mice. Furthermore, a substantial number of these enzymes were completely undetected in the gut microbial ecosystem. Consequently, these enzymes were not considered from our most recent study examining the influence of the gut microbiome on Alzheimer's disease (AD).


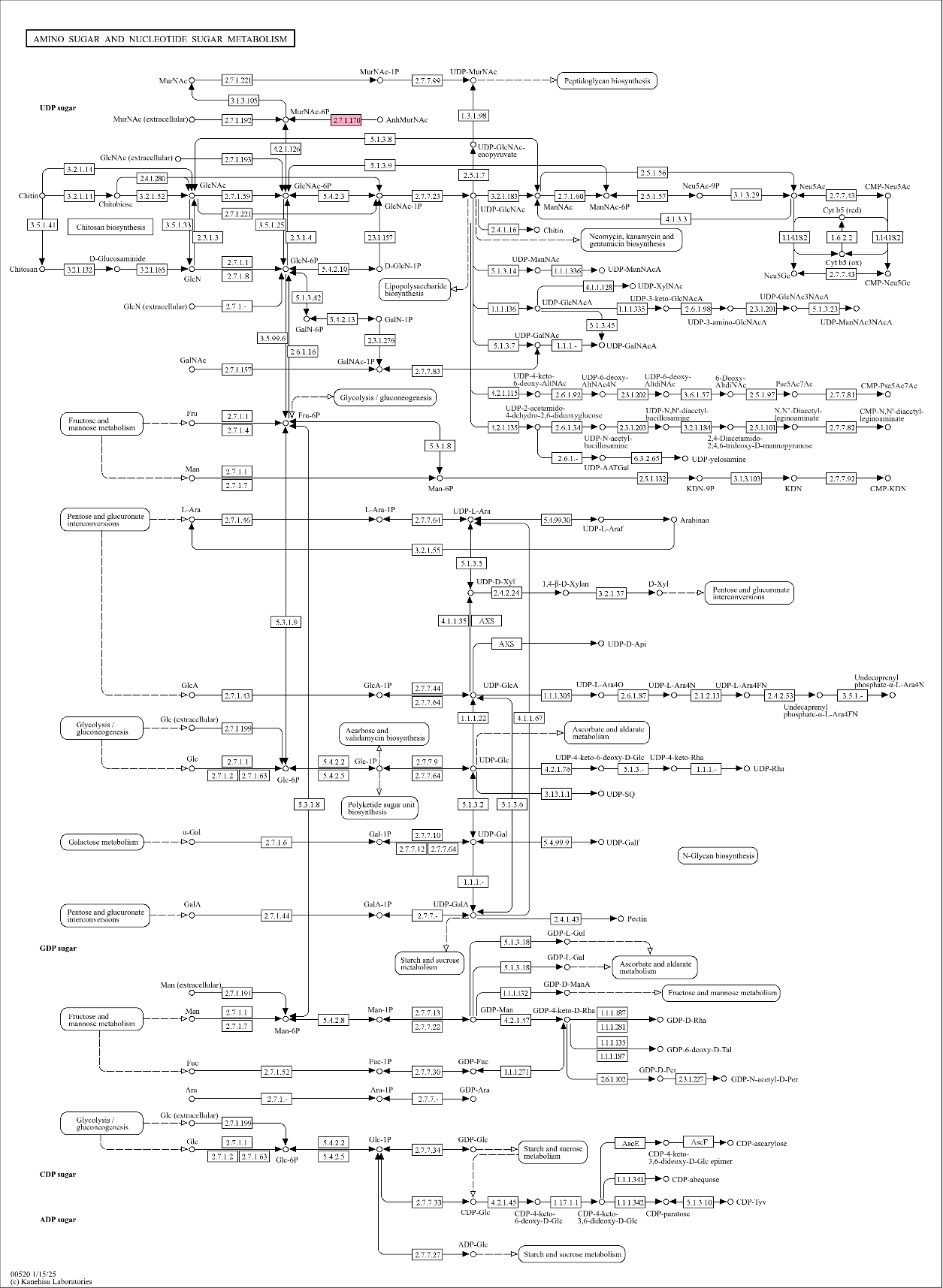
Fig. S41. Enzymatic profile of the "Amino sugar and nucleotide sugar metabolism" KEGG pathway in the oral microbiome, exhibiting a ≥ 0.2-fold decrease in abundance in transgenic male littermates expressing amyloid precursor protein/presenilin-1 (APP/PS1) compared to age-matched wild-type controls. EC:2.7.1.170 = Anhydro-N-acetylmuramic acid kinase. None of the enzymes enriched in this pathway in oral WT microbiome showed increased abundance in the gut microbiome of WT mice relative to APP/PS1 mice. Furthermore, a substantial number of these enzymes were completely undetected in the gut microbial ecosystem. Consequently, these enzymes were not considered from our most recent study examining the influence of the gut microbiome on Alzheimer's disease (AD).


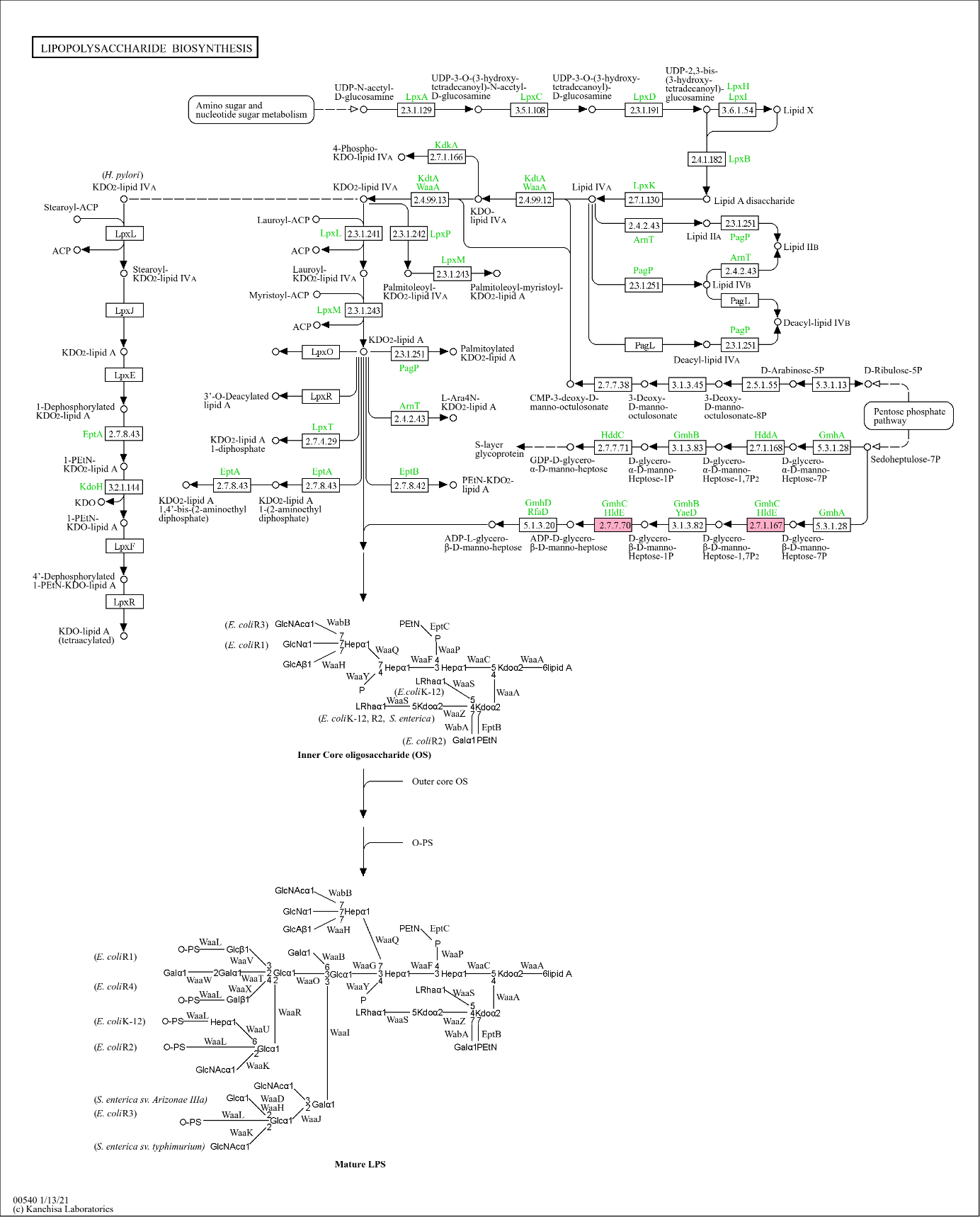
Fig. S42. Enzymatic profile of the "Lipopolysaccharide biosynthesis" KEGG pathway in the oral microbiome, exhibiting a ≥ 0.2-fold decrease in abundance in transgenic male littermates expressing amyloid precursor protein/presenilin-1 (APP/PS1) compared to age-matched wild-type controls. EC:2.7.1.167 = D-glycero-beta-D-manno-heptose-7-phosphate kinase; EC:2.7.7.70 = D-glycero-beta-D-manno-heptose 1-phosphate adenylyltransferase. None of the enzymes enriched in this pathway in oral WT microbiome showed increased abundance in the gut microbiome of WT mice relative to APP/PS1 mice. Furthermore, a substantial number of these enzymes were completely undetected in the gut microbial ecosystem. Consequently, these enzymes were not considered from our most recent study examining the influence of the gut microbiome on Alzheimer's disease (AD).


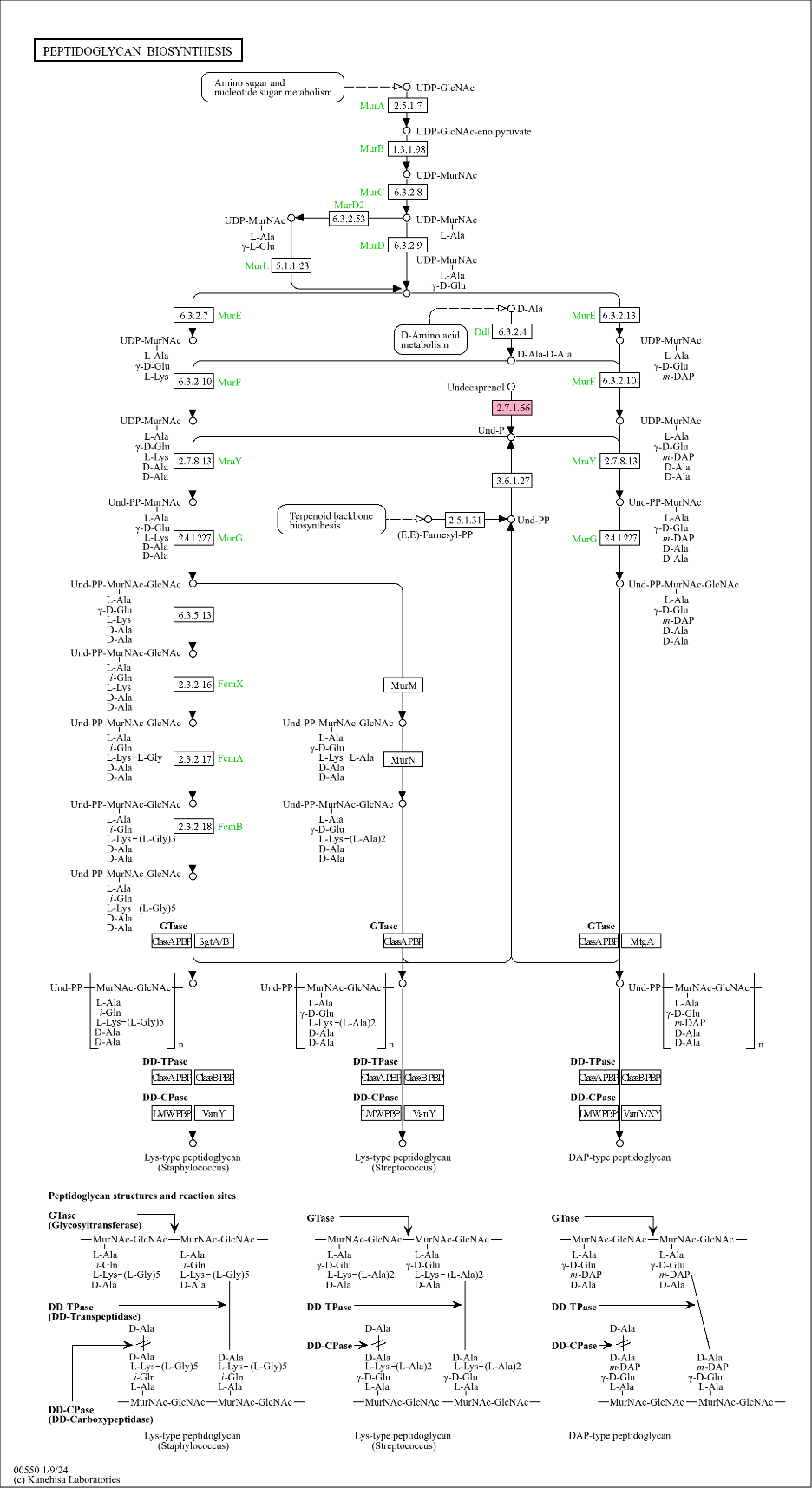
Fig. S43. Enzymatic profile of the "Peptidoglycan biosynthesis" KEGG pathway in the oral microbiome, exhibiting a ≥ 0.2-fold decrease in abundance in transgenic male littermates expressing amyloid precursor protein/presenilin-1 (APP/PS1) compared to age-matched wild-type controls. EC:2.7.1.66 = Undecaprenol kinase. None of the enzymes enriched in this pathway in oral WT microbiome showed increased abundance in the gut microbiome of WT mice relative to APP/PS1 mice. Furthermore, a substantial number of these enzymes were completely undetected in the gut microbial ecosystem. Consequently, these enzymes were not considered from our most recent study examining the influence of the gut microbiome on Alzheimer's disease (AD).


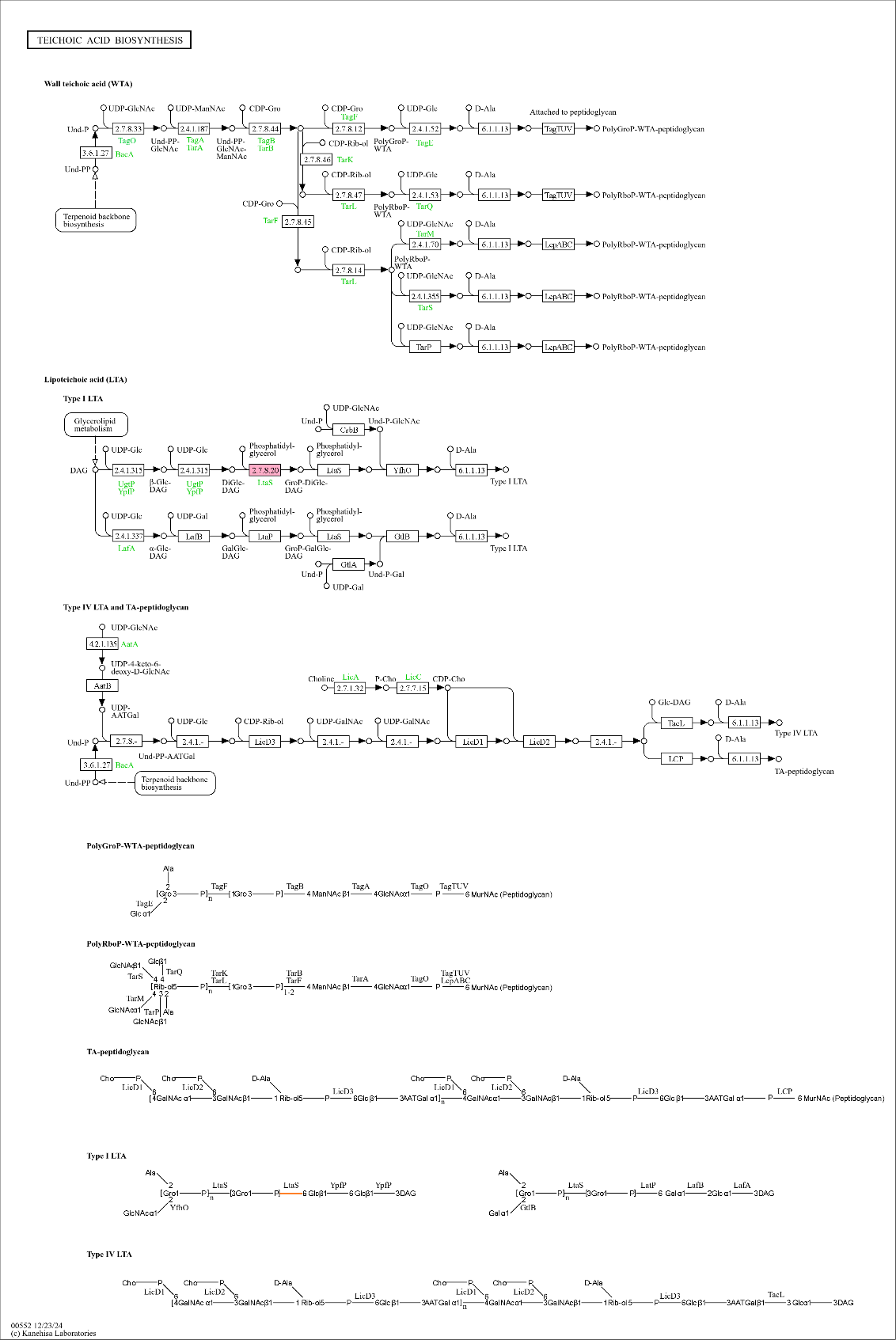
Fig. S44. Enzymatic profile of the "Teichoic acid biosynthesis" KEGG pathway in the oral microbiome, exhibiting a ≥ 0.2-fold decrease in abundance in transgenic male littermates expressing amyloid precursor protein/presenilin-1 (APP/PS1) compared to age-matched wild-type controls. EC:2.7.8.20 = Phosphatidylglycerol--membrane-oligosaccharide glycerophosphotransferase. None of the enzymes enriched in this pathway in oral WT microbiome showed increased abundance in the gut microbiome of WT mice relative to APP/PS1 mice. Furthermore, a substantial number of these enzymes were completely undetected in the gut microbial ecosystem. Consequently, these enzymes were not considered from our most recent study examining the influence of the gut microbiome on Alzheimer's disease (AD).


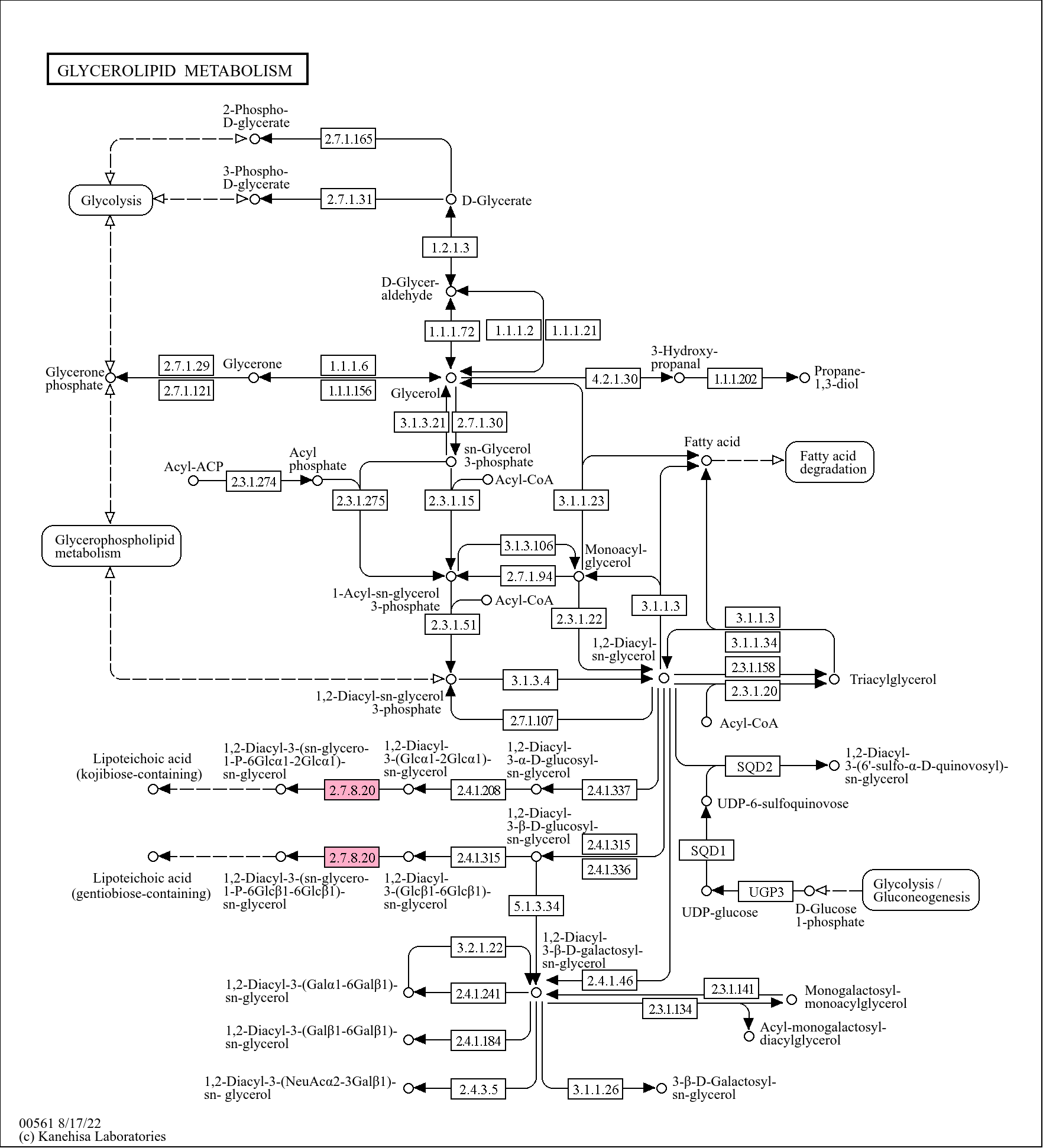
Fig. S45. Enzymatic profile of the "Glycerolipid metabolism" KEGG pathway in the oral microbiome, exhibiting a ≥ 0.2-fold decrease in abundance in transgenic male littermates expressing amyloid precursor protein/presenilin-1 (APP/PS1) compared to age-matched wild-type controls. EC:2.7.8.20 = Phosphatidylglycerol--membrane-oligosaccharide glycerophosphotransferase. None of the enzymes enriched in this pathway in oral WT microbiome showed increased abundance in the gut microbiome of WT mice relative to APP/PS1 mice. Furthermore, a substantial number of these enzymes were completely undetected in the gut microbial ecosystem. Consequently, these enzymes were not considered from our most recent study examining the influence of the gut microbiome on Alzheimer's disease (AD).


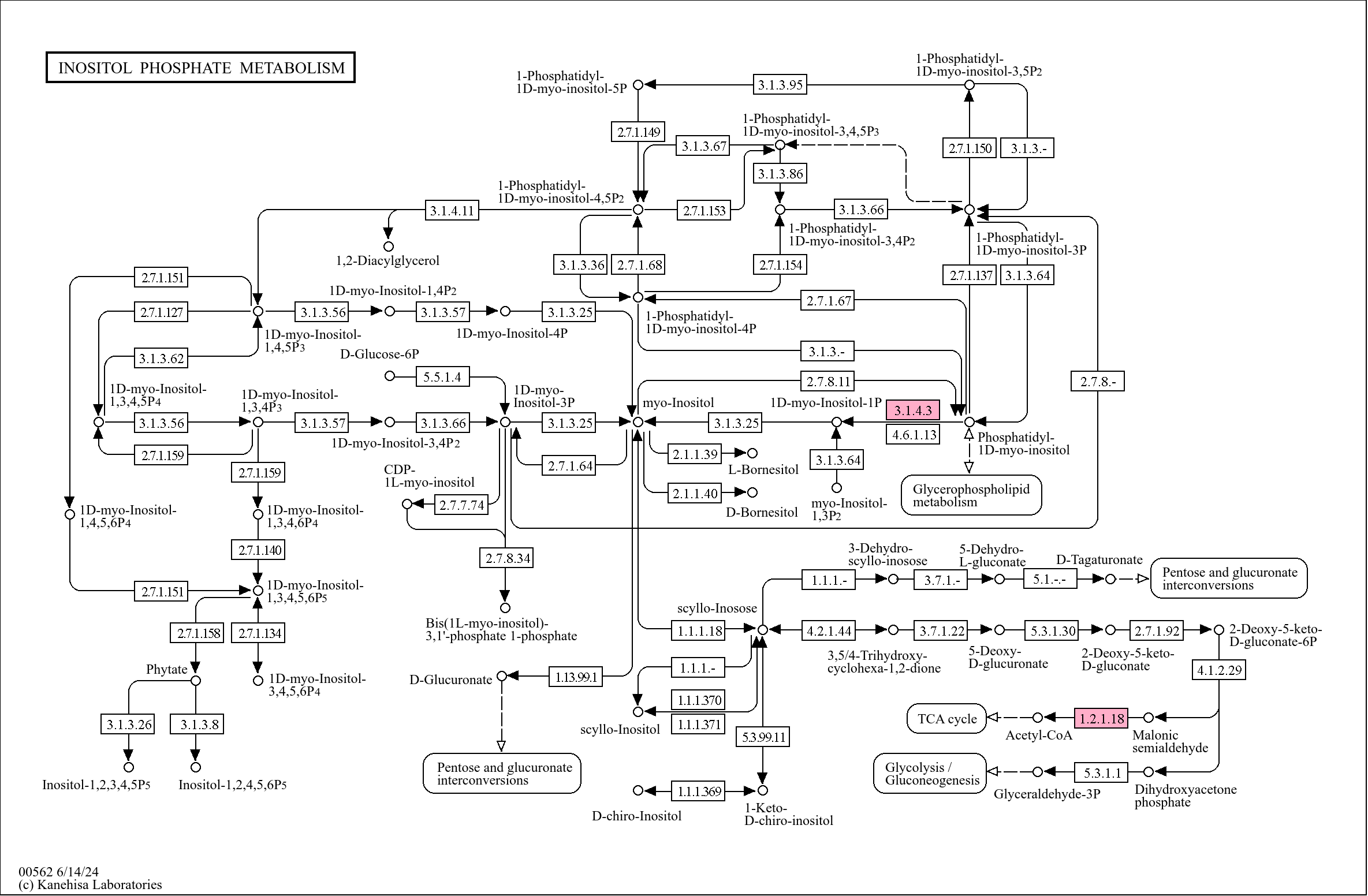
Fig. S46. Enzymatic profile of the "Inositol phosphate metabolism" KEGG pathway in the oral microbiome, exhibiting a ≥ 0.2-fold decrease in abundance in transgenic male littermates expressing amyloid precursor protein/presenilin-1 (APP/PS1) compared to age-matched wild-type controls. EC:1.2.1.18 = Malonate-semialdehyde dehydrogenase (acetylating); EC:3.1.4.3 = Phospholipase C. None of the enzymes enriched in this pathway in oral WT microbiome showed increased abundance in the gut microbiome of WT mice relative to APP/PS1 mice. Furthermore, a substantial number of these enzymes were completely undetected in the gut microbial ecosystem. Consequently, these enzymes were not considered from our most recent study examining the influence of the gut microbiome on Alzheimer's disease (AD).


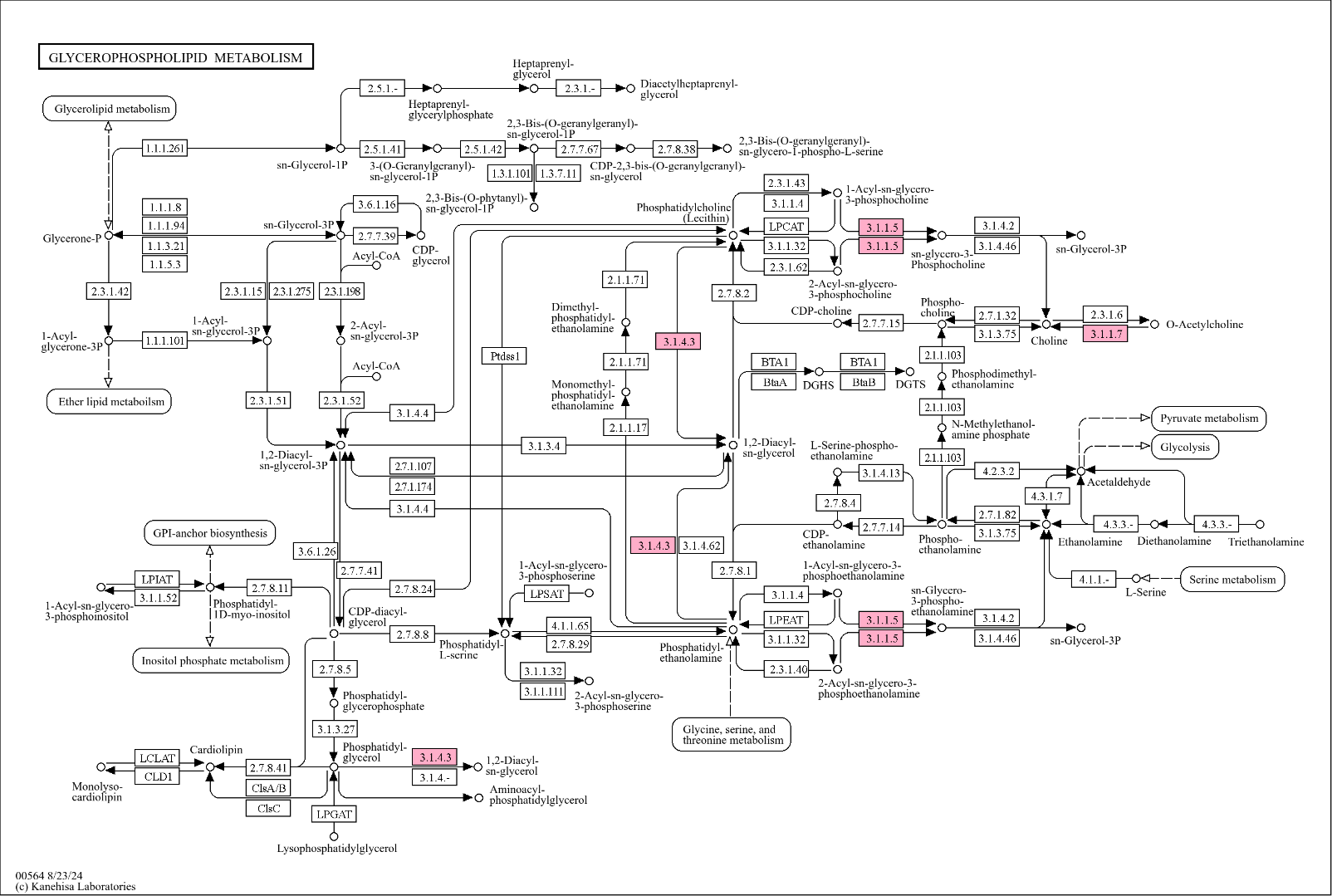
Fig. S47. Enzymatic profile of the "Glycerophospholipid metabolism" KEGG pathway in the oral microbiome, exhibiting a ≥ 0.2-fold decrease in abundance in transgenic male littermates expressing amyloid precursor protein/presenilin-1 (APP/PS1) compared to age-matched wild-type controls. EC:3.1.1.5 = Lysophospholipase; EC:3.1.4.3 = Phospholipase C; *EC:3.1.1.7 = Acetylcholinesterase. The red asterisk (*) symbol functions as a crucial visual marker, highlighting specific KEGG enzymes that have been either substantiated through scientific inquiry or are hypothesized to potentially play pivotal roles in the onset or progression of Alzheimer’s disease (AD). None of the enzymes enriched in this pathway in oral WT microbiome showed increased abundance in the gut microbiome of WT mice relative to APP/PS1 mice. Furthermore, a substantial number of these enzymes were completely undetected in the gut microbial ecosystem. Consequently, these enzymes were not considered from our most recent study examining the influence of the gut microbiome on Alzheimer's disease (AD).


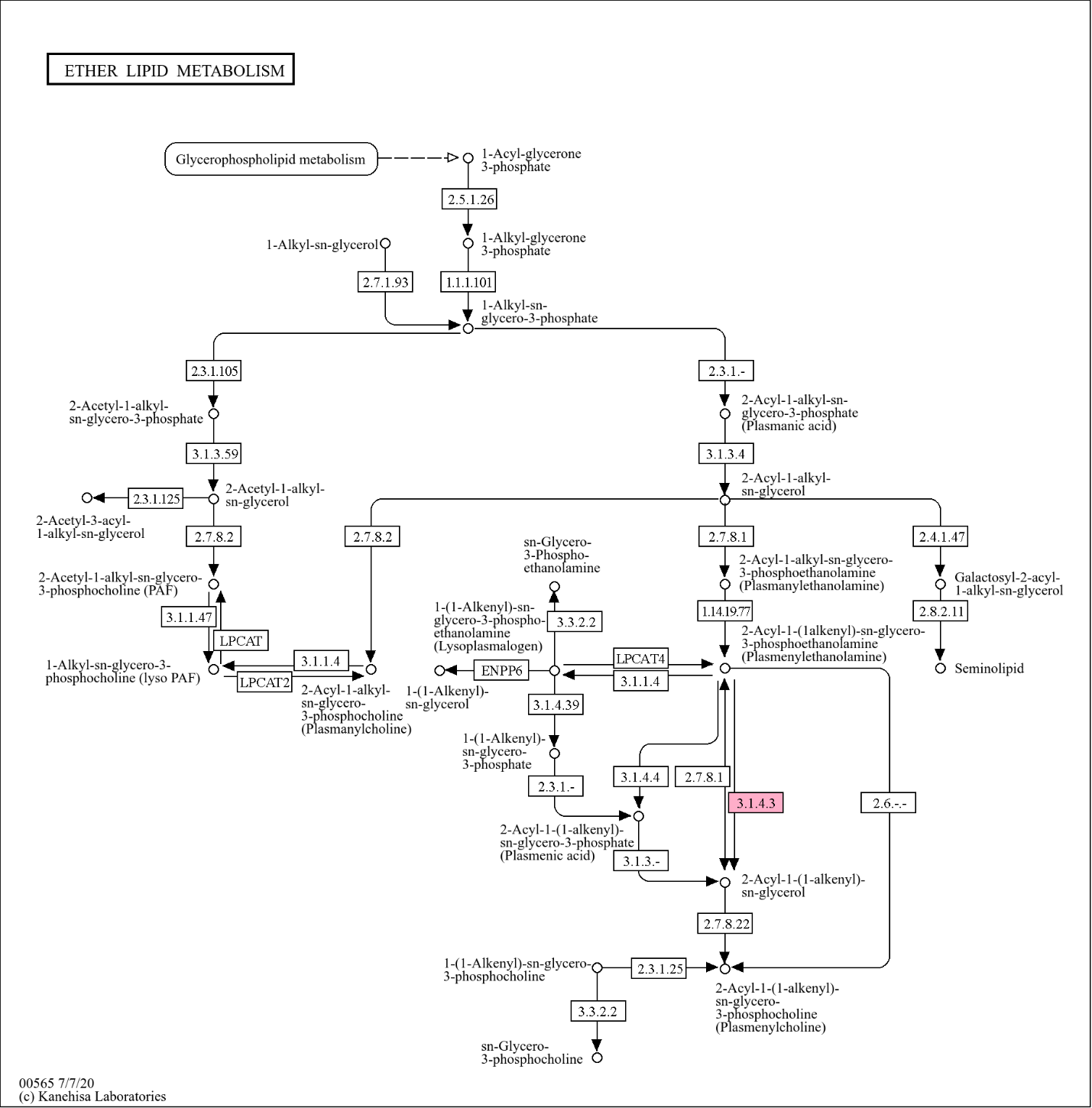
Fig. S48. Enzymatic profile of the "Ether lipid metabolism" KEGG pathway in the oral microbiome, exhibiting a ≥ 0.2-fold decrease in abundance in transgenic male littermates expressing amyloid precursor protein/presenilin-1 (APP/PS1) compared to age-matched wild-type controls. EC:3.1.4.3 = Phospholipase C. None of the enzymes enriched in this pathway in oral WT microbiome showed increased abundance in the gut microbiome of WT mice relative to APP/PS1 mice. Furthermore, a substantial number of these enzymes were completely undetected in the gut microbial ecosystem. Consequently, these enzymes were not considered from our most recent study examining the influence of the gut microbiome on Alzheimer's disease (AD).


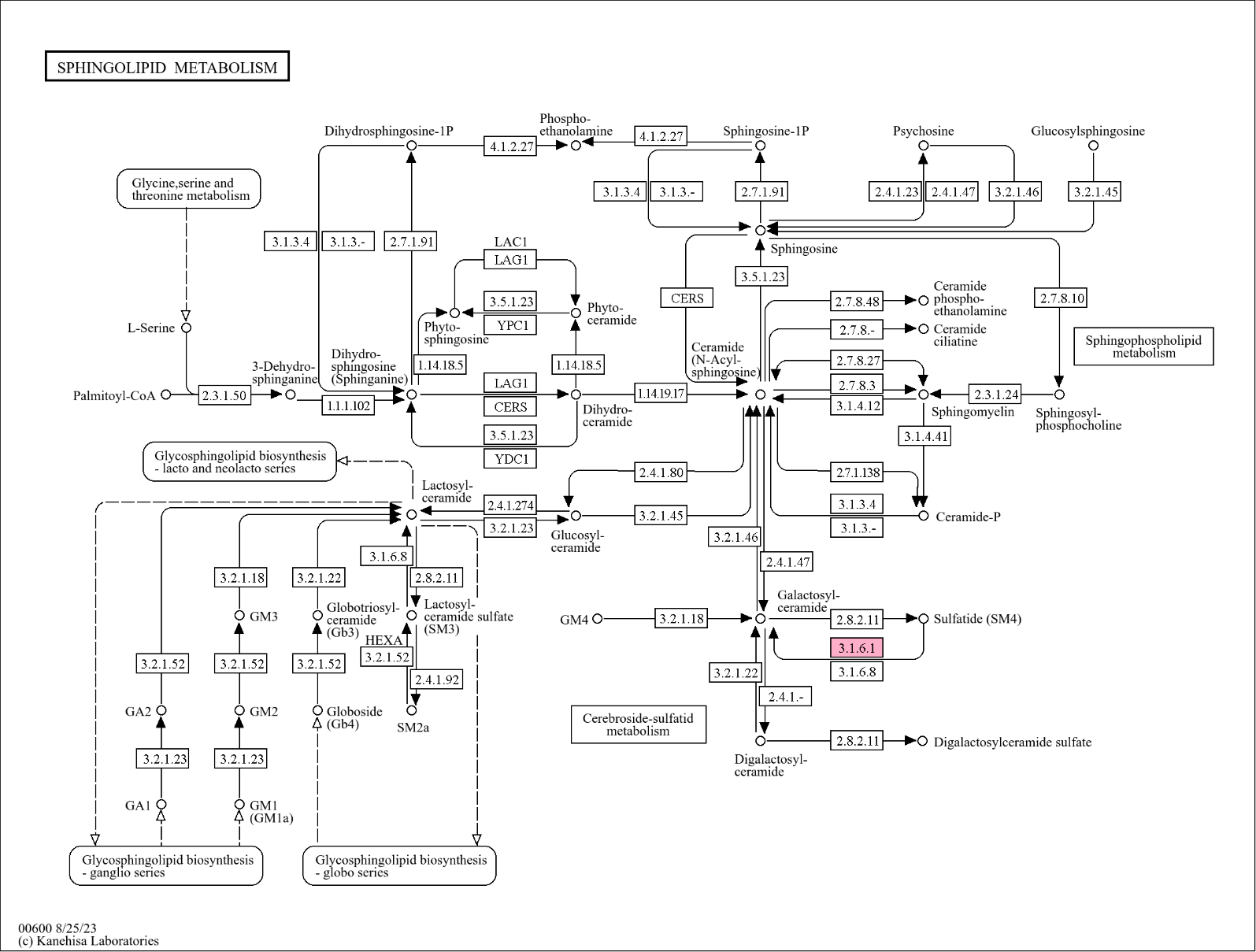
Fig. S49. Enzymatic profile of the "Sphingolipid metabolism" KEGG pathway in the oral microbiome, exhibiting a ≥ 0.2-fold decrease in abundance in transgenic male littermates expressing amyloid precursor protein/presenilin-1 (APP/PS1) compared to age-matched wild-type controls. EC:3.1.6.1 = Arylsulfatase. None of the enzymes enriched in this pathway in oral WT microbiome showed increased abundance in the gut microbiome of WT mice relative to APP/PS1 mice. Furthermore, a substantial number of these enzymes were completely undetected in the gut microbial ecosystem. Consequently, these enzymes were not considered from our most recent study examining the influence of the gut microbiome on Alzheimer's disease (AD).


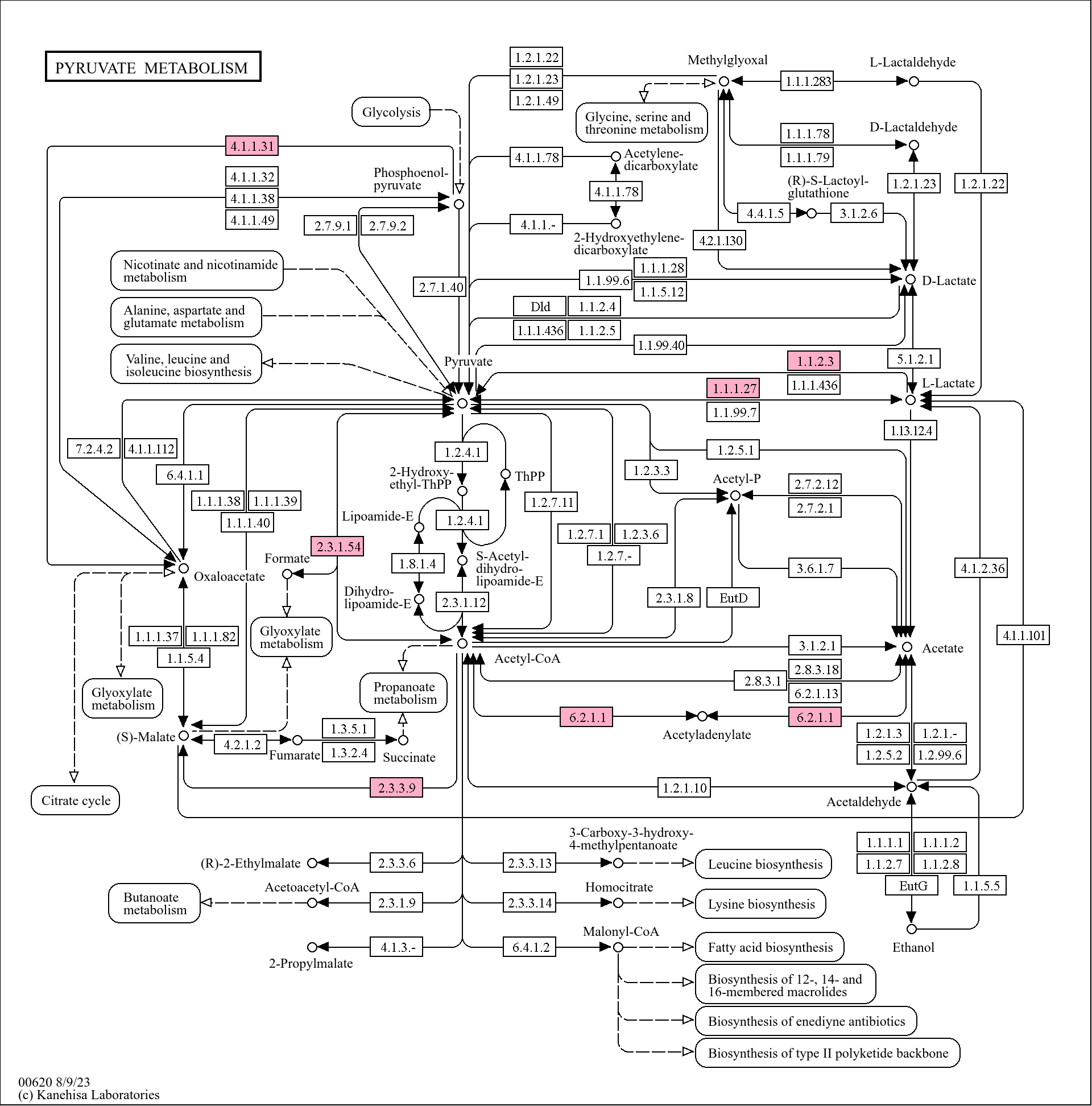
Fig. S50. Enzymatic profile of the "Pyruvate metabolism" KEGG pathway in the oral microbiome, exhibiting a ≥ 0.2-fold decrease in abundance in transgenic male littermates expressing amyloid precursor protein/presenilin-1 (APP/PS1) compared to age-matched wild-type controls. EC:1.1.2.3 = L-lactate dehydrogenase (cytochrome); EC:2.3.3.9 = Malate synthase; EC:4.1.1.31 = Phosphoenolpyruvate carboxylase; *EC:2.3.1.54 = Formate C-acetyltransferase; *EC:6.2.1.1 = Acetate--CoA ligase; *EC:1.1.1.27 = L-lactate dehydrogenase. The red asterisk (*) symbol functions as a crucial visual marker, highlighting specific KEGG enzymes that have been either substantiated through scientific inquiry or are hypothesized to potentially play pivotal roles in the onset or progression of Alzheimer’s disease (AD). None of the enzymes enriched in this pathway in oral WT microbiome showed increased abundance in the gut microbiome of WT mice relative to APP/PS1 mice. Furthermore, a substantial number of these enzymes were completely undetected in the gut microbial ecosystem. Consequently, these enzymes were not considered from our most recent study examining the influence of the gut microbiome on Alzheimer's disease (AD).


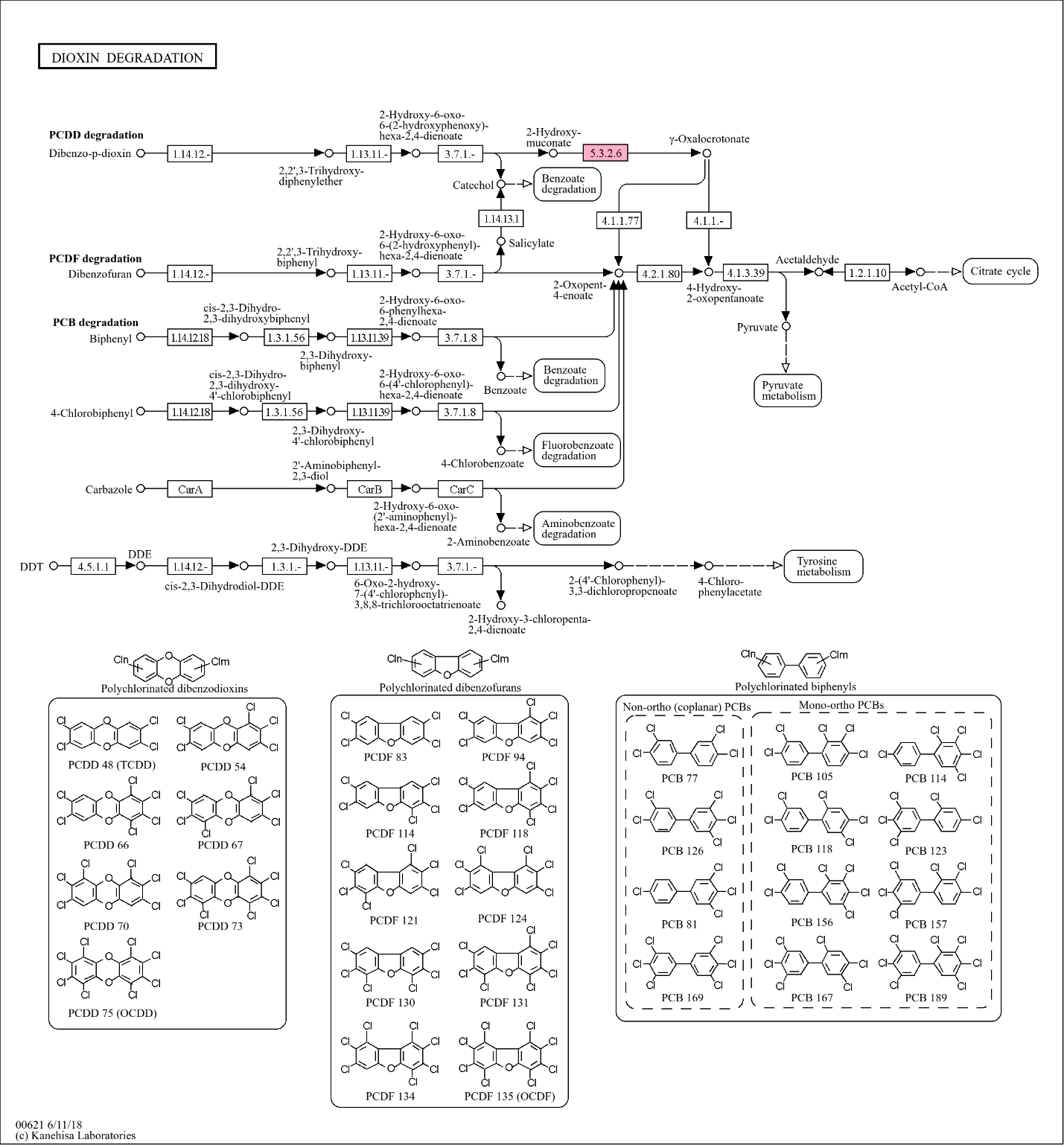
Fig. S51. Enzymatic profile of the "Dioxin degradation" KEGG pathway in the oral microbiome, exhibiting a ≥ 0.2-fold decrease in abundance in transgenic male littermates expressing amyloid precursor protein/presenilin-1 (APP/PS1) compared to age-matched wild-type controls. EC:5.3.2.6 = 2-hydroxymuconate tautomerase. None of the enzymes enriched in this pathway in oral WT microbiome showed increased abundance in the gut microbiome of WT mice relative to APP/PS1 mice. Furthermore, a substantial number of these enzymes were completely undetected in the gut microbial ecosystem. Consequently, these enzymes were not considered from our most recent study examining the influence of the gut microbiome on Alzheimer's disease (AD).


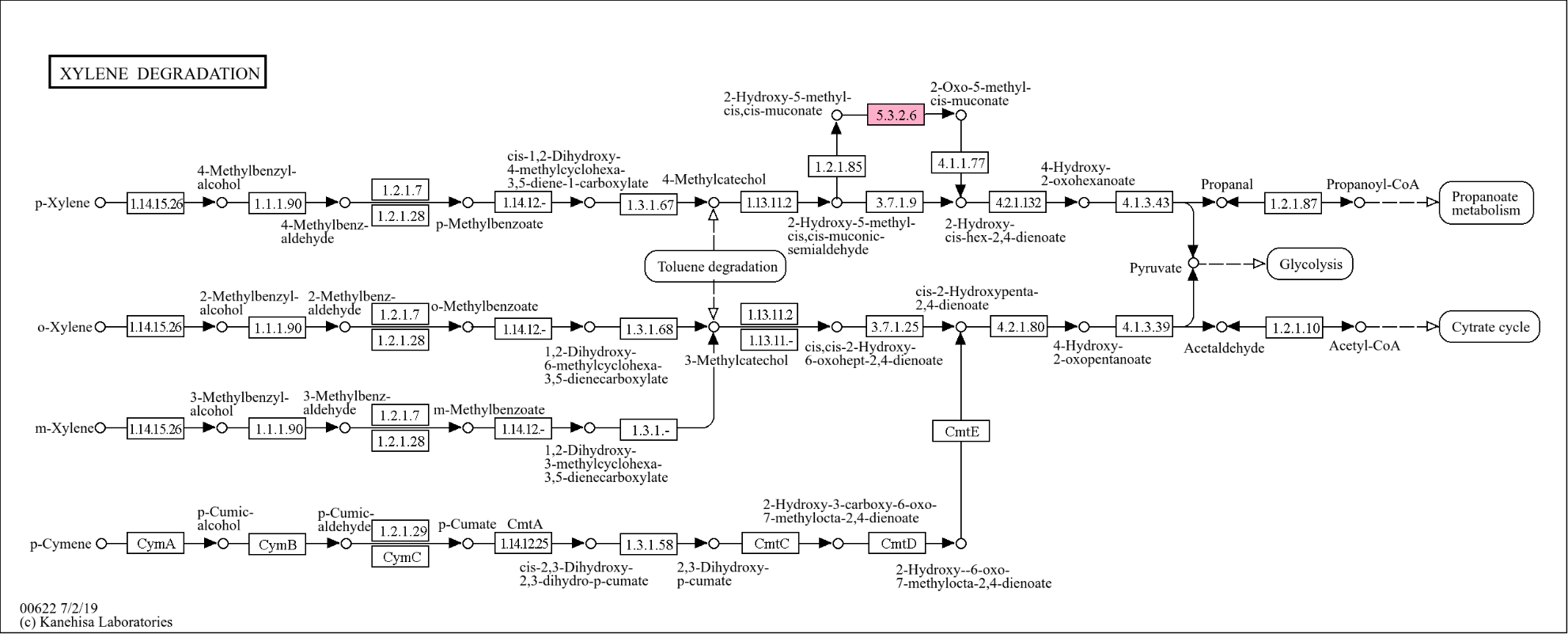
Fig. S52. Enzymatic profile of the "Xylene degradation" KEGG pathway in the oral microbiome, exhibiting a ≥ 0.2-fold decrease in abundance in transgenic male littermates expressing amyloid precursor protein/presenilin-1 (APP/PS1) compared to age-matched wild-type controls. EC:5.3.2.6 = 2-hydroxymuconate tautomerase. None of the enzymes enriched in this pathway in oral WT microbiome showed increased abundance in the gut microbiome of WT mice relative to APP/PS1 mice. Furthermore, a substantial number of these enzymes were completely undetected in the gut microbial ecosystem. Consequently, these enzymes were not considered from our most recent study examining the influence of the gut microbiome on Alzheimer's disease (AD).


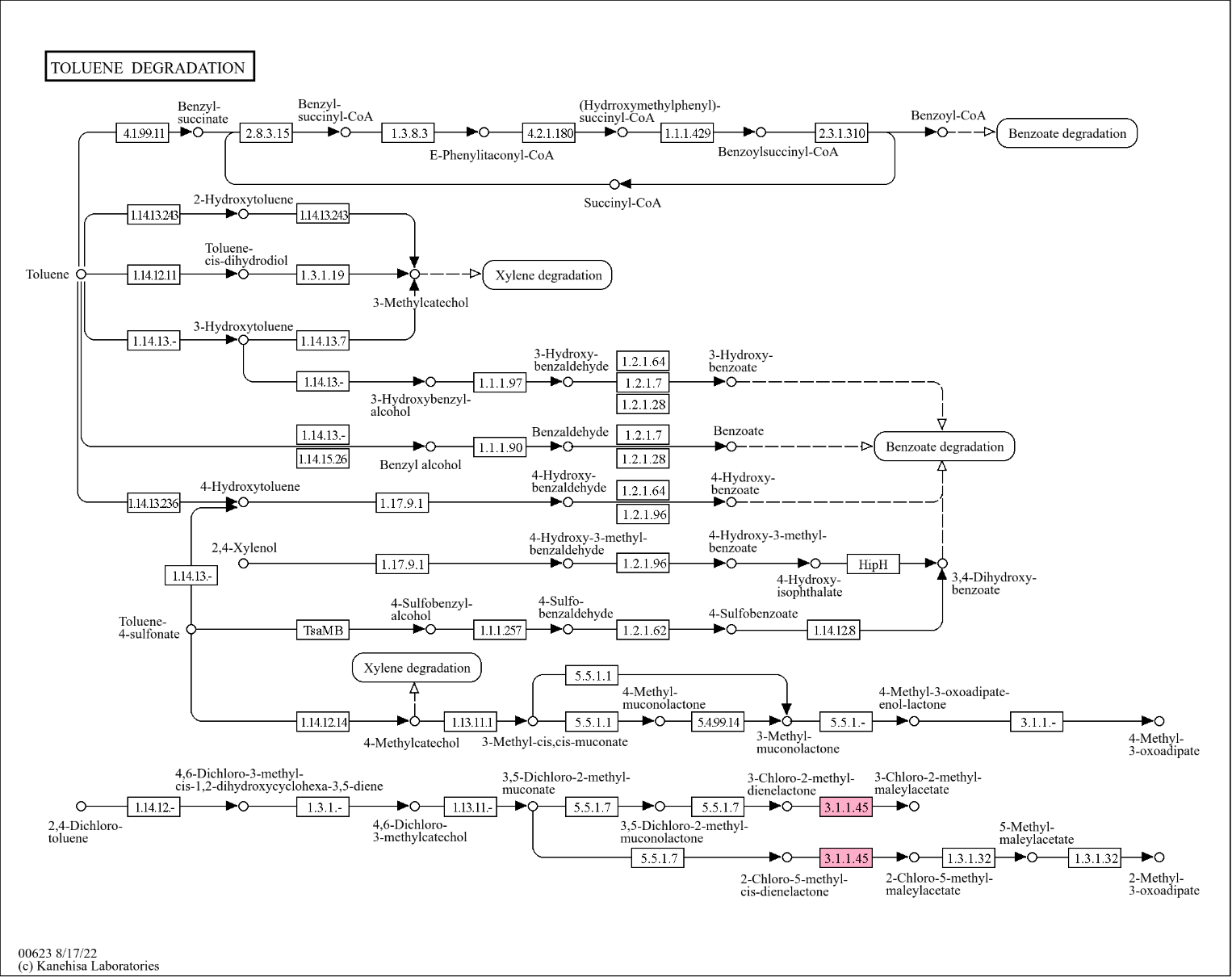
Fig. S53. Enzymatic profile of the "Toluene degradation" KEGG pathway in the oral microbiome, exhibiting a ≥ 0.2-fold decrease in abundance in transgenic male littermates expressing amyloid precursor protein/presenilin-1 (APP/PS1) compared to age-matched wild-type controls. EC:3.1.1.45 = Carboxymethylene butenolidase. None of the enzymes enriched in this pathway in oral WT microbiome showed increased abundance in the gut microbiome of WT mice relative to APP/PS1 mice. Furthermore, a substantial number of these enzymes were completely undetected in the gut microbial ecosystem. Consequently, these enzymes were not considered from our most recent study examining the influence of the gut microbiome on Alzheimer's disease (AD).


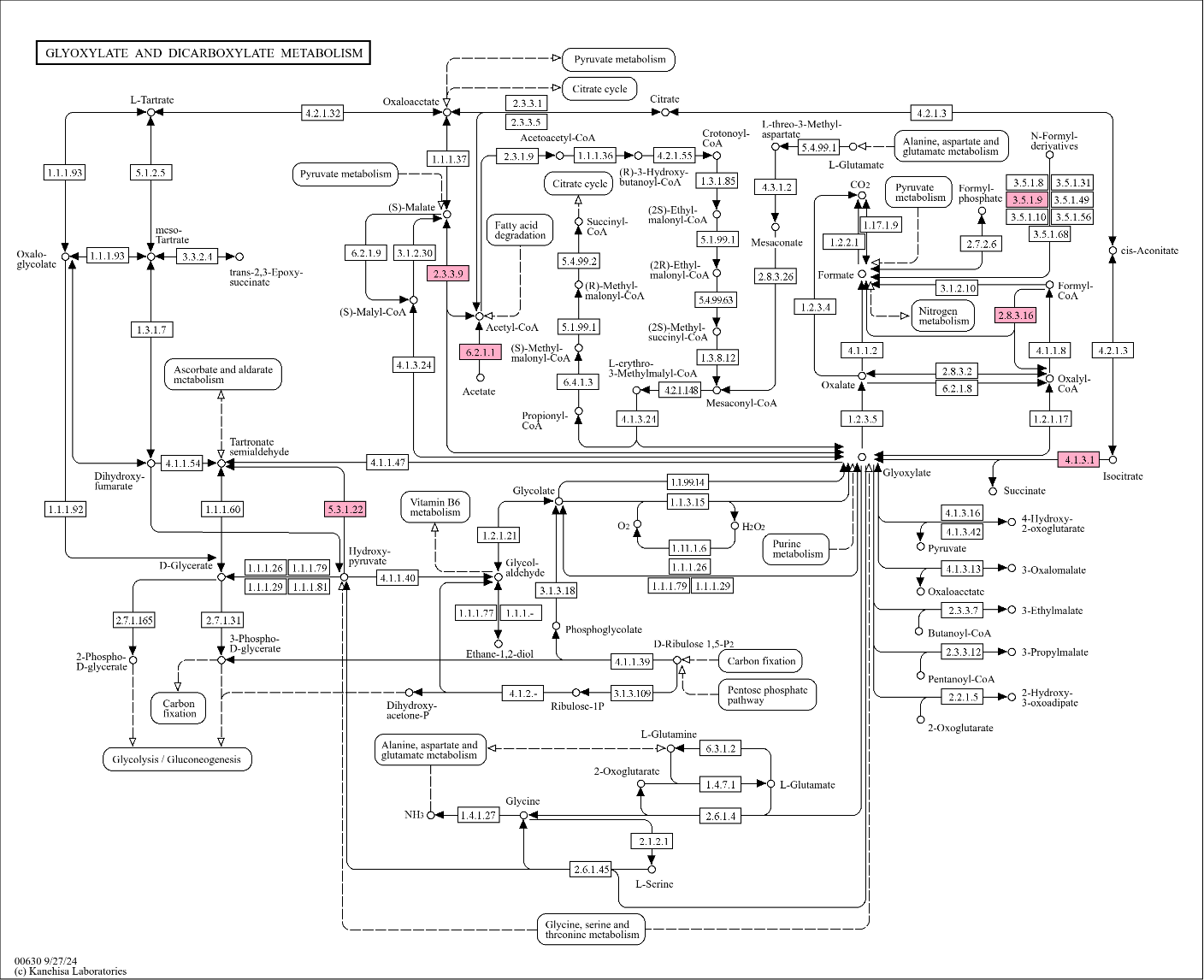
Fig. S54. Enzymatic profile of the "Glyoxylate and dicarboxylate metabolism" KEGG pathway in the oral microbiome, exhibiting a ≥ 0.2-fold decrease in abundance in transgenic male littermates expressing amyloid precursor protein/presenilin-1 (APP/PS1) compared to age-matched wild-type controls. EC:2.3.3.9 = Malate synthase; EC:3.5.1.9 = Arylformamidase; EC:4.1.3.1 = Isocitrate lyase; EC:5.3.1.22 = Hydroxypyruvate isomerase; *EC:6.2.1.1 = Acetate--CoA ligase; EC:2.8.3.16 = Formyl-CoA transferase. The red asterisk (*) symbol functions as a crucial visual marker, highlighting specific KEGG enzymes that have been either substantiated through scientific inquiry or are hypothesized to potentially play pivotal roles in the onset or progression of Alzheimer’s disease (AD). None of the enzymes enriched in this pathway in oral WT microbiome showed increased abundance in the gut microbiome of WT mice relative to APP/PS1 mice. Furthermore, a substantial number of these enzymes were completely undetected in the gut microbial ecosystem. Consequently, these enzymes were not considered from our most recent study examining the influence of the gut microbiome on Alzheimer's disease (AD).


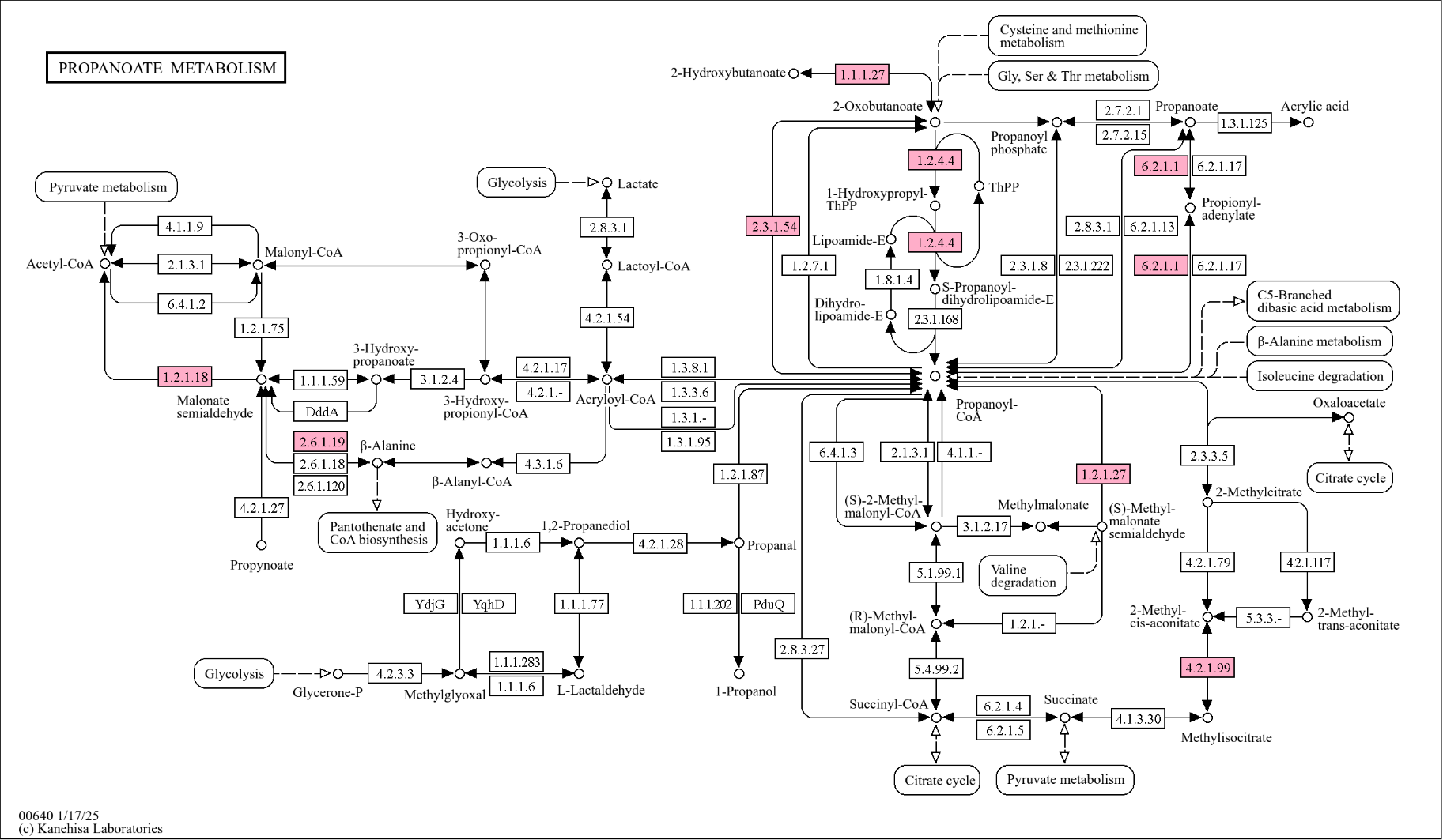
Fig. S55. Enzymatic profile of the "Propanoate metabolism" KEGG pathway in the oral microbiome, exhibiting a ≥ 0.2-fold decrease in abundance in transgenic male littermates expressing amyloid precursor protein/presenilin-1 (APP/PS1) compared to age-matched wild-type controls. EC:1.2.1.18 = Malonate-semialdehyde dehydrogenase (acetylating); EC:1.2.1.27 = Methylmalonate-semialdehyde dehydrogenase (CoA acylating); EC:1.2.4.4 = 3-Methyl-2-oxobutanoate dehydrogenase (2-methylpropanoyl-transferring); EC:2.6.1.19 = 4-aminobutyrate--2-oxoglutarate transaminase; EC:4.2.1.99 = 2-methylisocitrate dehydratase; *EC:2.3.1.54 = Formate C-acetyltransferase; EC:6.2.1.1 = Acetate--CoA ligase; EC:1.1.1.27 = L-lactate dehydrogenase. The red asterisk (*) symbol functions as a crucial visual marker, highlighting specific KEGG enzymes that have been either substantiated through scientific inquiry or are hypothesized to potentially play pivotal roles in the onset or progression of Alzheimer’s disease (AD). None of the enzymes enriched in this pathway in oral WT microbiome showed increased abundance in the gut microbiome of WT mice relative to APP/PS1 mice. Furthermore, a substantial number of these enzymes were completely undetected in the gut microbial ecosystem. Consequently, these enzymes were not considered from our most recent study examining the influence of the gut microbiome on Alzheimer's disease (AD).


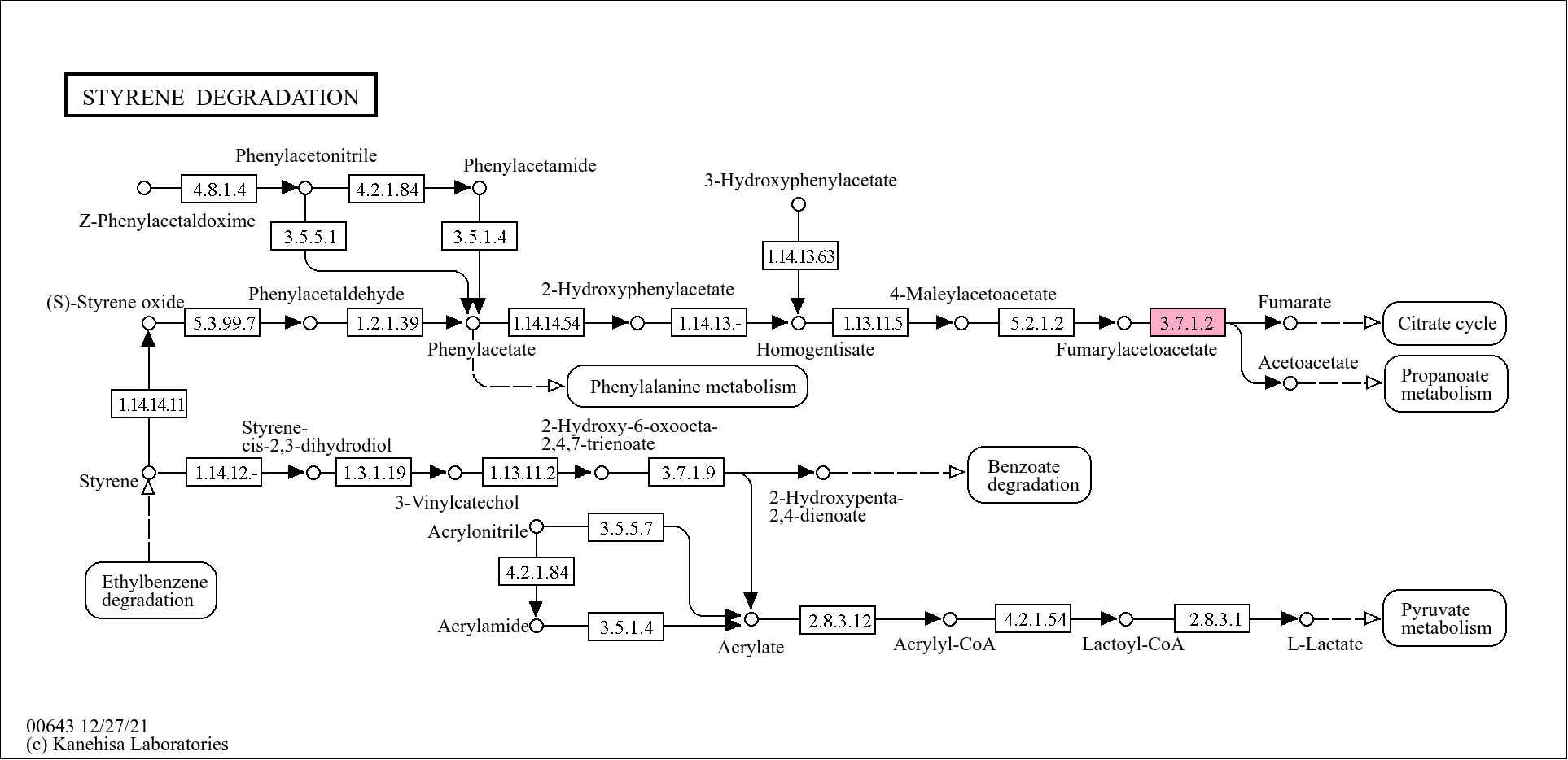
Fig. S56. Enzymatic profile of the "Styrene degradation" KEGG pathway in the oral microbiome, exhibiting a ≥ 0.2-fold decrease in abundance in transgenic male littermates expressing amyloid precursor protein/presenilin-1 (APP/PS1) compared to age-matched wild-type controls. EC:3.7.1.2 = Fumaryl acetoacetase. None of the enzymes enriched in this pathway in oral WT microbiome showed increased abundance in the gut microbiome of WT mice relative to APP/PS1 mice. Furthermore, a substantial number of these enzymes were completely undetected in the gut microbial ecosystem. Consequently, these enzymes were not considered from our most recent study examining the influence of the gut microbiome on Alzheimer's disease (AD).


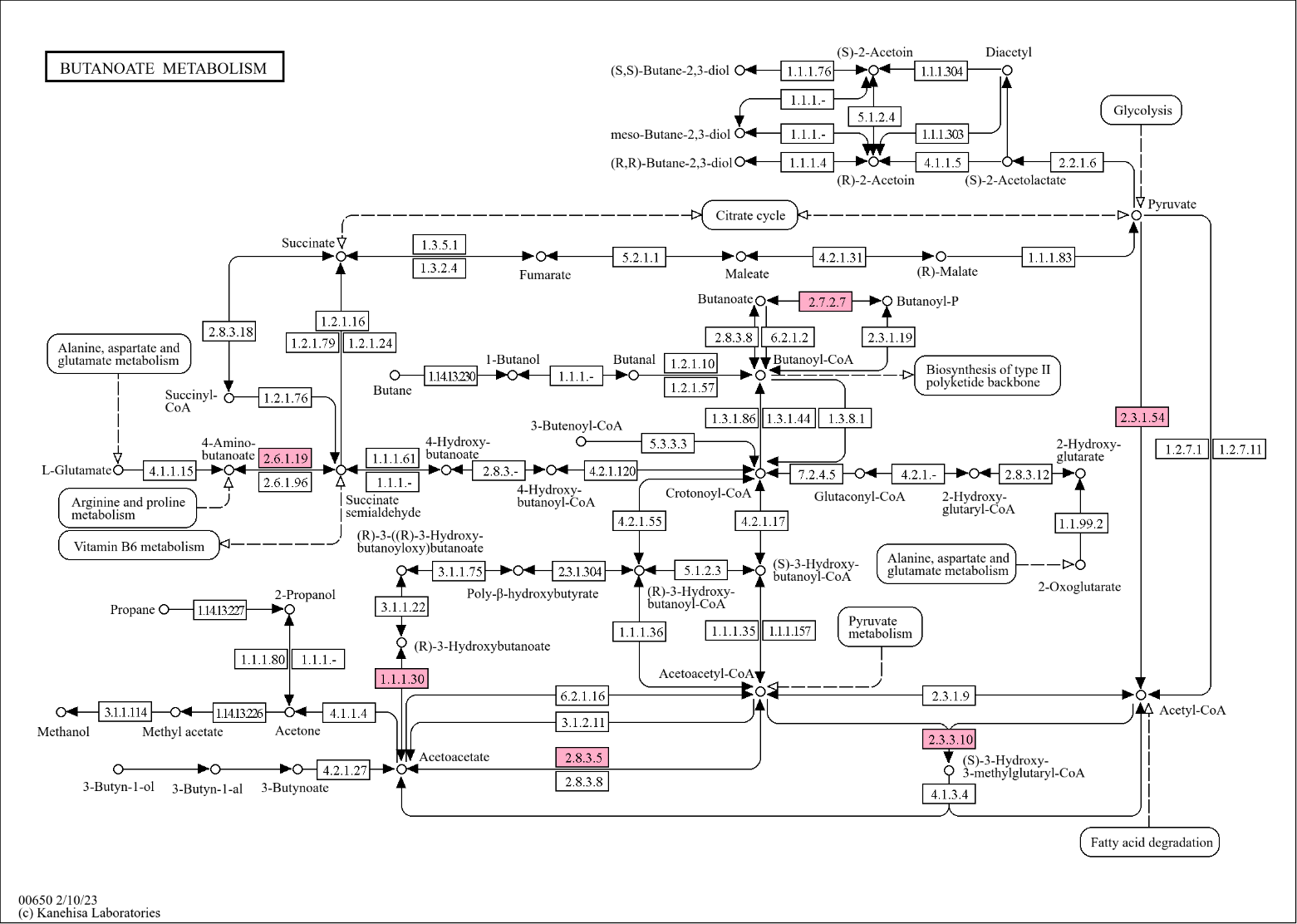
Fig. S57. Enzymatic profile of the "Butanoate metabolism" KEGG pathway in the oral microbiome, exhibiting a ≥ 0.2-fold decrease in abundance in transgenic male littermates expressing amyloid precursor protein/presenilin-1 (APP/PS1) compared to age-matched wild-type controls. EC:1.1.1.30 = 3-hydroxybutyrate dehydrogenase; EC:2.3.3.10 = Hydroxymethylglutaryl-CoA synthase; EC:2.6.1.19 = 4-aminobutyrate--2-oxoglutarate transaminase; EC:2.8.3.5 = 3-Oxoacid CoA-transferase; *EC:2.3.1.54 = Formate C-acetyltransferase; *EC:2.7.2.7 = Butyrate kinase. The red asterisk (*) symbol functions as a crucial visual marker, highlighting specific KEGG enzymes that have been either substantiated through scientific inquiry or are hypothesized to potentially play pivotal roles in the onset or progression of Alzheimer’s disease (AD). None of the enzymes enriched in this pathway in oral WT microbiome showed increased abundance in the gut microbiome of WT mice relative to APP/PS1 mice. Furthermore, a substantial number of these enzymes were completely undetected in the gut microbial ecosystem. Consequently, these enzymes were not considered from our most recent study examining the influence of the gut microbiome on Alzheimer's disease (AD).


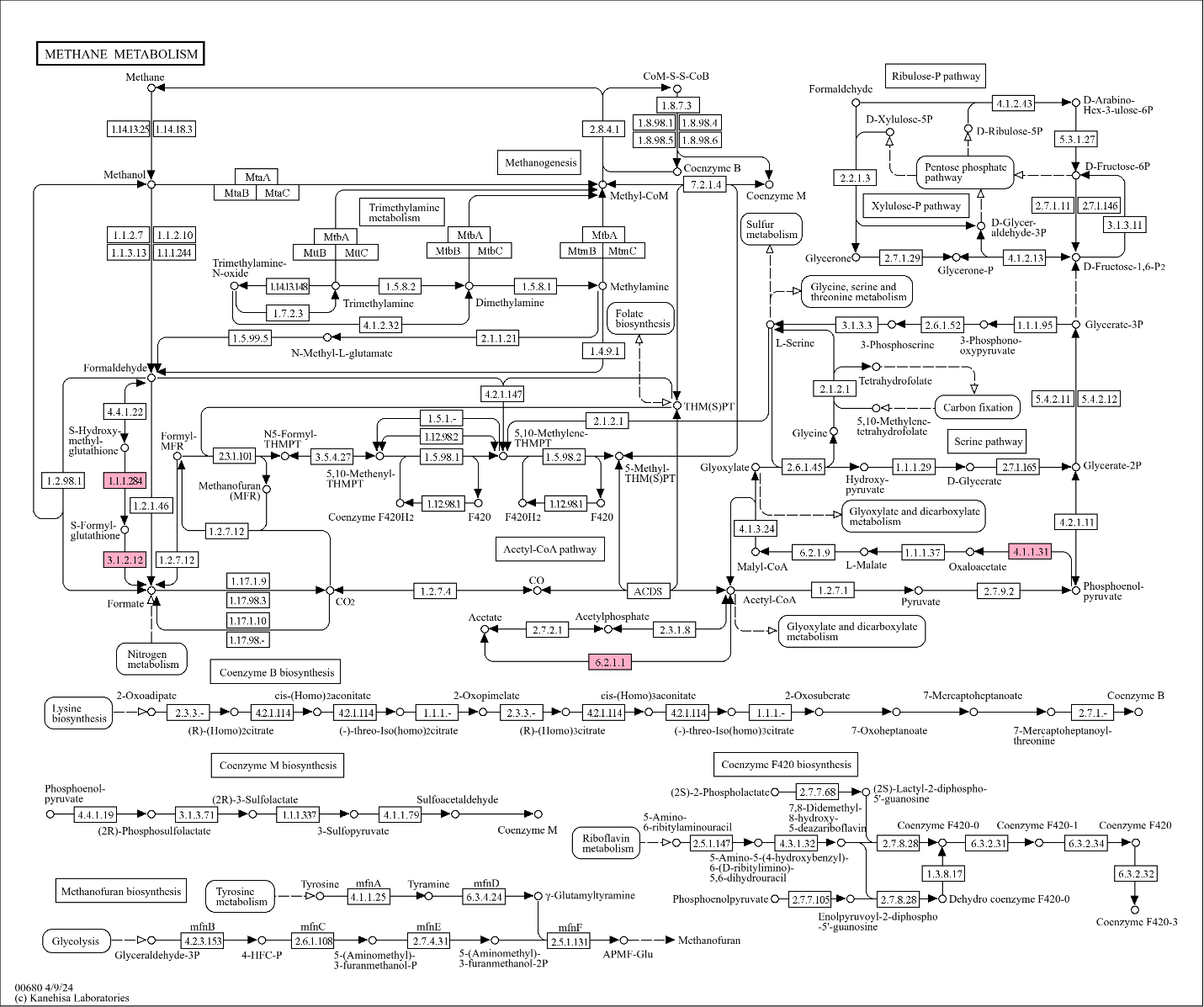
Fig. S58. Enzymatic profile of the "Methane metabolism" KEGG pathway in the oral microbiome, exhibiting a ≥ 0.2-fold decrease in abundance in transgenic male littermates expressing amyloid precursor protein/presenilin-1 (APP/PS1) compared to age-matched wild-type controls. EC:1.1.1.284 = S-(hydroxymethyl)glutathione dehydrogenase; EC:3.1.2.12 = S-formylglutathione hydrolase; EC:4.1.1.31 = Phosphoenolpyruvate carboxylase; *EC:6.2.1.1 = Acetate--CoA ligase. The red asterisk (*) symbol functions as a crucial visual marker, highlighting specific KEGG enzymes that have been either substantiated through scientific inquiry or are hypothesized to potentially play pivotal roles in the onset or progression of Alzheimer’s disease (AD). None of the enzymes enriched in this pathway in oral WT microbiome showed increased abundance in the gut microbiome of WT mice relative to APP/PS1 mice. Furthermore, a substantial number of these enzymes were completely undetected in the gut microbial ecosystem. Consequently, these enzymes were not considered from our most recent study examining the influence of the gut microbiome on Alzheimer's disease (AD).


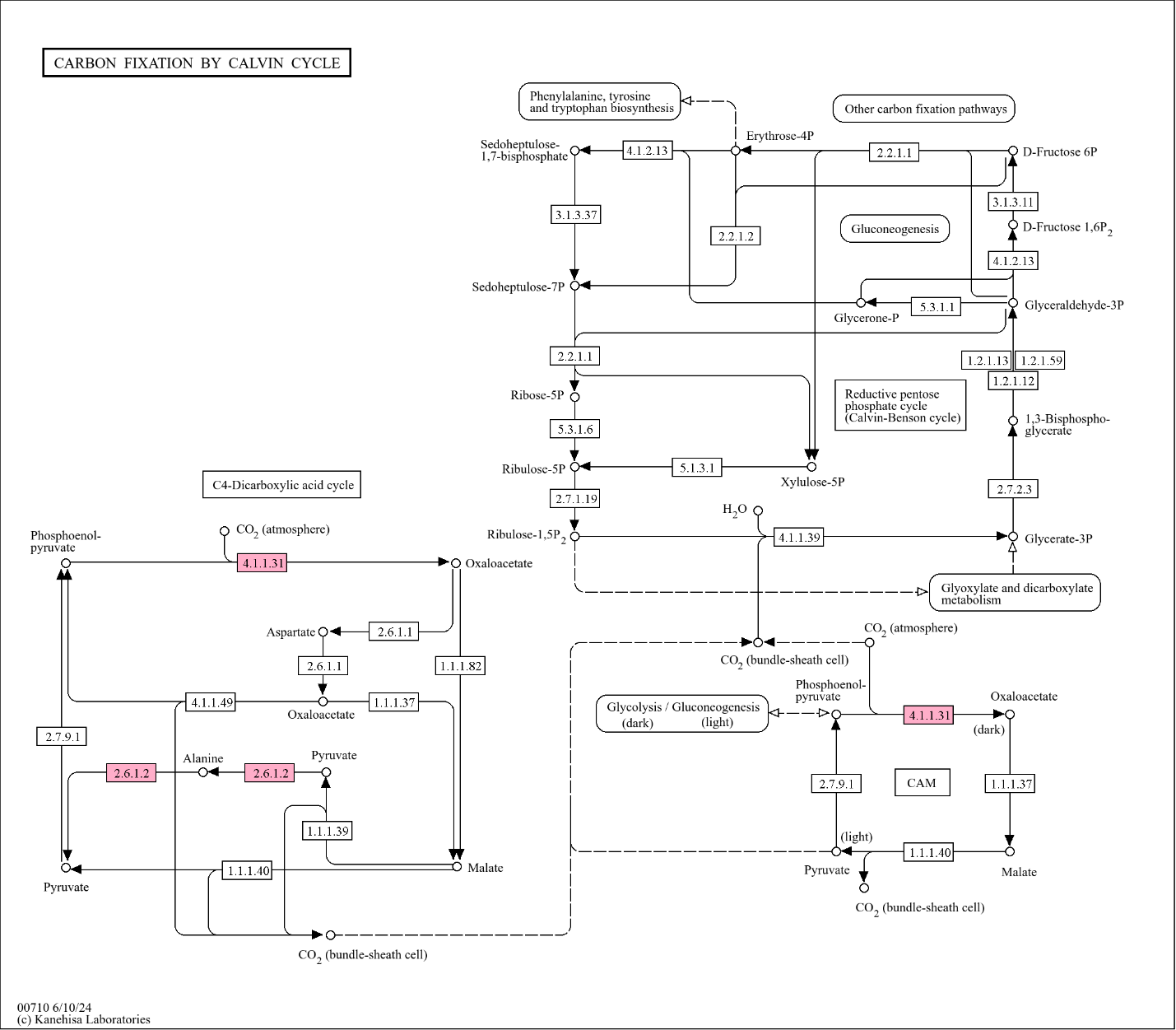
Fig. S59. Enzymatic profile of the "Carbon fixation by Calvin cycle" KEGG pathway in the oral microbiome, exhibiting a ≥ 0.2-fold decrease in abundance in transgenic male littermates expressing amyloid precursor protein/presenilin-1 (APP/PS1) compared to age-matched wild-type controls. EC:2.6.1.2 = Alanine transaminase; EC:4.1.1.31 = Phosphoenolpyruvate carboxylase. None of the enzymes enriched in this pathway in oral WT microbiome showed increased abundance in the gut microbiome of WT mice relative to APP/PS1 mice. Furthermore, a substantial number of these enzymes were completely undetected in the gut microbial ecosystem. Consequently, these enzymes were not considered from our most recent study examining the influence of the gut microbiome on Alzheimer's disease (AD).


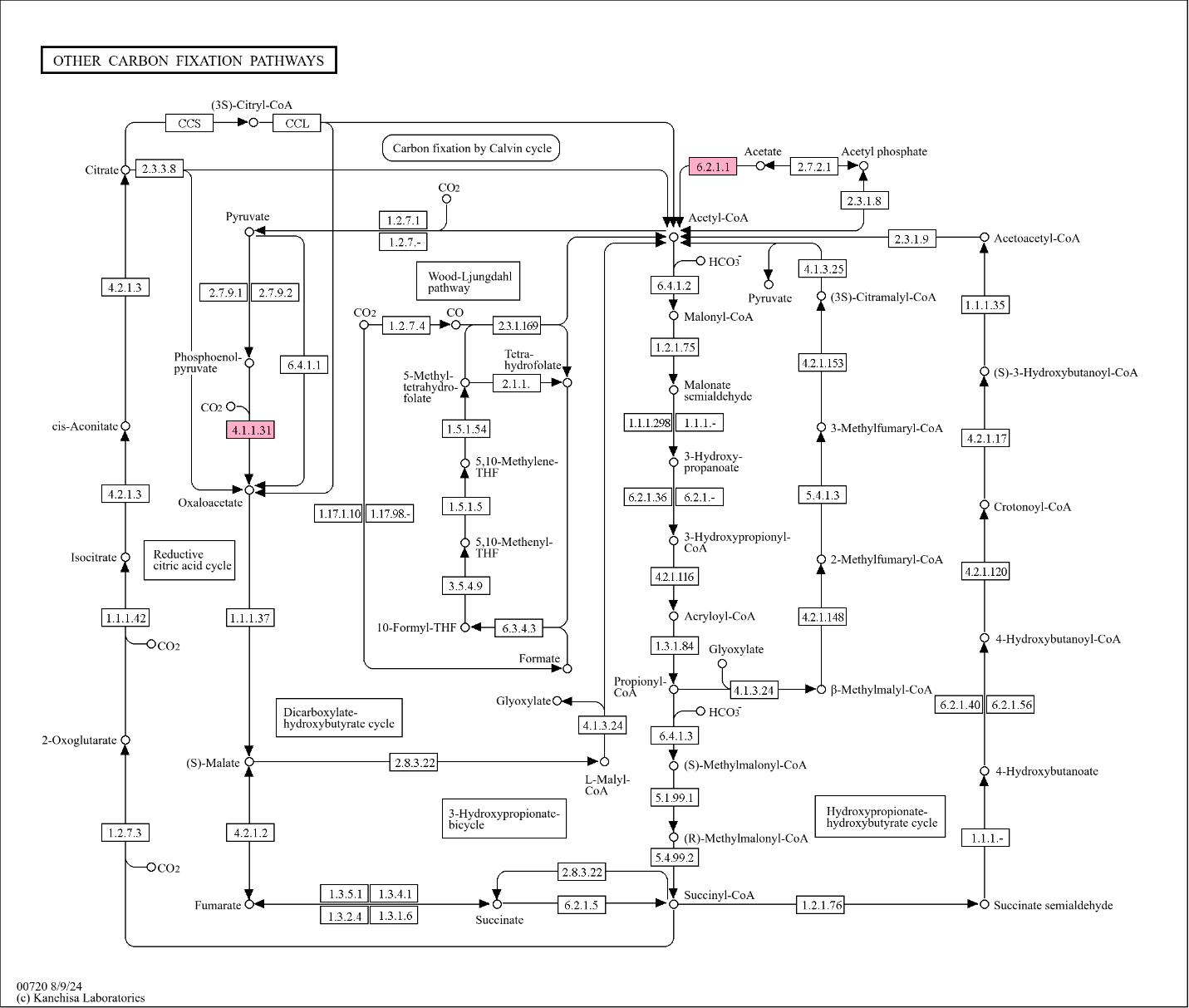
Fig. S60. Enzymatic profile of the "Other carbon fixation pathways" KEGG pathway in the oral microbiome, exhibiting a ≥ 0.2-fold decrease in abundance in transgenic male littermates expressing amyloid precursor protein/presenilin-1 (APP/PS1) compared to age-matched wild-type controls. EC:4.1.1.31 = Phosphoenolpyruvate carboxylase; *EC:6.2.1.1 = Acetate--CoA ligase. The red asterisk (*) symbol functions as a crucial visual marker, highlighting specific KEGG enzymes that have been either substantiated through scientific inquiry or are hypothesized to potentially play pivotal roles in the onset or progression of Alzheimer’s disease (AD). None of the enzymes enriched in this pathway in oral WT microbiome showed increased abundance in the gut microbiome of WT mice relative to APP/PS1 mice. Furthermore, a substantial number of these enzymes were completely undetected in the gut microbial ecosystem. Consequently, these enzymes were not considered from our most recent study examining the influence of the gut microbiome on Alzheimer's disease (AD).


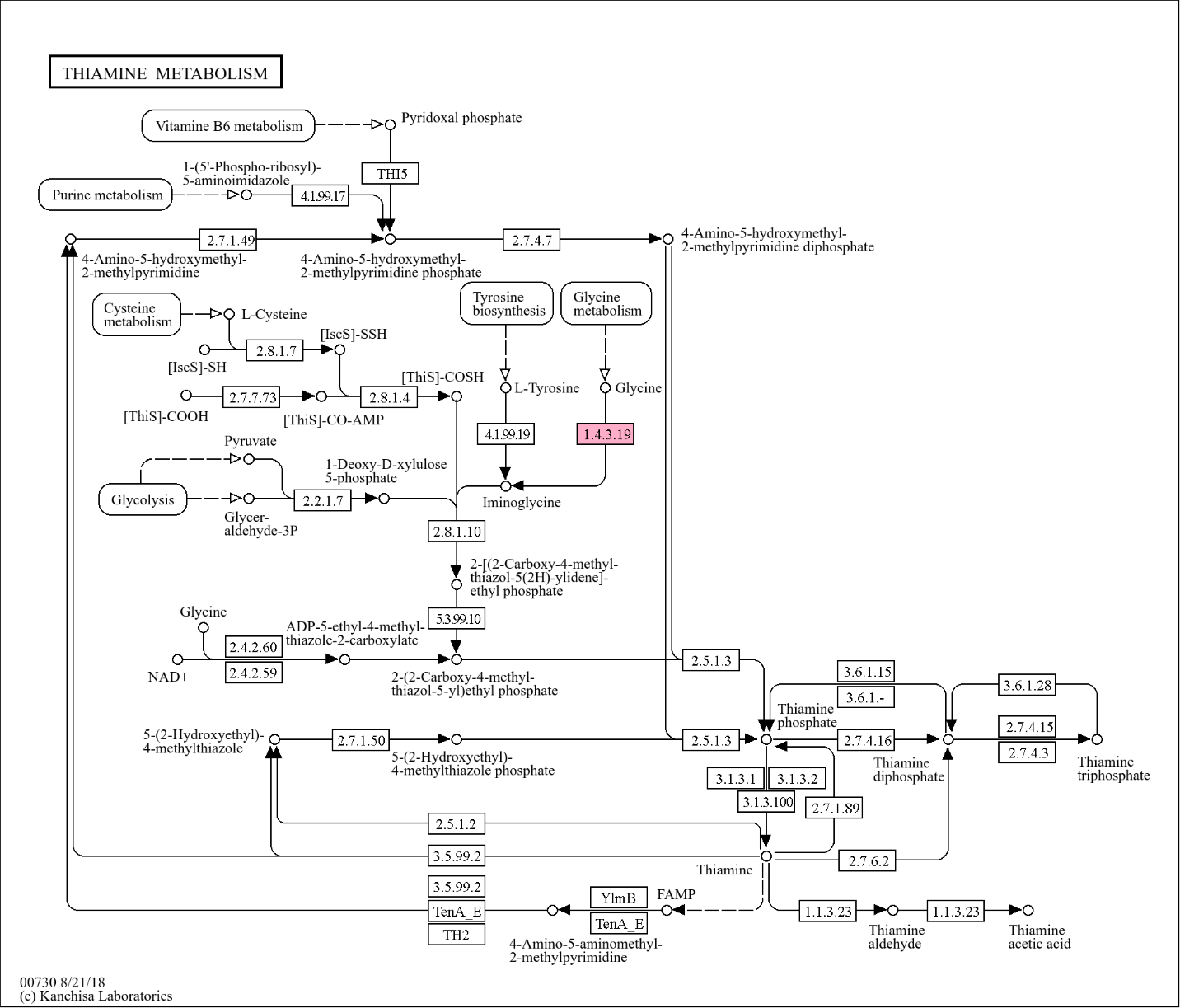
Fig. S61. Enzymatic profile of the "Thiamine metabolism" KEGG pathway in the oral microbiome, exhibiting a ≥ 0.2-fold decrease in abundance in transgenic male littermates expressing amyloid precursor protein/presenilin-1 (APP/PS1) compared to age-matched wild-type controls. EC:1.4.3.19 = Glycine oxidase. None of the enzymes enriched in this pathway in oral WT microbiome showed increased abundance in the gut microbiome of WT mice relative to APP/PS1 mice. Furthermore, a substantial number of these enzymes were completely undetected in the gut microbial ecosystem. Consequently, these enzymes were not considered from our most recent study examining the influence of the gut microbiome on Alzheimer's disease (AD).


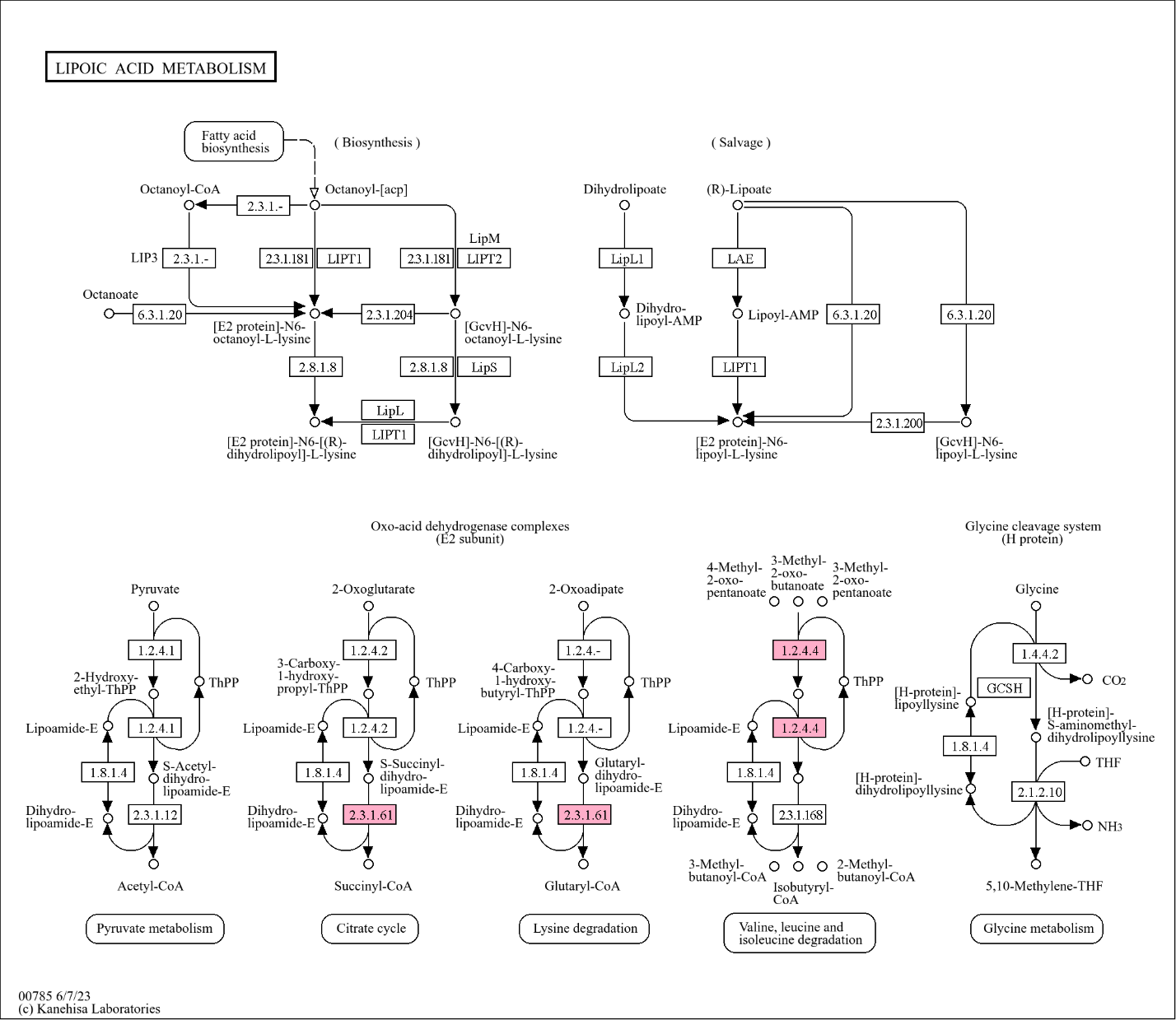
Fig. S62. Enzymatic profile of the "Lipoic acid metabolism" KEGG pathway in the oral microbiome, exhibiting a ≥ 0.2-fold decrease in abundance in transgenic male littermates expressing amyloid precursor protein/presenilin-1 (APP/PS1) compared to age-matched wild-type controls. EC:1.2.4.4 = 3-Methyl-2-oxobutanoate dehydrogenase (2-methylpropanoyl-transferring); EC:2.3.1.61 = Dihydrolipoyllysine-residue succinyltransferase. None of the enzymes enriched in this pathway in oral WT microbiome showed increased abundance in the gut microbiome of WT mice relative to APP/PS1 mice. Furthermore, a substantial number of these enzymes were completely undetected in the gut microbial ecosystem. Consequently, these enzymes were not considered from our most recent study examining the influence of the gut microbiome on Alzheimer's disease (AD).


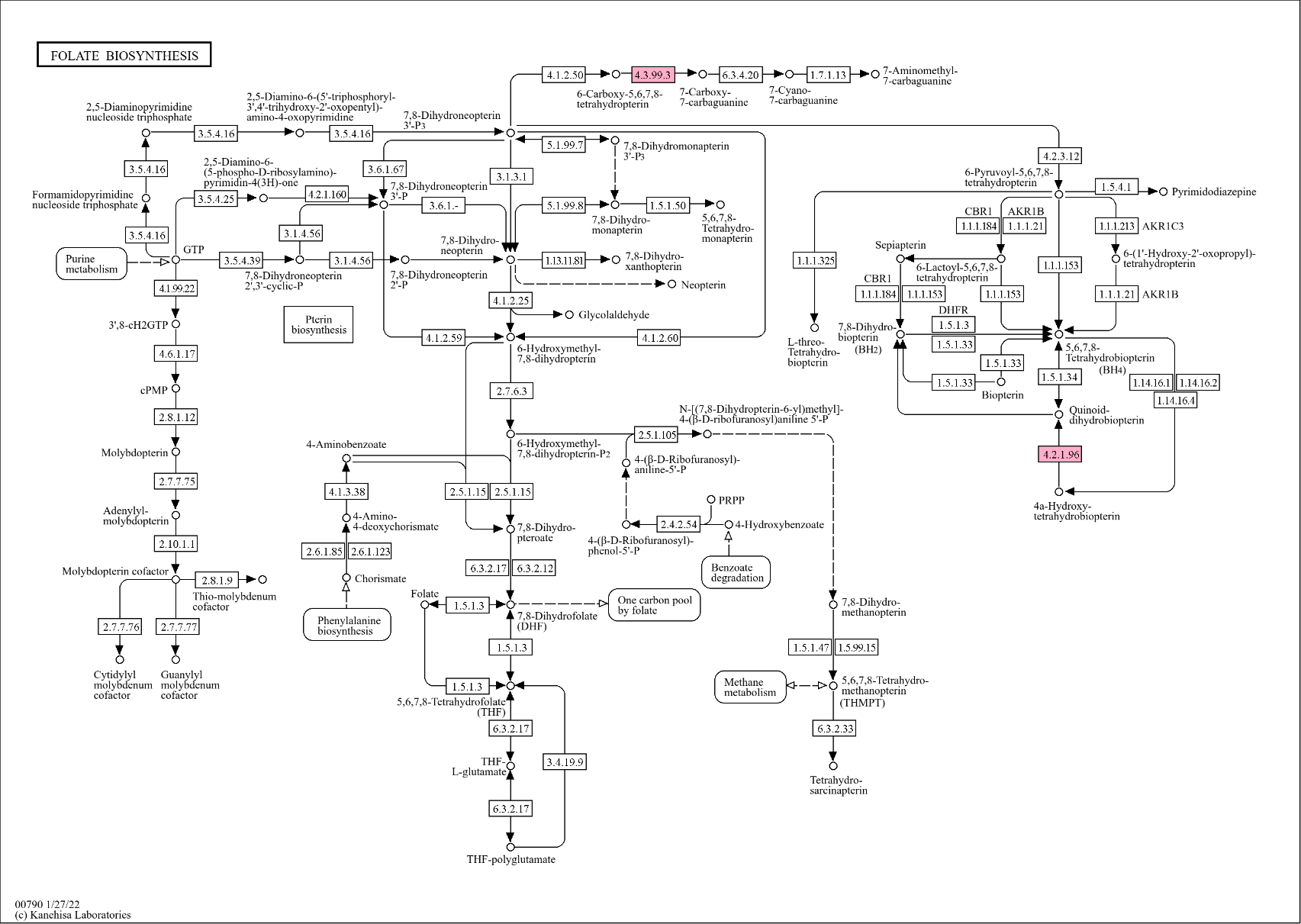
Fig. S63. Enzymatic profile of the "Folate biosynthesis" KEGG pathway in the oral microbiome, exhibiting a ≥ 0.2-fold decrease in abundance in transgenic male littermates expressing amyloid precursor protein/presenilin-1 (APP/PS1) compared to age-matched wild-type controls. EC:4.2.1.96 = 4a-hydroxytetrahydrobiopterin dehydratase; EC:4.3.99.3 = 7-carboxy-7-deazaguanine synthase. None of the enzymes enriched in this pathway in oral WT microbiome showed increased abundance in the gut microbiome of WT mice relative to APP/PS1 mice. Furthermore, a substantial number of these enzymes were completely undetected in the gut microbial ecosystem. Consequently, these enzymes were not considered from our most recent study examining the influence of the gut microbiome on Alzheimer's disease (AD).


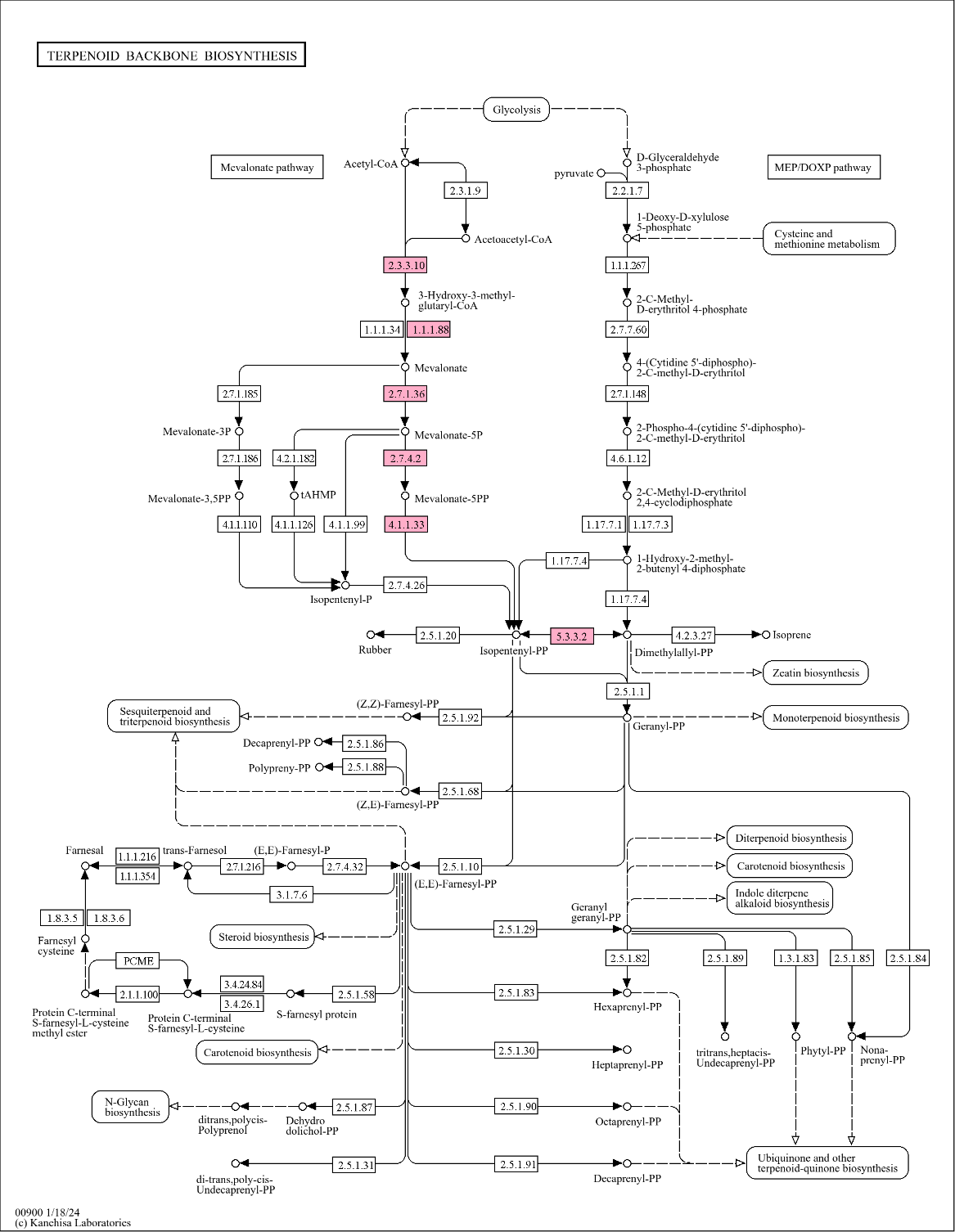
Fig. S64. Enzymatic profile of the "Terpenoid backbone biosynthesis" KEGG pathway in the oral microbiome, exhibiting a ≥ 0.2-fold decrease in abundance in transgenic male littermates expressing amyloid precursor protein/presenilin-1 (APP/PS1) compared to age-matched wild-type controls. EC:2.3.3.10 = Hydroxymethylglutaryl-CoA synthase; EC:2.7.1.36 = Mevalonate kinase; EC:2.7.4.2 = Phosphomevalonate kinase; EC:4.1.1.33 = Diphosphomevalonate decarboxylase; EC:5.3.3.2 = Isopentenyl-diphosphate Delta-isomerase; *EC:1.1.1.88 = Hydroxymethylglutaryl-CoA reductase. The red asterisk (*) symbol functions as a crucial visual marker, highlighting specific KEGG enzymes that have been either substantiated through scientific inquiry or are hypothesized to potentially play pivotal roles in the onset or progression of Alzheimer’s disease (AD). None of the enzymes enriched in this pathway in oral WT microbiome showed increased abundance in the gut microbiome of WT mice relative to APP/PS1 mice. Furthermore, a substantial number of these enzymes were completely undetected in the gut microbial ecosystem. Consequently, these enzymes were not considered from our most recent study examining the influence of the gut microbiome on Alzheimer's disease (AD).


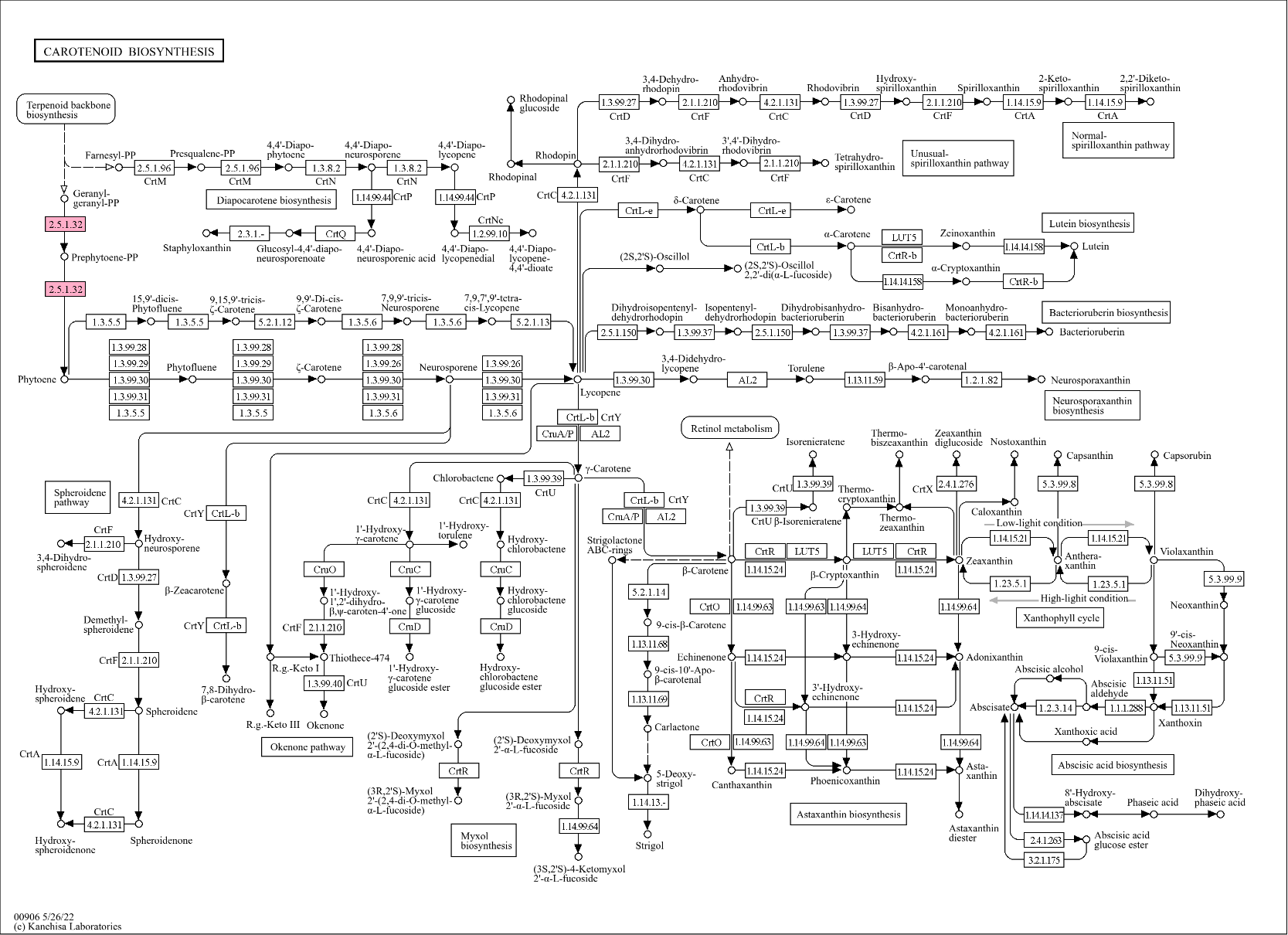
Fig. S65. Enzymatic profile of the "Carotenoid biosynthesis" KEGG pathway in the oral microbiome, exhibiting a ≥ 0.2-fold decrease in abundance in transgenic male littermates expressing amyloid precursor protein/presenilin-1 (APP/PS1) compared to age-matched wild-type controls. EC:2.5.1.32 = 15-cis-phytoene synthase. None of the enzymes enriched in this pathway in oral WT microbiome showed increased abundance in the gut microbiome of WT mice relative to APP/PS1 mice. Furthermore, a substantial number of these enzymes were completely undetected in the gut microbial ecosystem. Consequently, these enzymes were not considered from our most recent study examining the influence of the gut microbiome on Alzheimer's disease (AD).


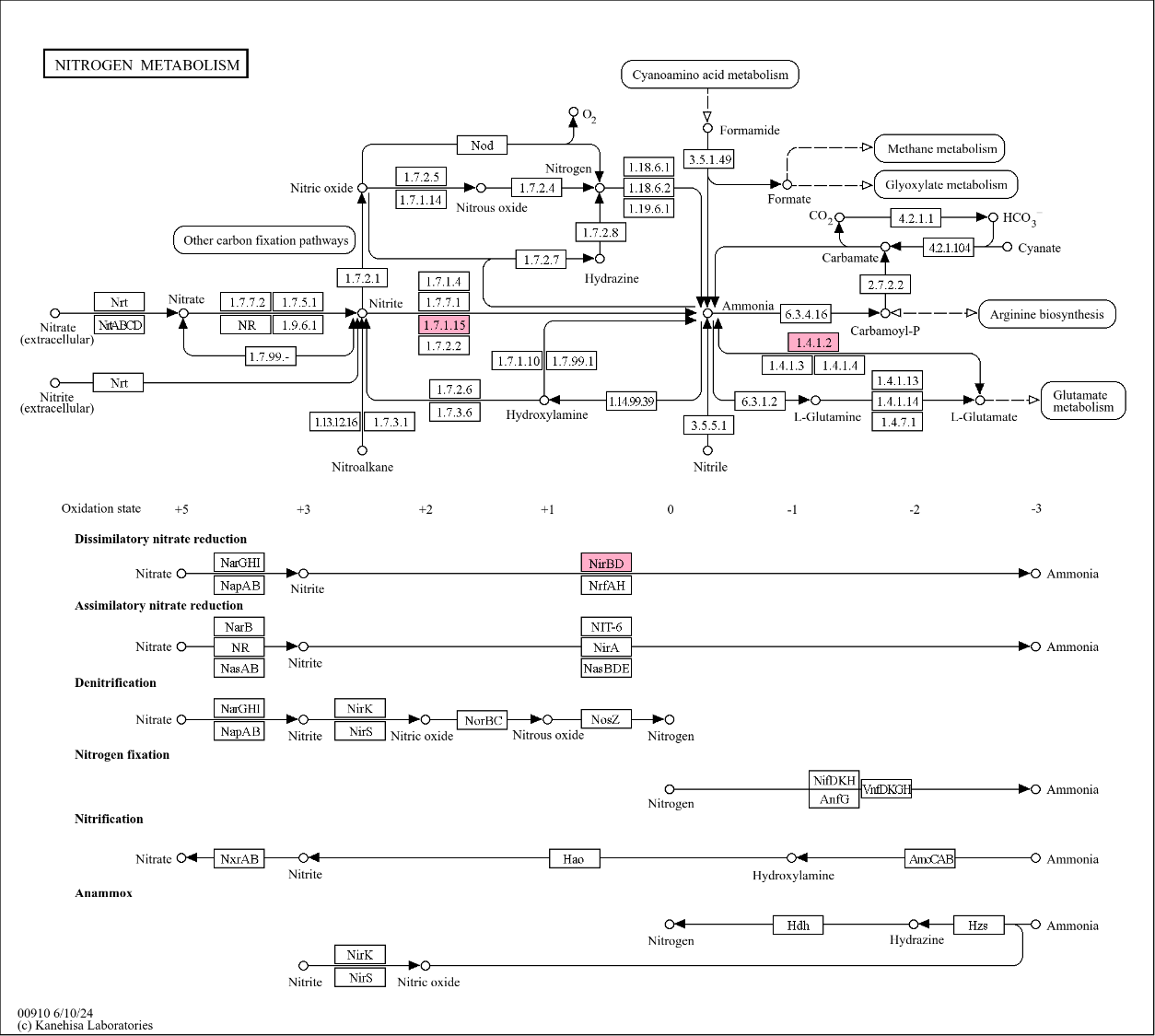
Fig. S66. Enzymatic profile of the "Nitrogen metabolism" KEGG pathway in the oral microbiome, exhibiting a ≥ 0.2-fold decrease in abundance in transgenic male littermates expressing amyloid precursor protein/presenilin-1 (APP/PS1) compared to age-matched wild-type controls. EC:1.7.1.15 = Nitrite reductase (NirBD); *EC:1.4.1.2 = Glutamate dehydrogenase. The red asterisk (*) symbol functions as a crucial visual marker, highlighting specific KEGG enzymes that have been either substantiated through scientific inquiry or are hypothesized to potentially play pivotal roles in the onset or progression of Alzheimer’s disease (AD). None of the enzymes enriched in this pathway in oral WT microbiome showed increased abundance in the gut microbiome of WT mice relative to APP/PS1 mice. Furthermore, a substantial number of these enzymes were completely undetected in the gut microbial ecosystem. Consequently, these enzymes were not considered from our most recent study examining the influence of the gut microbiome on Alzheimer's disease (AD).


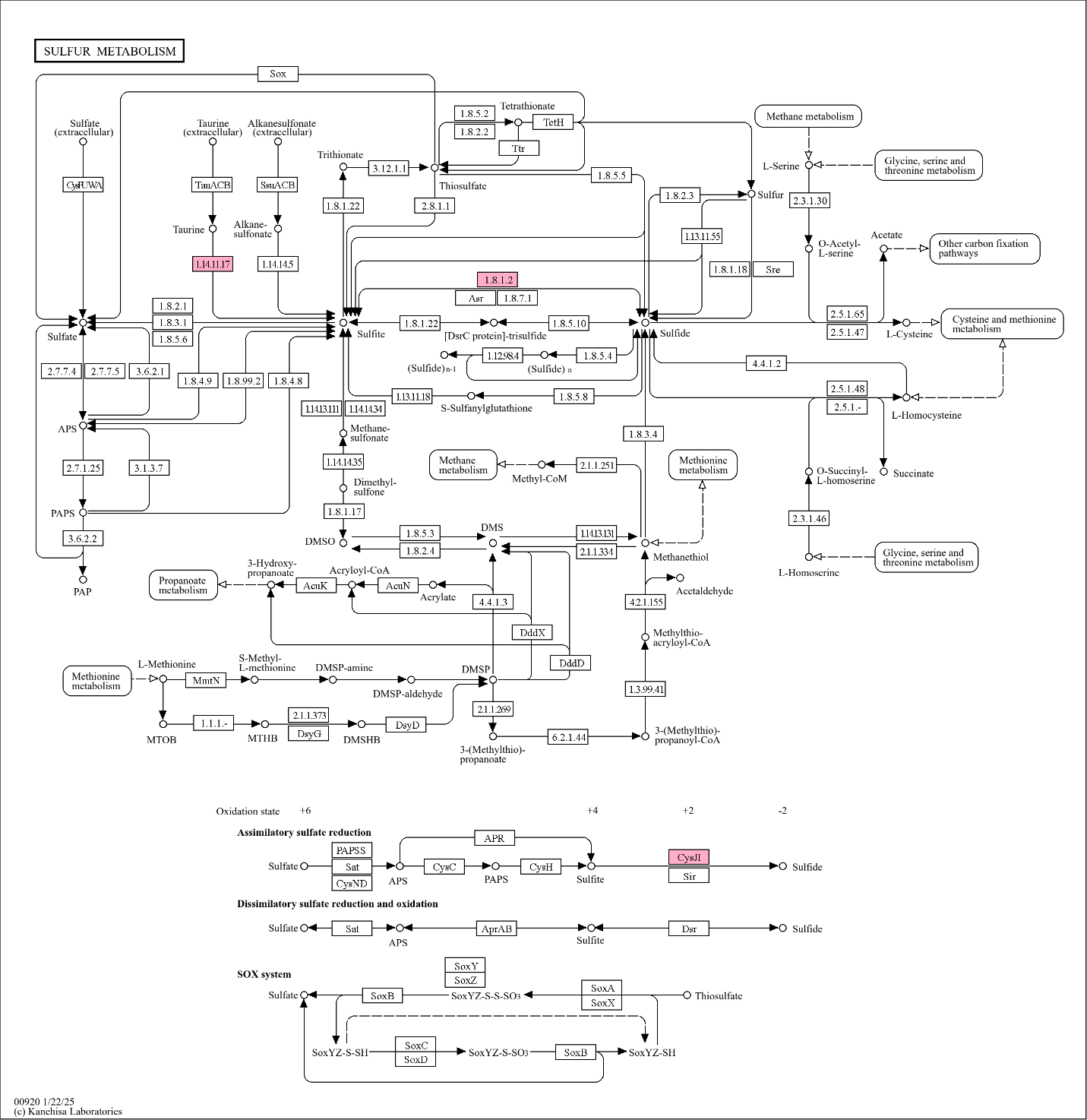
Fig. S67. Enzymatic profile of the "Sulfur metabolism" KEGG pathway in the oral microbiome, exhibiting a ≥ 0.2-fold decrease in abundance in transgenic male littermates expressing amyloid precursor protein/presenilin-1 (APP/PS1) compared to age-matched wild-type controls. EC:1.14.11.17 = Taurine dioxygenase; EC:1.8.1.2 = Assimilatory sulfite reductase (CysJI). None of the enzymes enriched in this pathway in oral WT microbiome showed increased abundance in the gut microbiome of WT mice relative to APP/PS1 mice. Furthermore, a substantial number of these enzymes were completely undetected in the gut microbial ecosystem. Consequently, these enzymes were not considered from our most recent study examining the influence of the gut microbiome on Alzheimer's disease (AD).


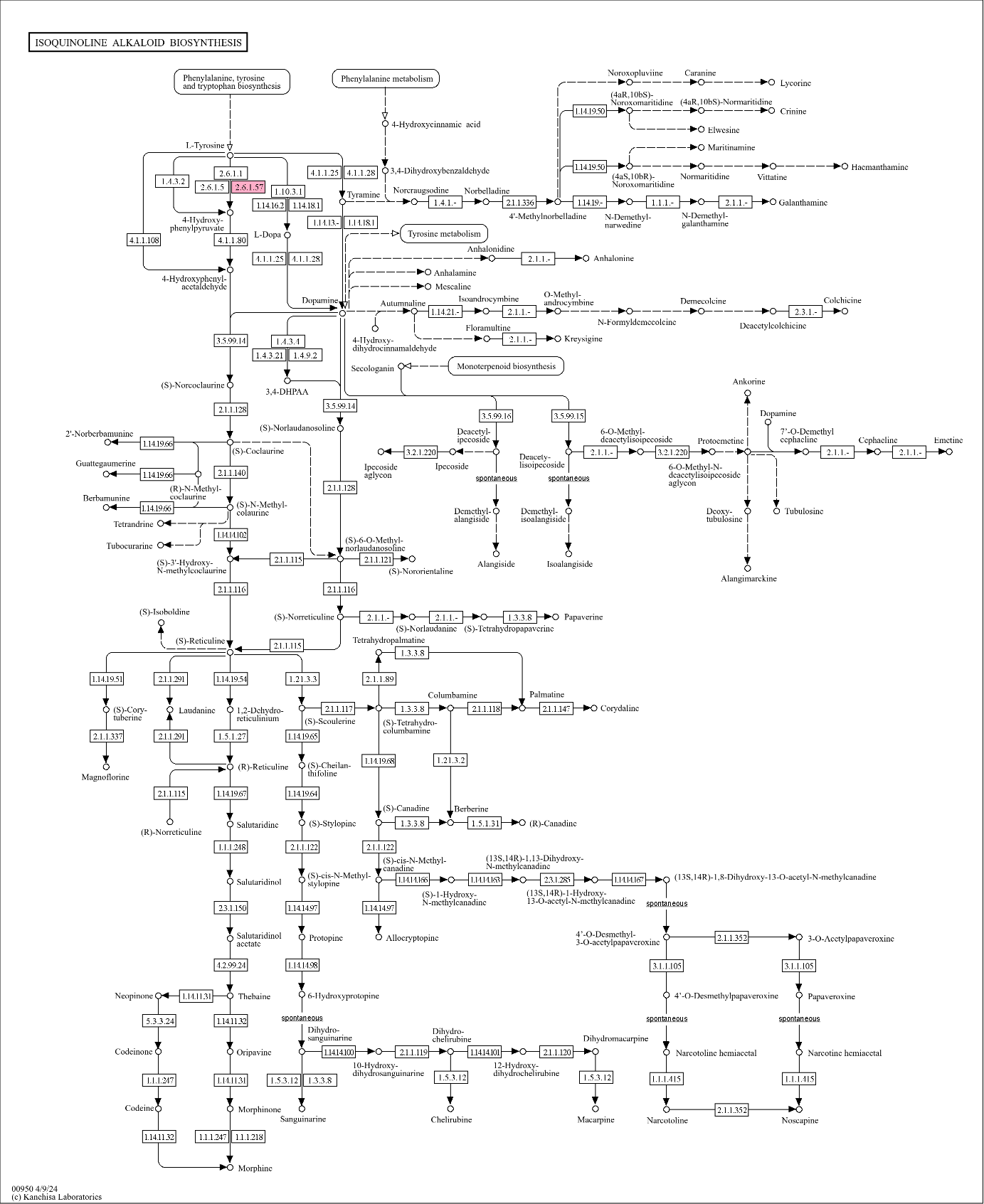
Fig. S68. Enzymatic profile of the "Isoquinoline alkaloid biosynthesis" KEGG pathway in the oral microbiome, exhibiting a ≥ 0.2-fold decrease in abundance in transgenic male littermates expressing amyloid precursor protein/presenilin-1 (APP/PS1) compared to age-matched wild-type controls. EC:2.6.1.57 = Aromatic-amino-acid transaminase. None of the enzymes enriched in this pathway in oral WT microbiome showed increased abundance in the gut microbiome of WT mice relative to APP/PS1 mice. Furthermore, a substantial number of these enzymes were completely undetected in the gut microbial ecosystem. Consequently, these enzymes were not considered from our most recent study examining the influence of the gut microbiome on Alzheimer's disease (AD).


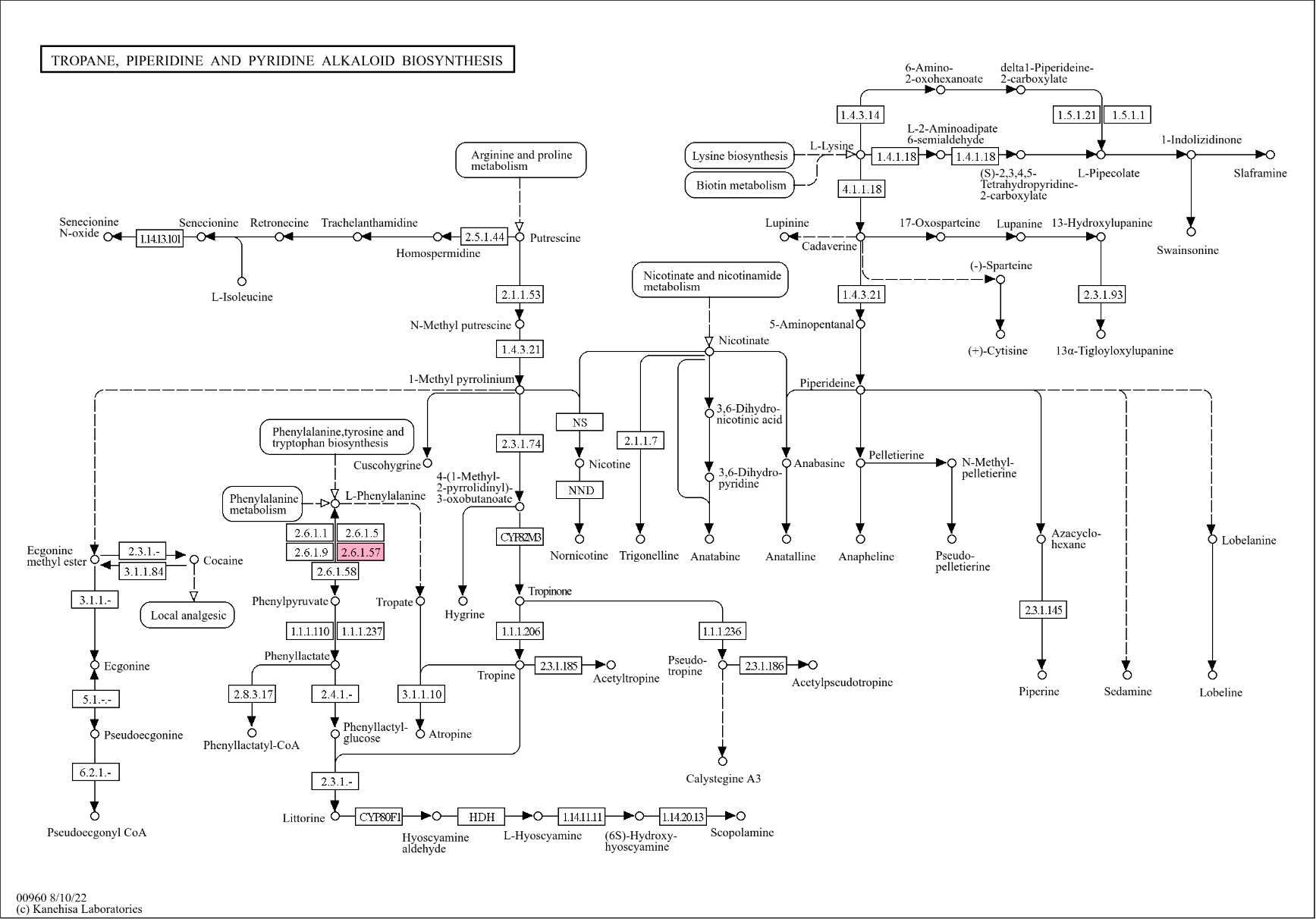
Fig. S69. Enzymatic profile of the "Tropane, piperidine and pyridine alkaloid biosynthesis" KEGG pathway in the oral microbiome, exhibiting a ≥ 0.2-fold decrease in abundance in transgenic male littermates expressing amyloid precursor protein/presenilin-1 (APP/PS1) compared to age-matched wild-type controls. EC:2.6.1.57 = Aromatic-amino-acid transaminase. None of the enzymes enriched in this pathway in oral WT microbiome showed increased abundance in the gut microbiome of WT mice relative to APP/PS1 mice. Furthermore, a substantial number of these enzymes were completely undetected in the gut microbial ecosystem. Consequently, these enzymes were not considered from our most recent study examining the influence of the gut microbiome on Alzheimer's disease (AD).

Fig. S70. Enzymatic profile of the "Biosynthesis of unsaturated fatty acids" KEGG pathway in the oral microbiome, exhibiting a ≥ 0.2-fold decrease in abundance in transgenic male littermates expressing amyloid precursor protein/presenilin-1 (APP/PS1) compared to age-matched wild-type controls. EC:1.14.19.1 = Stearoyl-CoA 9-desaturase (reaction shown in red arrow). None of the enzymes enriched in this pathway in oral WT microbiome showed increased abundance in the gut microbiome of WT mice relative to APP/PS1 mice. Furthermore, a substantial number of these enzymes were completely undetected in the gut microbial ecosystem. Consequently, these enzymes were not considered from our most recent study examining the influence of the gut microbiome on Alzheimer's disease (AD).


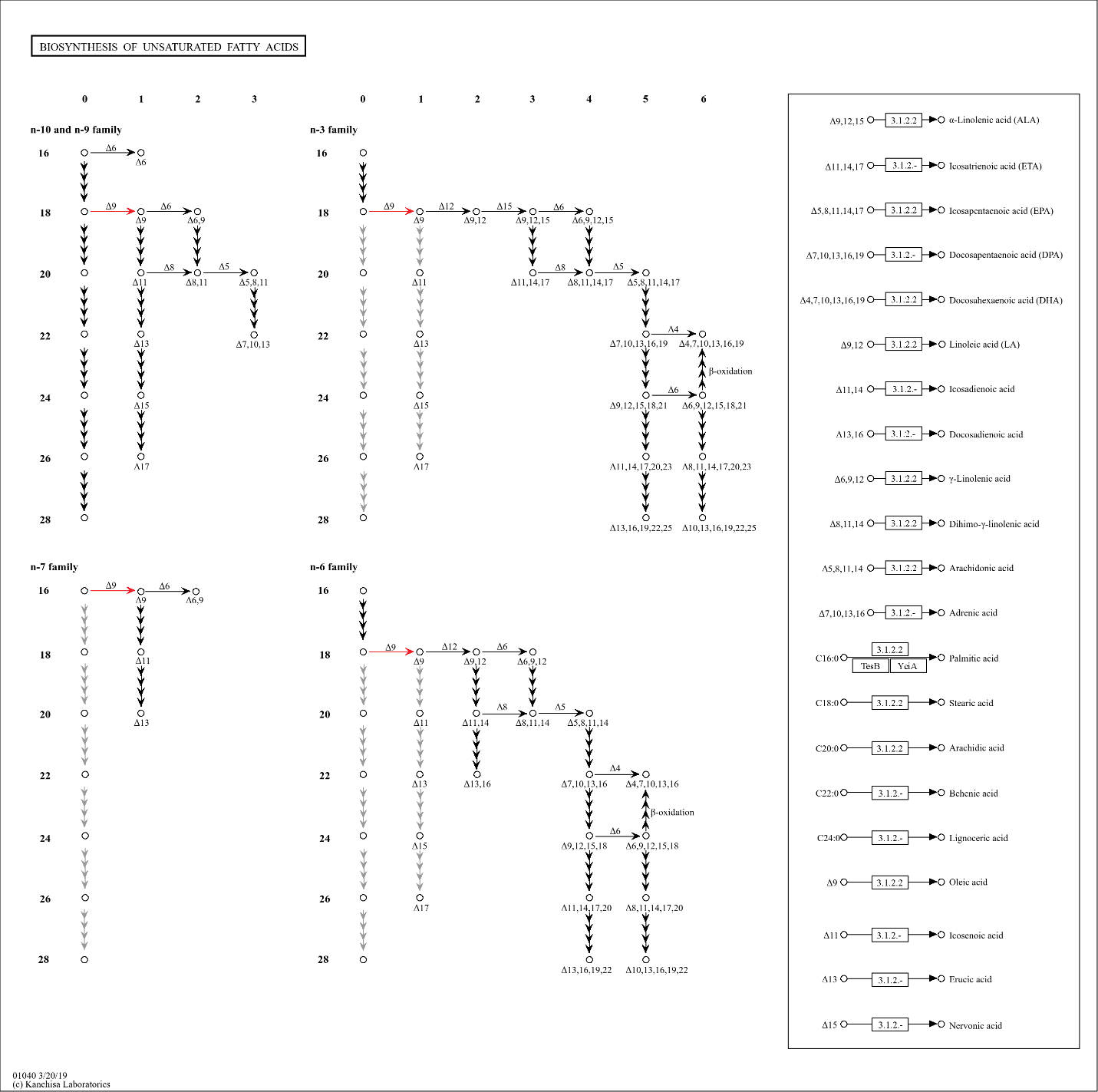


**EC:1.14.19.1**

**EC:1.14.19.1**

**EC:1.14.19.1**

**EC:1.14.19.1**

Fig. S71. Enzymatic profile of the "Alzheimer disease" KEGG pathway in the oral microbiome, exhibiting a ≥ 0.2-fold decrease in abundance in transgenic male littermates expressing amyloid precursor protein/presenilin-1 (APP/PS1) compared to age-matched wild-type controls. *EC:3.4.24.11 (K01389) = Neprilysin (neutral endopeptidase or NEP)
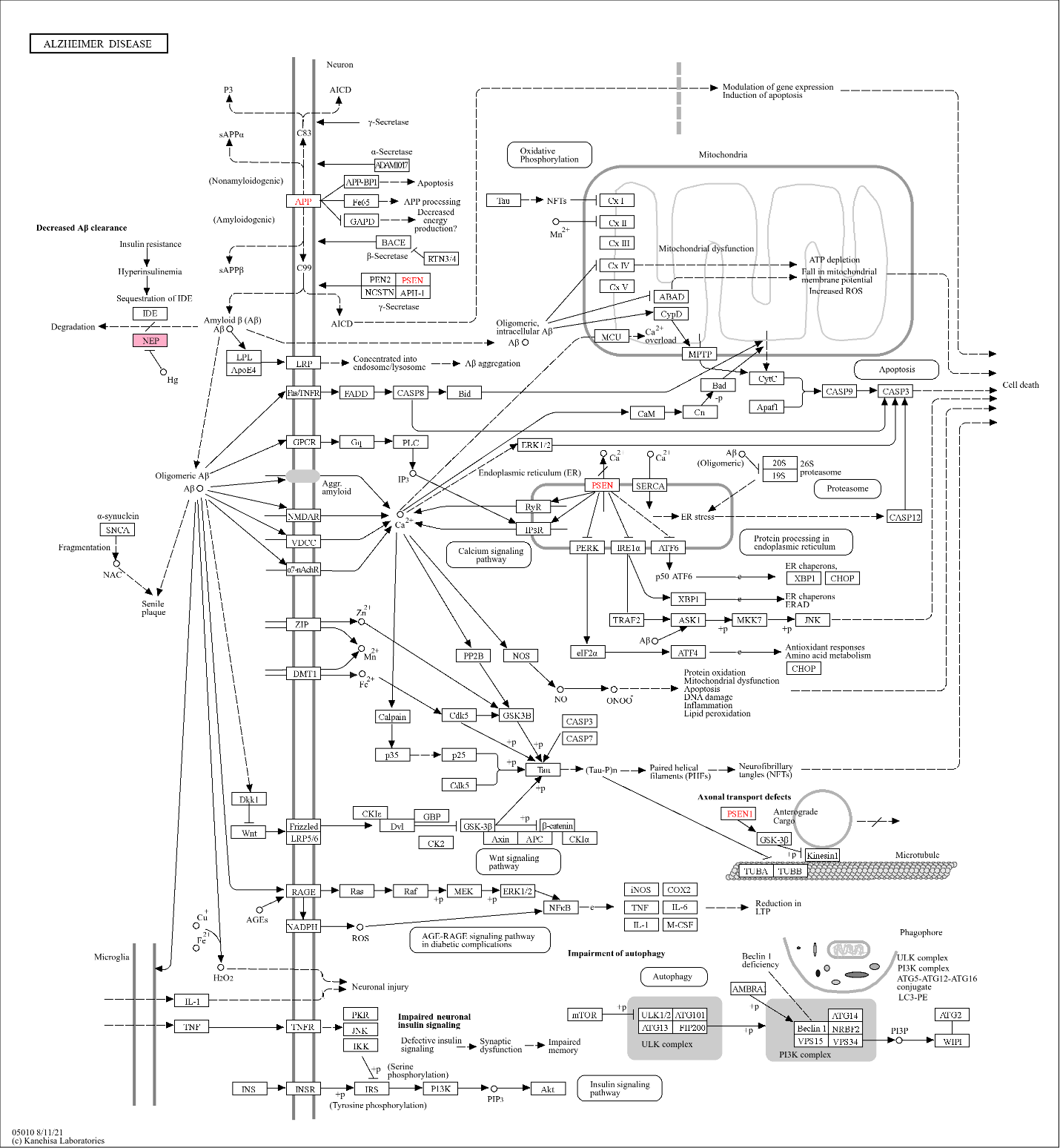
. The red asterisk (*) symbol functions as a crucial visual marker, highlighting specific KEGG enzymes that have been either substantiated through scientific inquiry or are hypothesized to potentially play pivotal roles in the onset or progression of Alzheimer’s disease (AD). None of the enzymes enriched in this pathway in oral WT microbiome showed increased abundance in the gut microbiome of WT mice relative to APP/PS1 mice. Furthermore, a substantial number of these enzymes were completely undetected in the gut microbial ecosystem. Consequently, these enzymes were not considered from our most recent study examining the influence of the gut microbiome on Alzheimer's disease (AD).
